# Identification of Key Genes Involved in Sesquiterpene Synthesis in *Nardostachys jatamansi* Based on Transcriptome and Component Analysis

**DOI:** 10.3390/genes15121539

**Published:** 2024-11-28

**Authors:** Xiaohui Tang, Tingju Li, Zhiyu Hao, Wenji Zhao, Yanlong Han, Guofu Jia, Zhengjun He, Chaoxiang Ren, Ke Rao, Jin Pei, Jiang Chen

**Affiliations:** 1 Sichuan Academy of Grassland Sciences, Chengdu 611731, China; cdzyytxh@163.com (X.T.); li1161112896@163.com (T.L.); zhaowenji2005@163.com (W.Z.); 18298373639@163.com (Y.H.); guo70520@163.com (G.J.); 13350089486@163.com (Z.H.); 2 State Key Laboratory of Southwestern Chinese Medicine Resources, College of Pharmacy, Chengdu University of Traditional Chinese Medicine, Chengdu 611137, China; zzyhh@stu.cdutcm.edu.cn (Z.H.); chaoxiangren@cdutcm.edu.cn (C.R.); 17381096771@163.com (K.R.); peixjin@163.com (J.P.)

**Keywords:** *Nardostachys jatamansi*, transcriptome, sesquiterpene synthase, terpene synthase genes, MeJA

## Abstract

**Background:** *Nardostachys jatamansi* (D. Don) DC. (*N. jatamansi*.) is an endangered medicinal plant native to the Himalayas that is widely used in traditional medicine due to its terpenoid compounds, especially sesquiterpenes, which are abundant in *N. jatamansi*. However, the mechanism of sesquiterpene metabolism remains unclear. **Methods:** Transcriptome sequencing analyses of different parts (roots and rhizomes, leaves, anthocaulus and flowers) and developmental stages (rejuvenation, budding, flowering, fruiting and withering) of cultivated *N. jatamansi* were conducted using the Illumina platform. Key genes involved in regulating the sesquiterpene metabolism pathway in *N. jatamansi* were identified by combining component analyses of various tissues and developmental stages. Furthermore, these key genes were validated through MeJA treatment and a chemical composition analysis. **Results:** A transcriptome sequencing analysis was performed on 24 samples from four tissues and in five developmental stages, yielding 183.18 Gb of clean data with a Q30 base percentage of 92% or above. A total of 269,136 UniGenes were obtained and annotated. Genes related to sesquiterpene synthesis were screened and validated by RT–qPCR using annotation results from various databases. Twelve candidate genes involved in sesquiterpene synthase were identified. Following MeJA treatment, an RT–qPCR analysis revealed that the expression of the *NjTPS-49, NjTPS-54, NjTPS-56, NjTPS-57* and *NjTPS-59* genes was positively regulated. Additionally, an HPLC analysis indicated an increase in the nardosinone content after MeJA treatment. This study demonstrates that *NjTPS-49, NjTPS-54, NjTPS-56, NjTPS-57* and *NjTPS-59* are potential candidate genes for sesquiterpene synthesis. **Conclusion:** The obtained findings establish the groundwork for elucidating the mechanism of sesquiterpene synthesis in *N. jatamansi* and contribute to the conservation of *N. jatamansi* resources.

## 1. Introduction

*N. jatamansi*, commonly known as traditional Chinese medicine, is a perennial herb with a distinctive fragrance. This medicine is widely distributed in Nepal, Bhutan, China, and the Himalayan regions of India at altitudes ranging from 3000 m to 5000 m [1]. In China, *N. jatamansi* is mainly found in high-altitude shrubs and grasslands in Tibet, Sichuan, Gansu, Qinghai and Yunnan, amongst other regions [2]. This medicine is extensively utilised in various traditional medical systems, including Chinese, Islamic, Ayurveda and Korean folk medicine [3,4]. The roots and rhizomes of *N. jatamansi* are commonly used as medicinal parts and can be used to treat mental disorders [5], gastrointestinal discomfort [6], cardiovascular diseases [7] and skin problems [8]. Li et al. found that *N. jatamansi*, combined with levodopa, is a promising therapeutic candidate for PD, providing a scientific basis for the subsequent clinical combination therapy of levodopa to enhance the anti-PD effect [9]. The primary medicinal compound of this medicine, namely nardosinone, exhibits anti-inflammatory and anti-hypertrophic effects on myocardial cells, enhances the activity of nerve growth factor and promotes the proliferation and differentiation of neural stem cells [10].

*N. jatamansi* is a highly renowned medicinal and aromatic plant in the Himalayan region. In addition to its medicinal uses, this plant is also a source of fragrance used in incense, flavouring agents and high-quality perfumes, and it is widely applied in the cosmetics, food and pharmaceutical industries. Terpenoid compounds are the main components responsible for the aromatic fragrance of many flowering plants, including *Lavandula officinalis* [11], *Lilium ‘siberia’* [12], *Jasminum sambac* [13] and *Osmanthus fragrans Lour.* [14], amongst others. Similarly, the primary constituents of *N. jatamansi* are also terpenoids [3], which likely contribute to its unique aroma that attracts pollinating insects [15] and serves as a protective mechanism against grazing by plateau animals such as yaks, sheep and plateau pikas [16]. Additionally, sesquiterpenoids are important quality evaluation indicators for *N. jatamansi* medicinal materials [17]. This species is experiencing a decline in population and is protected under the Convention on International Trade in Endangered Species of Wild Fauna and Flora (CITES) due to overexploitation, particularly of its medicinal parts (roots and rhizomes). Scientific research on *N. jatamansi* is limited due to its remote habitat and sparse populations. Investigating the metabolic pathways of its active terpenoid compounds is crucial for protecting wild *N. jatamansi* resources, alleviating supply pressures and establishing the foundation for the artificial biosynthesis of terpenoids.

Research on the metabolic pathways of terpenoids in *N. jatamansi* is currently limited. Nisha Dhiman et al. [18] analysed the genes involved in the biosynthesis pathways of phenolic compounds in *N. jatamansi* through de novo transcriptome sequencing. Additionally, Mingkang Feng et al. examined the differences in transcriptomes and metabolomes of different tissues of *N. jatamansi* and identified some terpenoid synthase genes [19]. However, scientific research on the molecular mechanism of terpenoid metabolism pathways in *N. jatamansi* remains limited.

Terpenoid compounds in living organisms are synthesised using the following two main pathways [20]: the mevalonic acid (MVA) pathway and the 2-methyl-D-erythritol-4-phosphate (MEP) pathway. In the cytoplasm, acetyl-CoA (AC) is fixed to form acetoacetyl-CoA (ACC), which is catalysed by AACT to condense two AC molecules into one ACC molecule. ACC and AC are further condensed by 3-hydroxy-3-rnethylglutaryl-CoA synthase to form 3-hydroxy-3-methylglutaryl-CoA, which is then reduced by the rate-limiting enzyme 3-hydroxy-3-methylglutaryl-CoA reductase to yield mevalonate (MVA). Subsequently, MVA is converted into mevalonate-5-phosphate (MVAPP), a process mediated by the phosphorylation actions of mevalonate kinase and phosphomevalonate kinase (PMK). MVAPP undergoes further enzyme-catalysed reactions, such as decarboxylation by mevalonate 5-diphosphate decarboxylase, to form isopentenyl diphosphate (IPP). Along with its isomer dimethylallyl diphosphate, IPP generates farnesyl diphosphate, which is further converted into sesquiterpenes and triterpenoid compounds through terpene synthase catalysis.

In this study, the Illumina platform was used to conduct transcriptome sequencing analyses of different tissues (roots and rhizomes, leaves, anthocaulus and flowers) and different developmental stages (rejuvenation, budding, flowering, fruiting and withering) of cultivated *N*. *jatamansi.* Genes involved in terpenoid biosynthesis were screened to analyse their expression patterns. The results of this study not only establish the foundation for elucidating the metabolic pathways of sesquiterpenes in *N*. *jatamansi* but also provide valuable resources for further research on the artificial synthesis of sesquiterpene components, thereby reducing usage of the original plant and protecting wild resources of *N*. *jatamansi*.

## 2. Materials and Methods

### 2.1. Plant Materials

**Figure 1:**
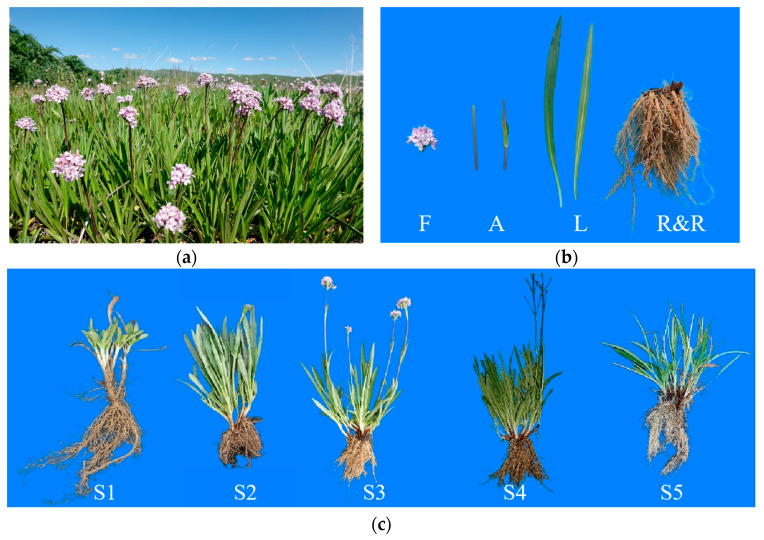
Plant materials. (**a**) Whole plant of cultivated *N. jatamansi.* (**b**) Four tissues of N. *jatamansi* (flowers [F]; anthocaulus [A]; leaves [L]; roots and rhizomes [R&R]). (**c**) Developmental stages of *N. jatamansi* (rejuvenation stage [S1], budding stage [S2], flowering stage [S3], fruiting stage [S4] and withering stage [S5]).

The plant material used in this study comprised three-year-old seedlings of *N. jatamansi* (Figure 1a) cultivated at the Hongyuan Scientific Research Base of Sichuan Academy of Grassland Sciences, Sichuan Province, China, with consistent genetic backgrounds. Samples were collected from April 2023 to September 2023, including four different tissues during the flowering stage (roots and rhizomes, leaves, anthocaulus and flowers) (Figure 1b), as well as roots and rhizomes at different developmental stages (rejuvenation, budding, flowering, fruiting and withering) (Figure 1c). Each sample had three biological replicates, with each replicate comprising tissue from at least five plants of the same part, which were immediately frozen in liquid nitrogen for transcriptome sequencing and expression analysis.

### 2.2. RNA Extraction and Quality Assessment

RNA extraction from plant samples was performed using the TRansZol Up Plus RNA Kit (Q41206, TransGen, Beijing, China) following the manufacturer’s instructions. The integrity of the extracted RNA and the presence of DNA contamination were analysed using agarose gel electrophoresis to ensure the accuracy of sequencing data. RNA purity was assessed using a Nano Photometer spectrophotometer (IMPLEN, Westlake Village, CA, USA), and RNA concentration was accurately quantified using the Qubit 2.0 fluorometer (Life Technologies, Carlsbad, CA, USA). Additionally, RNA integrity was precisely assessed using the Agilent 2100 bioanalyser (Agilent Technologies, Santa Clara, CA, USA).

### 2.3. RNA-Seq Transcriptome Sequencing

Library construction was carried out with the Illumina NEBNext^®^ UltraTM RNA Library Prep Kit (NEB, Ipswich, MA, USA). Following library construction, initial quantification was performed using the Qubit 2.0 fluorometer, and the libraries were diluted to 1.5 ng/μL. Subsequently, the insert size of the libraries was assessed using the Agilent 2100 bioanalyser (Agilent Technologies, Santa Clara, CA, USA). After confirming that the insert size met expectations, qRT-PCR was conducted to accurately quantify the effective concentration of the libraries (effective concentration above 2 nM) and ensure library quality.

Following library qualification, different libraries were pooled based on their effective concentrations and the required amount of target sequencing data. Illumina sequencing was performed, generating paired-end reads of 150 bp. The sequencing principle involved sequencing via synthesis, where four fluorescently labelled dNTPs, DNA polymerase and adapter primers were added to the flow cell for amplification. With the extension of each complementary strand in each sequencing cluster, the incorporation of a fluorescently labelled dNTP released a corresponding fluorescence signal. The sequencer captured these signals and converted them into sequencing peaks using a computer software, thereby obtaining the sequence information of the target fragments.

The raw sequencing data underwent filtering using fastp [21], primarily removing reads containing adapters. Reads were further filtered out if the N content exceeded 10% of the base count or if the number of low-quality bases (Q <= 20) exceeded 50% of the read length. All subsequent analyses were based on the clean reads.

Transcriptome assembly of clean reads was performed using Trinity [22], and the assembled transcripts were clustered and de-replicated using Corset (https://github.com/trinityrnaseq/trinityrnaseq, accessed on 2 September 2023). SSR analysis was performed on UniGenes from different tissues using MISA [23].

### 2.4. Gene Functional Annotation and Differential Gene Screening

Using DESeq2 1.22.2 software [24,25], differential expression analysis was conducted between sample groups to obtain a set of differentially expressed genes between two biological conditions. The input data for differential analysis comprised unnormalised read count data for genes. Subsequently, the Benjamini–Hochberg method was employed to adjust the hypothesis testing probability (*p* value) through multiple hypothesis testing corrections, yielding the false discovery rate (FDR). Differential gene selection criteria were set at │log2Fold Change│ >= 1, with FDR < 0.05. DEGs were then subjected to GO enrichment and KEGG pathway enrichment analyses using Diamond v2.0.9.

### 2.5. Phylogenetic Analysis of Terpenoid Synthesis Genes

After the analysis of differentially expressed genes was completed by using DESeq2 1.22.2, the total number of differentially expressed genes, the number of up-regulated genes and the number of down-regulated genes in each group were counted (Figure S4). Based on the annotation results of UniGenes from different parts and developmental stages in the Nr, SwissProt, TrEMBL and Pfam databases, genes associated with the synthesis of terpenoid compounds were identified. The open reading frames and amino acid sequences of these genes were obtained, resulting in 104 terpene synthase amino acid sequences (Additional File S2). Terpene synthase amino acid sequences reported by Mingkang Feng et al. [19] were selected to have differential expression in various tissues of *N. jatamansi*, as well as several terpene synthase subtypes downloaded from NCBI: TPS-a, TPS-b, TPS-c, TPS-e/f and TPS-g amino acid sequences [26,27,28]. The amino acid sequences of these genes are detailed in Additional File S2. A phylogenetic tree was constructed using the neighbour-joining method based on the 104 terpene synthase sequences obtained from the transcriptome data and those selected sequences. These genes were compiled and aligned using MEGA 7.0 (MEGA, http://www.megasoftware.net/, accessed on 6 May 2024).

### 2.6. Quantitative Real-Time PCR Verification Analysis

qRT–PCR was performed to validate the transcriptional data. Total RNA extracted from various tissue samples was reverse-transcribed into cDNA using the EasyScript@One-step gDNA removal and cDNA Synthesis SuperMix kit (TransGen, Beijing, China). As a commonly used housekeeping gene, actin was chosen as the internal control [19]. All qRT–PCR primers were designed using Primer-blast (https://www.ncbi.nlm.nih.gov/tools/primer-blast/index.cgi?LINK_LOC=BlastHome, accessed on 23 May 2024). Detection was conducted using the Analytik Jena qTower2.2 system (Analytik Jena, Jena, Germany). qRT–PCR was then performed with three biological replicates and three technical replicates using the PerfectStart Green qPCR SuperMix dye-based fluorescence quantitative pre-mixed reagent kit (TransGen, Beijing, China). The PCR protocol was performed as follows: initial denaturation at 95 °C for 30 s (hold stage), followed by denaturation at 95 °C for 5 s and annealing/extension at 57 °C for 30 s (PCR stage), and a melt curve stage comprising denaturation at 95 °C for 15 s, annealing at 60 °C for 60 s and denaturation at 95 °C for 15 s [19]. The 2^^∆∆^Ct method was used to calculate the relative expression levels normalised to the housekeeping gene.

### 2.7. Expression Analysis of Sesquiterpene Synthase Genes Under MeJA Treatment

Methyl jasmonate (MeJA) is widely present in plants, and its exogenous application can trigger the expression of defence-related genes, inducing chemical defences in plants, and it can produce effects similar to those of mechanical damage and insect feeding [29]. The experimental procedure was adapted from a previous study [30] with some modifications. A solution containing 110 μmol/L MeJA (MACKLIN, Shanghai, China) was irrigated into the soil of *N. jatamansi* during the fruiting stage, whilst the control group was irrigated with an equal volume of pure water. Three *N. jatamansi* plants were irrigated for each treatment to minimise potential errors attributed to individual plant variability. After three days of treatment, the roots and rhizomes of *N. jatamansi* plants were collected and immediately frozen in liquid nitrogen and then stored at −80 °C. RNA extraction was performed for each sample using the aforementioned method, and the expression levels of sesquiterpene biosynthesis genes in the roots and rhizomes of *N. jatamansi* before and after MeJA treatment were analysed using the fluorescence quantitative RT–qPCR method.

### 2.8. Chemical Component Analysis

Nardosinone is a key component in *N. jatamansi*. The contents of nardosinone in different tissues, developmental stages and MeJA-treated samples of *N. jatamansi* were determined using LC-2030 3D Plus (Shimadzu, Kyoto, Japan) with nardosinone as the standard. The chromatographic conditions included an octadecylsilane bonded silica column and a mobile phase of acetonitrile/water (65:35) with the detection wavelength set at 254 nm.

## 3. Results

### 3.1. Transcriptome Sequencing and Transcript Assembly

High-quality RNA from four tissues (roots and rhizomes, flower stems, leaves and flowers) and five developmental stages (rejuvenation, budding, full flowering, fruiting and withering) of *N. jatamansi* were extracted to identify as many transcripts as possible. Sequencing was then performed using the Illumina platform, yielding 1422 million raw reads. The adapter sequences were filtered out using fastp, and raw data filtering, sequencing error rate checking and GC content distribution checking were conducted to obtain 1391 million clean reads for a subsequent analysis. The clean data for each sample exceeded 6.4 Gb with a quality score of Q30 > 92.5% and a GC content of >42.2%. The sequence length distribution at four tissues and five developmental stages are shown in Figure S1A,B. A total of 269,136 UniGenes were identified from the different tissues and developmental stages.

### 3.2. Gene Annotation and Functional Classification

The UniGene sequences were annotated against the KEGG, NR, Swiss-Prot, GO, COG/KOG and TrEMBL databases using the DIAMOND BLASTX v2.0.9. Subsequently, the amino acid sequences of the UniGenes were predicted and annotated using the HMMER 3.2 against the Pfam database. The annotation results for different tissues are as follows: KEGG (61,527; 53.5%), NR (79,656; 69.26%), Swiss-Prot (60,548; 52.64%), TrEMBL (79,732; 69.32%), KOG (47,545; 41.34%), GO (70,147; 60.99%) and Pfam (59,543; 51.77%) (Figure S2A). Similarly, the annotation results for different developmental stages are as follows: KEGG (78,542; 50.96%), NR (102,036; 66.2%), SwissProt (75,872; 49.23%), TrEMBL (103,764; 67.33%), KOG (63,523; 41.22%), GO (90,539; 58.74%) and Pfam (77,459; 50.26%) (Figure S2B).

**Figure 2:**
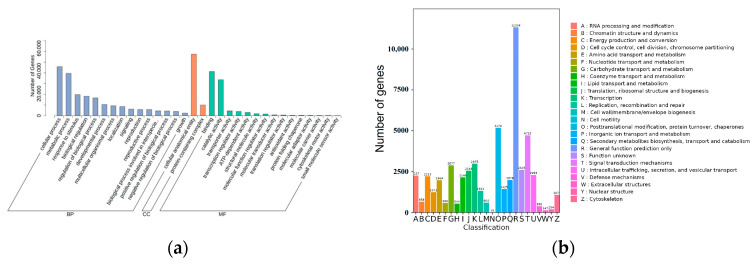
GO and KEGG annotations of de-redundant sequences from tissues. (**a**) GO annotation of de-redundant sequences from all tissues. The full expression of “biological process involved in intraspecies…” in the figure is “biological process involved in intraspecies interaction between organisms”. (**b**) KOG annotation of de-redundant sequences from all tissues.

Functional annotation of genes from different tissues and developmental stages was conducted using the GO database, and the genes were categorised as molecular function (MF), cellular component (CC) and biological process (BP). The majority of genes were annotated under cellular and metabolic processes in the BP category, cellular anatomical entity in the CC category, and binding and catalytic activity in the MF category (Figure 2a).

According to the annotation results from the KOG (euKaryotic Orthologous Groups) database, the largest number of genes were annotated as ‘General function prediction only’, followed by ‘Posttranslational modification, protein turnover, chaperones’ and ‘Signal transduction mechanisms’ (Figure 2b).

By aligning with the NR library, it can be seen that the species with the closest sequence to the transcript of *N. jatamansi* is *Nyssa sinensis* (Figure S3).

### 3.3. Phylogenetic Analysis of Terpene Synthase Genes

**Figure 3:**
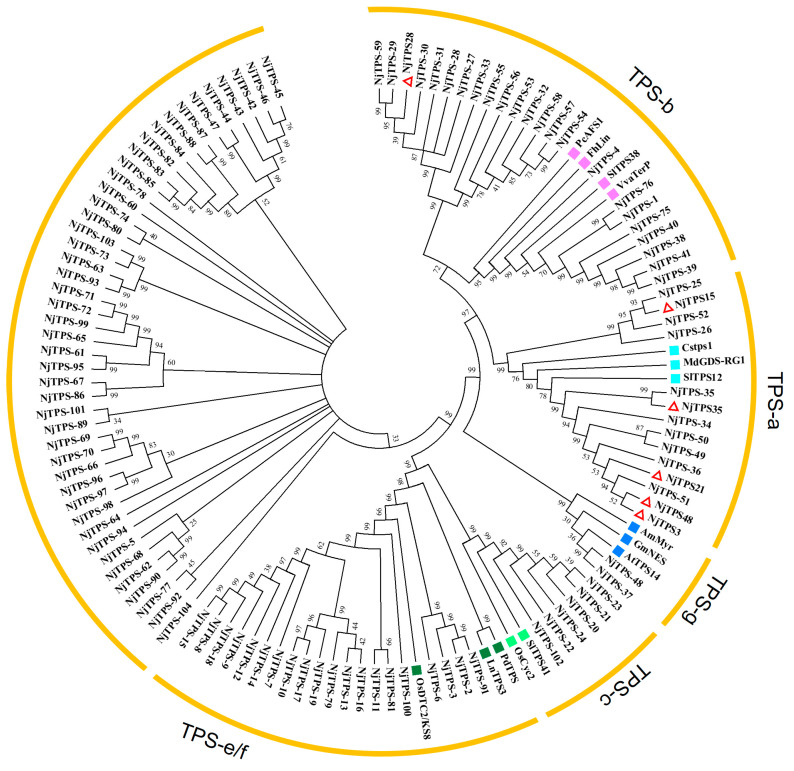
Phylogenetic tree of *NjTPSs*. Different colours represent different terpene synthase subfamilies: ■ TPS-a, ■ TPS-b, ■ TPS-c, ■ TPS-g and ■ TPS-e/f. **△** is the gene reported by Mingkang Feng et al. [19]. Information on TPS genes used for phylogenetic analysis is shown in Table S1.

The phylogenetic tree (Figure 3) indicates that *NjTPS-25*, *NjTPS-29* and *NjTPS-35* are genetically closest to *NjTPS15*, *NjTPS28* and *NjTPS35*. Amongst them, *NjTPS3*, *NjTPS21* and *NjTPS48* are all closest in genetic distance to *NjTPS-51* in this study. The phylogenetic tree shows that these *NjTPSs* are classified into the TPS-a, TPS-b, TPS-c, TPS-g and TPS-e/f subfamilies. The genes classified in the TPS-a subfamily are mostly related to sesquiterpene metabolism, and these genes will be the focus of this study. However, some genes are not classified into the five major terpene subfamilies, and further research is necessary to confirm the functions of these genes.

### 3.4. Gene Expression Pattern Validation and Screening of Sesquiterpene Synthase Genes

**Figure 4:**
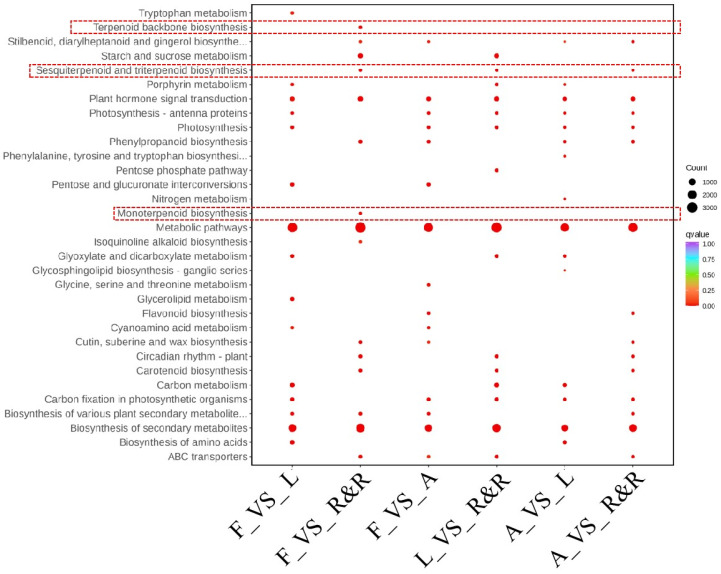
A comparison KEGG_Enrichment Bubble. The abscissa is the comparative combination; the ordinate is the enrichment pathway; the size of the point represents the number of genes enriched to the pathway; the larger the point, the more genes are enriched to the pathway; the colour of the point represents the significance value of the enrichment to the pathway; and the redder the colour of the point, the more significant the enrichment. The red dashed box represents the situation of the enriched genes in the terpene metabolic pathway for each comparative combination.

Based on the annotation results of the samples from four tissues in the database, as well as the differential expression of genes in the KEGG metabolic pathways, a comparison KEGG Enrichment Bubble plot was generated (Figure 4). Notably, pathways such as terpenoid backbone biosynthesis, sesquiterpenoid and triterpenoid biosynthesis, and monoterpenoid biosynthesis exhibit differential expression primarily in the roots. This finding indicates that sesquiterpenes are predominantly expressed in the roots and rhizomes, which is consistent with the terpenoid analysis results for the roots and rhizomes reported in reference [19], revealing that ‘the roots and rhizomes have the highest diversity and content of terpenoids’. Therefore, this study extensively screened genes related to sesquiterpene synthesis and analysed the expression patterns of terpenoid synthesis genes in different parts as well as during different developmental stages of roots and rhizomes.

**Figure 5:**
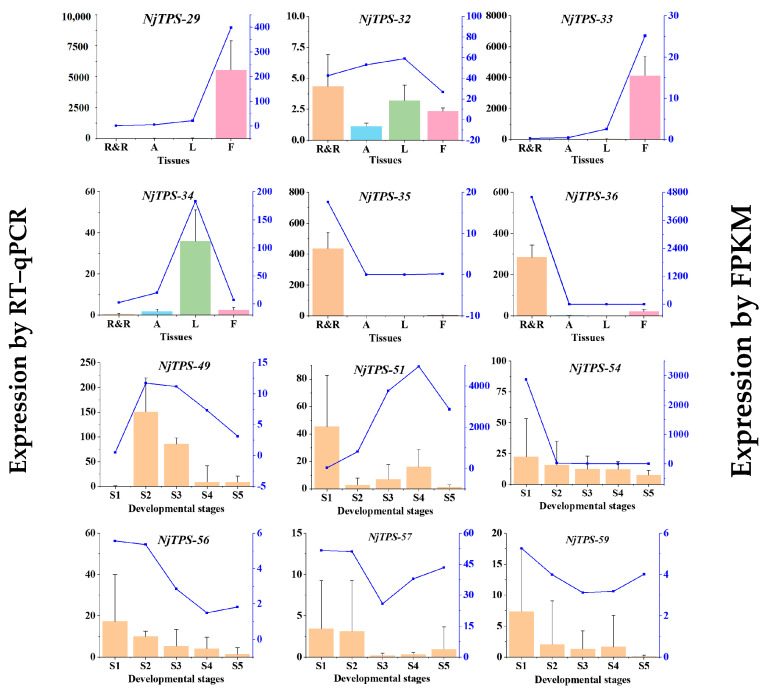
Partial validation of putative *NjTPSs* by RT–qPCR.

Based on the FPKM values of various UniGenes in the transcriptome data, as well as the annotation results in the KEGG pathways Ko00900 and Ko00909 and the annotation results in the Pfam database, 12 genes involved in sesquiterpene biosynthesis differential expression were screened across different tissues and developmental stages. RT–qPCR was employed to detect the expression level differences in sesquiterpene synthesis-related genes in different tissues and developmental stages to validate the accuracy of the transcriptome data. The primer sequences can be found in Table S2. The results show that the expression trends of the validated genes in various tissues and developmental stages were mostly consistent with the transcriptome data, indicating the reliability of the transcriptome data (Figure 5). Amongst them, *NjTPS-32, 35* and *36* showed the highest expression levels in the roots. Meanwhile, *NjTPS-34* revealed the highest expression levels in the leaves, and *NjTPS-29* and *33* exhibited the highest expression levels in the flowers.

### 3.5. Expression Analysis of Sesquiterpenoid Biosynthesis Genes Under MeJA Treatment

**Figure 6:**
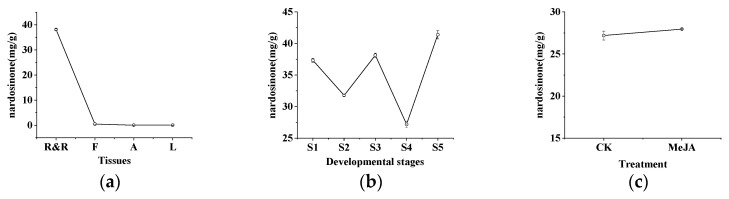
Results of HPLC determination of nardosinone. (**a**) Content of nardosinone in four tissues. (**b**) Content of nardosinone in five developmental stages. (**c**) Content of nardosinone in roots and rhizomes under MeJA treatment (CK indicates that MeJA is not used).

The content of nardosinone in different tissues, at different developmental stages and before and after the MeJA treatment of the roots and rhizomes of cultivated *N. jatamansi* was measured using HPLC to further validate the function of terpenoid synthesis genes (Figure 6). The nardosinone content was the highest in the roots and rhizomes, which corresponds to the expression trends of *NjTPS-32*, *NjTPS-35* and *NjTPS-36* (Figure 5). The results reveal that the nardosinone content peaked during the withering stage, followed by the flowering stage, and it was the lowest during the fruiting stage. The nardosinone content increased after the MeJA treatment.

**Figure 7:**
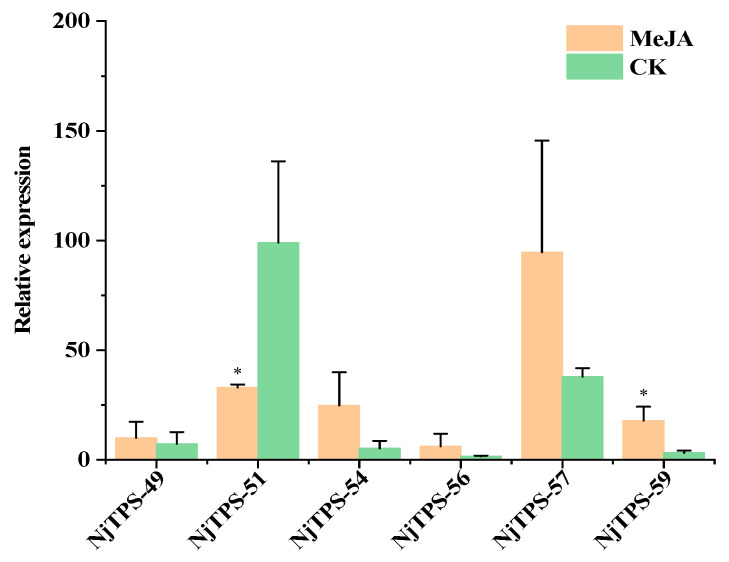
Temporal expression analyses of sesquiterpene biosynthesis genes under MeJA treatment. The significance of the differences was analysed using a one-sided paired *t*-test (* *p* < 0.05).

During the fruiting stage, MeJA was applied to the soil of three individually cultivated *N. jatamansi* plants. RNA was extracted from the samples before and after treatment for further validation of the sesquiterpene synthesis genes. The results (Figure 7) show that the *NjTPS-49*, *NjTPS-54*, *NjTPS-56, NjTPS-57* and *NjTPS-59* genes were up-regulated, whilst the *NjTPS-51* gene was down-regulated. *NjTPS-49, NjTPS-54, NjTPS-56, NjTPS-57* and *NjTPS-59* showed a positive regulatory effect on nardosinone synthesis, indicating the potential involvement of these genes in the synthesis of nardosinone.

### 3.6. SSR Identification

Molecular marker-assisted breeding is frequently employed to conserve wild plant resources and select high-quality varieties. Compared to other molecular marker technologies, simple sequence repeats (SSRs) are high-polymorphism markers widely distributed within genomes. SSRs have the advantages of co-dominance and repeatability and are extensively used in cultivar identification, phylogenetic studies, genetic diversity research, the construction of genetic linkage maps and molecular marker-assisted breeding. SSR molecular markers can be used for resource screening. This study utilised MISA to perform an SSR analysis on UniGenes from different tissues, identifying six types of SSRs, namely mono-nucleotide, di-nucleotide, tri-nucleotide, tetra-nucleotide, penta-nucleotide and hexa-nucleotide repeats, with the following quantities: 15,664, 15,193, 10,038, 754, 299 and 345, respectively (Figure S5A). The SSR analysis of the five developmental stages revealed the following quantities for the six types: 19,645, 18,028, 11,541, 838, 287 and 453. The results indicate the abundance of mono-nucleotide repeat SSRs (Figure S5B). The identified SSR sequences can be further screened, and the identification of SSR sequences in this study provides fundamental data for the future development of specific SSR molecular markers.

## 4. Discussion

*N. jatamansi* has a long history of use as a medicine and spice, and current research primarily focuses on the pharmacological effects and structures of terpenoids [31]. In this research, the medicinal parts of *N. jatamansi*, namely the roots and rhizomes, are treated as a single tissue for study. This approach is adopted because the two parts are generally not separated in practical production. Additionally, all editions of the *Pharmacopoeia of the people’s Republic of China* (Ch.P) specify that the medicinal parts of *N. jatamansi* are the roots and rhizomes.

This study compares the results with the terpenoid synthases reported by Mingkang Feng et al. [19], revealing a high degree of similarity for most genes. In addition to a transcriptome analysis of different tissues of *N. jatamansi*, this research also involves a transcriptome analysis of the roots and rhizomes at several developmental stages and the further identification of sesquiterpene synthases using MeJA treatment. The addition of transcriptome sequencing for roots and rhizomes at different developmental stages revealed a notable increase in the number of terpenoid synthase genes, indicating that the roots and rhizomes of *N. jatamansi* are highly regulated by terpenoid synthases during growth. However, this study only treated the roots and rhizomes with MeJA without optimising the treatment concentration or separately treating other parts, such as flowers and leaves. Based on the current experimental results, only two sesquiterpene synthase genes exhibited considerable differential expression before and after MeJA treatment. This finding may be related to the treatment concentration or could indicate a minor effect of MeJA on sesquiterpene expression in *N. jatamansi*. In future work, our research group will conduct further functional validation of the identified sesquiterpene synthase genes.

## 5. Conclusions

In our study, the Illumina platform was used to sequence the four tissues and five developmental stages of the *N. jatamansi,* and 183.18 Gb of clean data were obtained. A total of 269,136 UniGenes were acquired and annotated. Genes related to sesquiterpene synthesis were screened and validated using RT–qPCR through annotation results in various databases. Twelve candidate genes involved in sesquiterpene synthesis were identified. Following MeJA treatment, five terpenoid synthases may be involved in the synthesis of sesquiterpenoids. The SSR was also analysed in this study. Overall, the results provide a valuable resource for further research on *N. jatamansi*.

## Data Availability

The original contributions presented in this study are publicly available. These data can be found at NCBI, NGS sequencing PRJNA1131859.

## Supplementary Materials

The following supporting information can be downloaded at https://www.mdpi.com/article/10.3390/genes15121539/s1. Additional File S1. This file includes all additional figures and tables (Figures S1–S5; Tables S1 and S2) used in this manuscript. The numbers and titles of the pictures and tables are as follows: Figure S1. (A) Sequence length distribution at four tissues. (B) Sequence length distribution at five developmental stages. Figure S2. (A) Unigene annotations at four tissues. (B) Unigene annotations at five developmental stages. Figure S3. (A) Species classification statistics based on NR database alignment at four tissues. (B) Species classification statistics based on NR database alignment at five developmental stages. Figure S4. (A) Differential gene counts in four tissues grouped by differences. (B) Differential gene counts in five developmental stages grouped by differences. Figure S5. (A) Unigene misa statistical graph at four tissues. (B) Unigene misa statistical graph at five developmental stages. Table S1. Information on TPS subfamily genes used for phylogenetic analysis in Figure 3. Table S2. Primers of qRT-PCR used in Figure 5 and Figure 7. Additional File S2. The full-length amino acid sequence of NjTPS-s in this study.

## Glossary

TPS	terpene synthase
SMRT	single-molecule real-time
NGS	next-generation sequencing
HPLC	High-performance liquid chromatography
MEP	1-deoxy-D-xylulose-5-phosphate/methyl-erythritol-4-phosphate
MVA	mevalonate
IPP	isopentenyl diphosphate
DMAPP	dimethylallyl diphosphate
GPPS	geranyl diphosphate synthase
GPP	geranyl diphosphate
qRT-PCR	quantitative real-time polymerase chain reaction
ORF	open reading frames
FPKM	fragments per kilobase per million
ACCT	acetyl-coenzymeA (CoA)C-acetyltransferase
HMGS	3-hydroxy-3-methylglutaryl-CoA synthase
HMGR	3-hydroxy-3-methylglutaryl-CoA reductase
PMK	phosphomevalonate kinase
MDC	mevalonate-5-diphosphate
DXS	deoxyxyulose-5-phosphate
DXR	deoxyxyulose-5-phosphate
CMS	4-diphosphocytidyl-2-C-methyl-D-erythritol synthase
CMK	4-diphosphocytidyl-2-C-methyl-D-erythritol kinase
MCS	4-diphosphocytidyl-2-C-methyl-D-erythritol 2,4-cyclodiphosphate synthase
HDS	1-hydroxy-2-methyl-2(E)-butenyl 4-diphosphate synthase
HDR	1-hydroxy-2-methyl-2(E)-butenyl 4-diphosphate reductase
FPPS	farnesyl diphosphate synthase
FPP	farnesyl diphosphate
