# Supplementary material for: Identification of Key Genes Involved in Sesquiterpene Synthesis in Nardostachys jatamansi Based on Transcriptome and Component Analysis
Source: Genes (Basel). 2024 Nov 28;15(12):1539. doi: 10.3390/genes15121539 (PMC11675428; doi:10.3390/genes15121539)
Supplement: Supplementary file 1 [file genes-15-01539-s001.zip › Additional File S1.pdf]

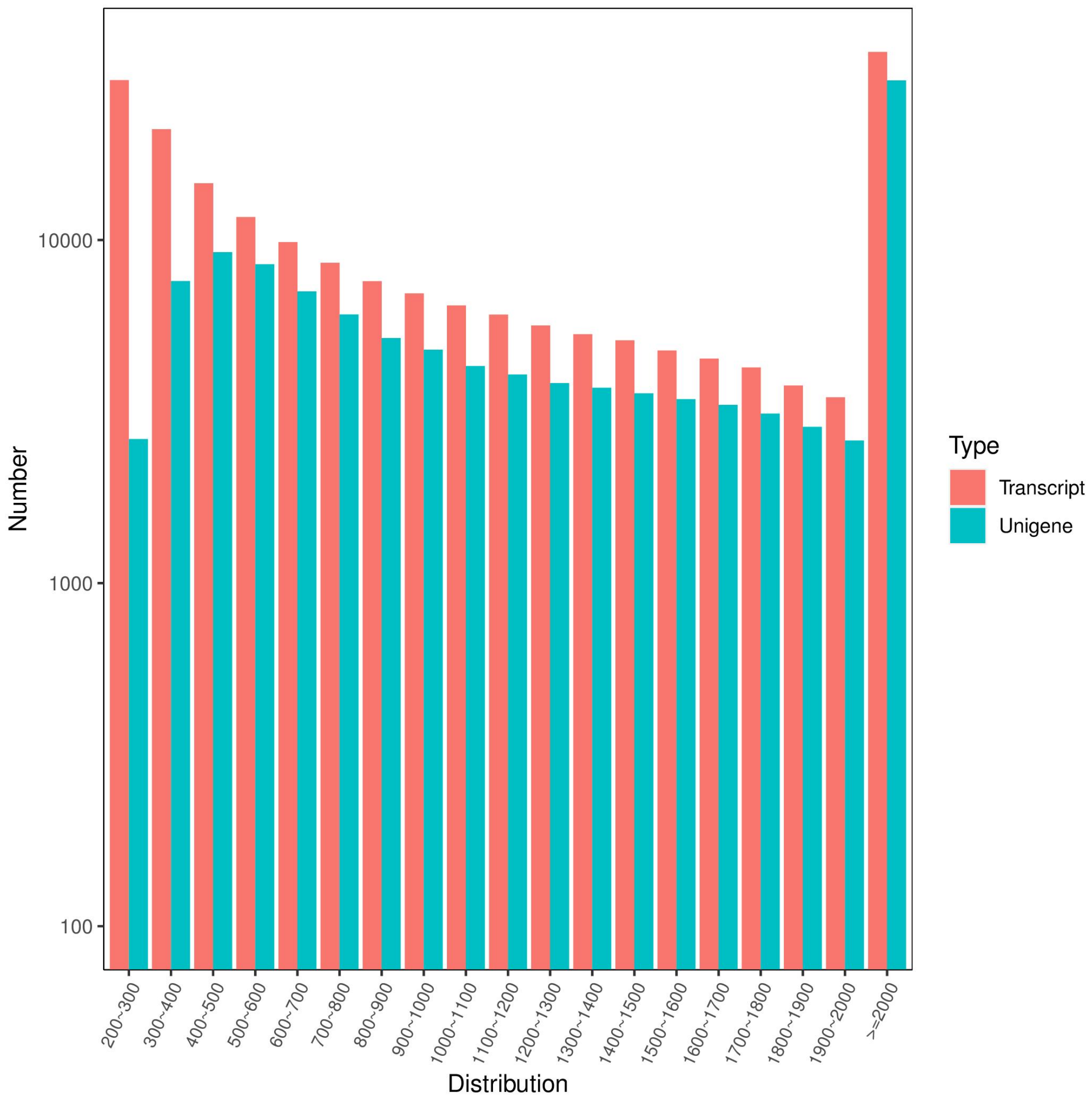

Figure S1A Sequence length distribution at four tissues

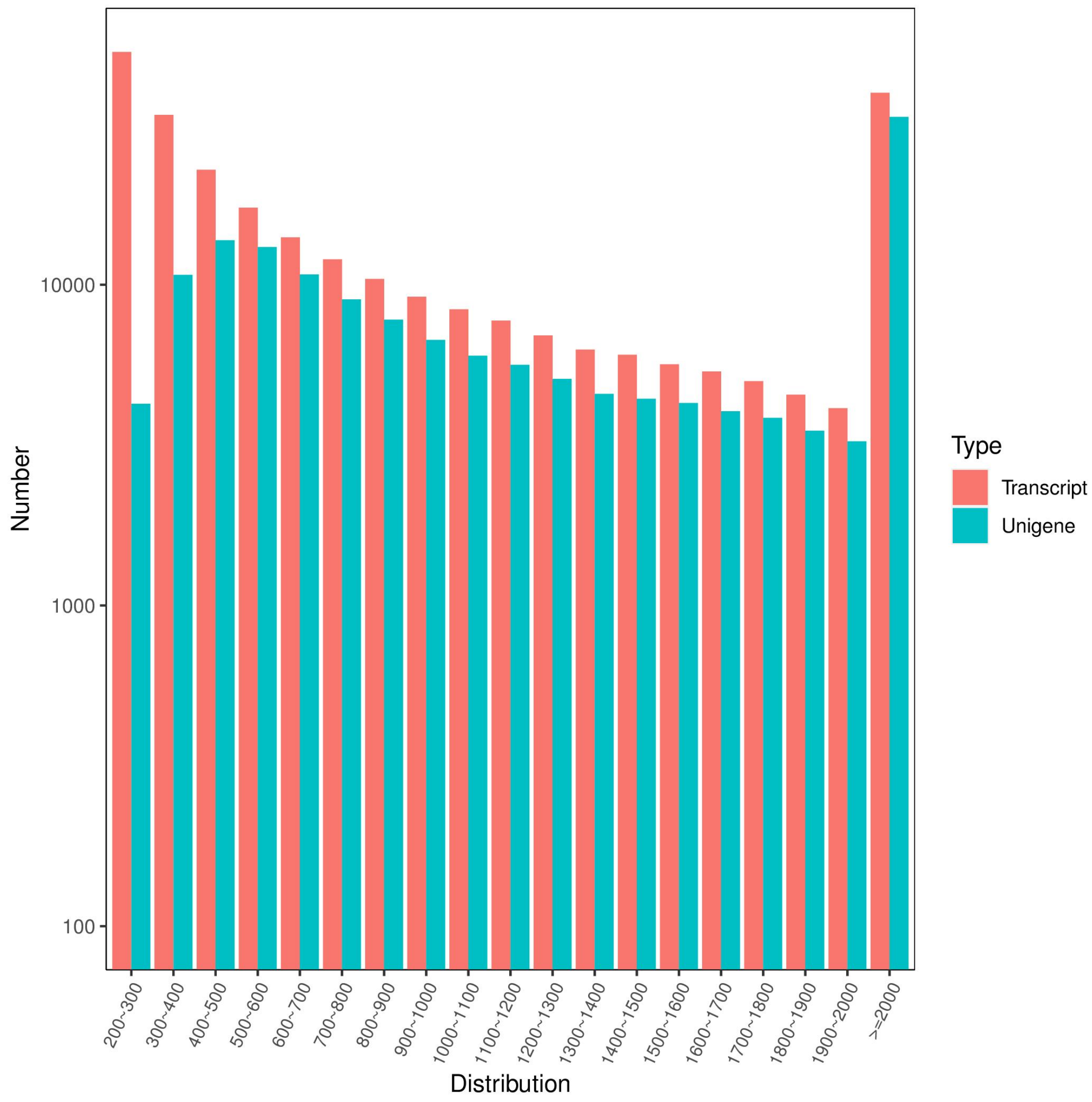

Figure S1B Sequence length distribution at five developmental stages

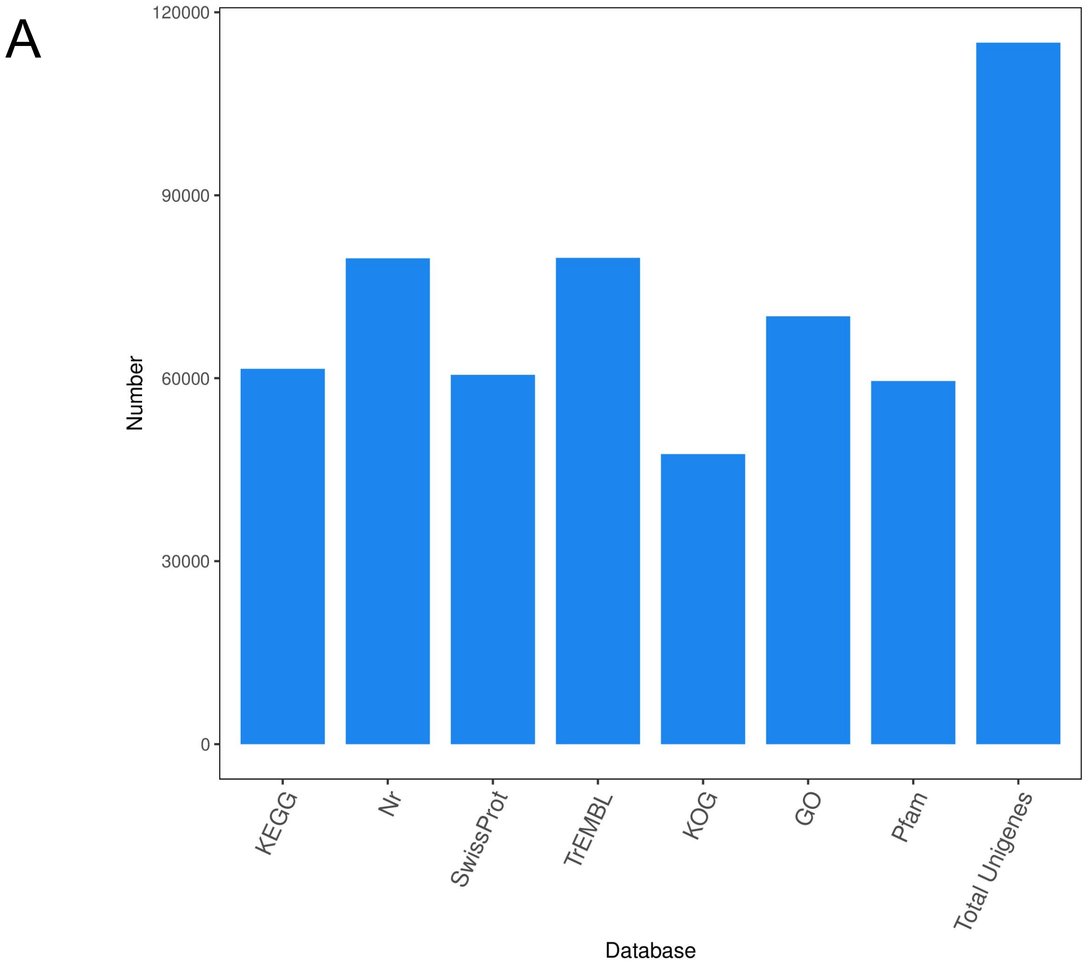

Figure S2A Unigene annotations at four tissues

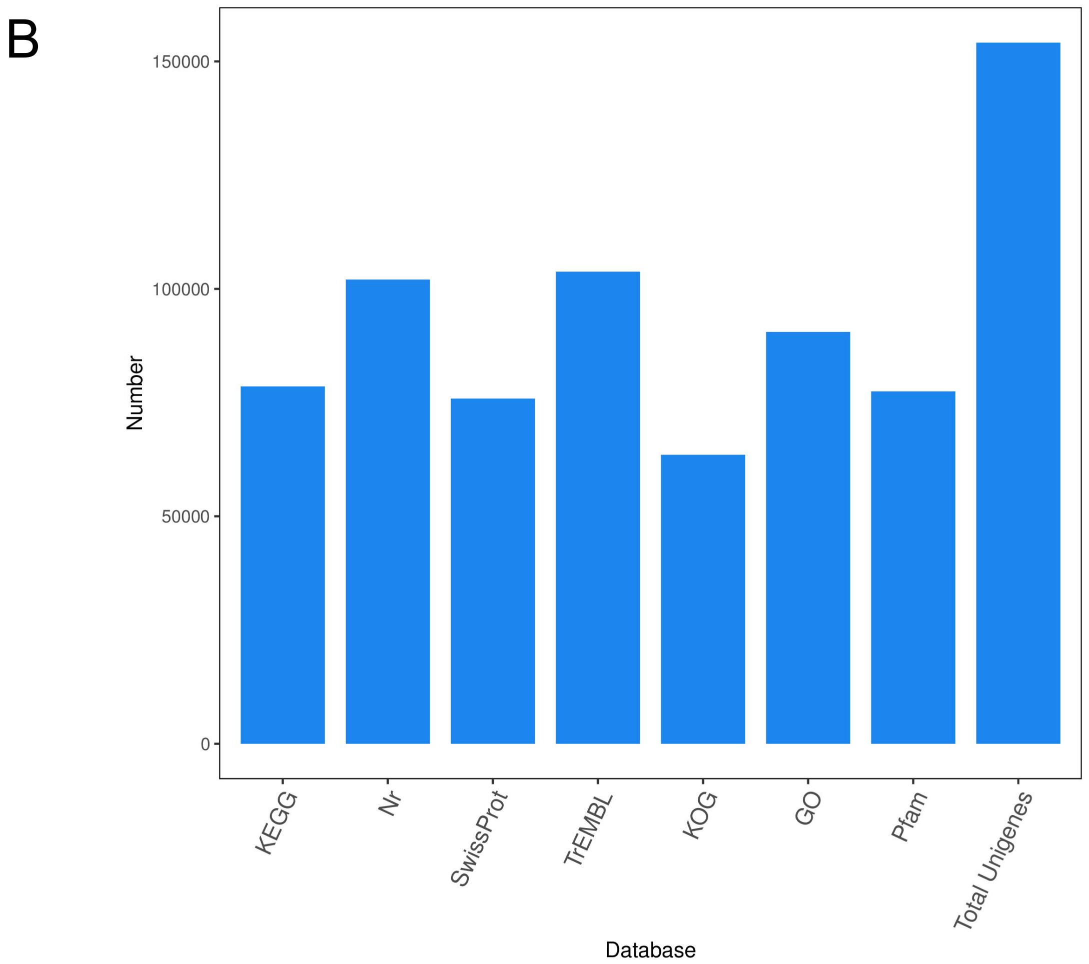

Figure S2B Unigene annotations at five developmental stages

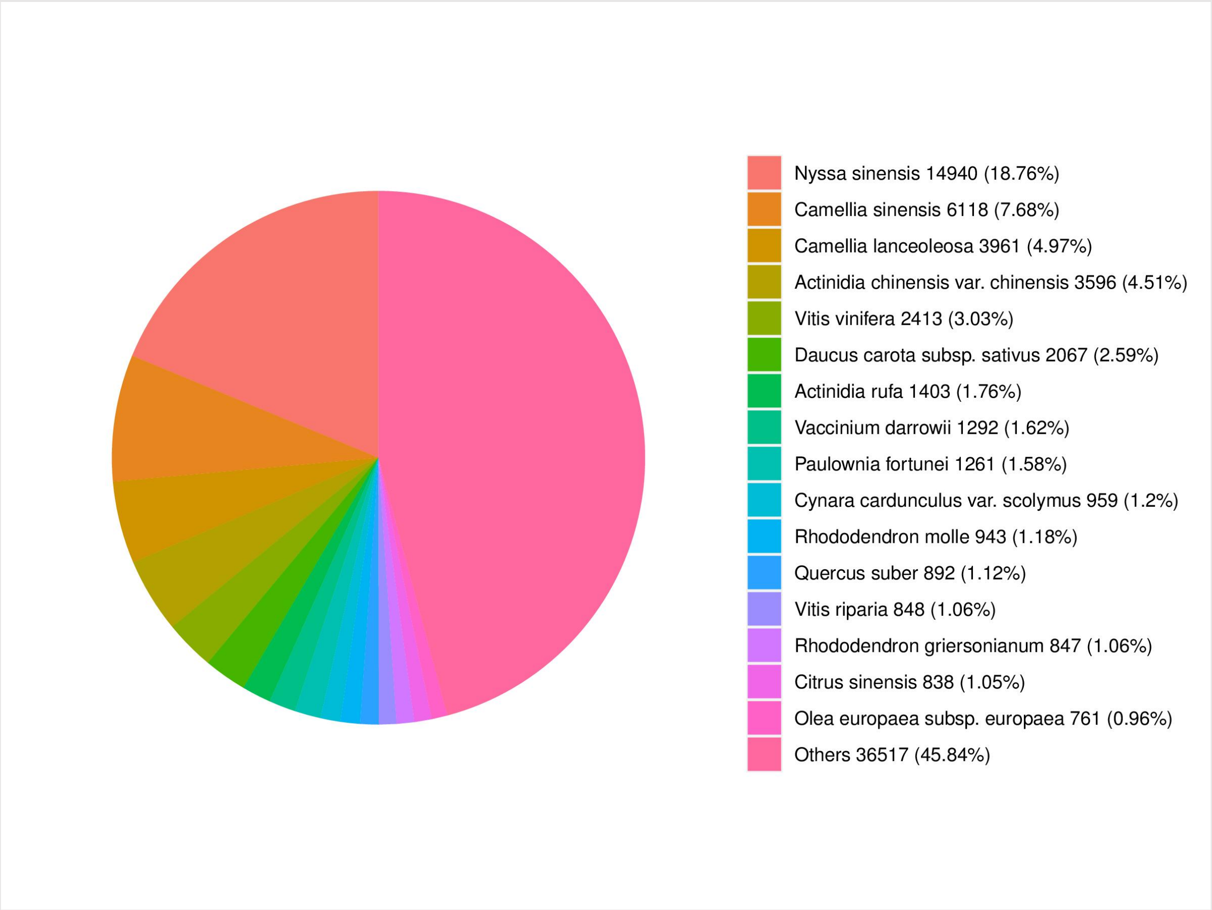

Figure S3A Species classification statistics based on NR database alignment at four tissues

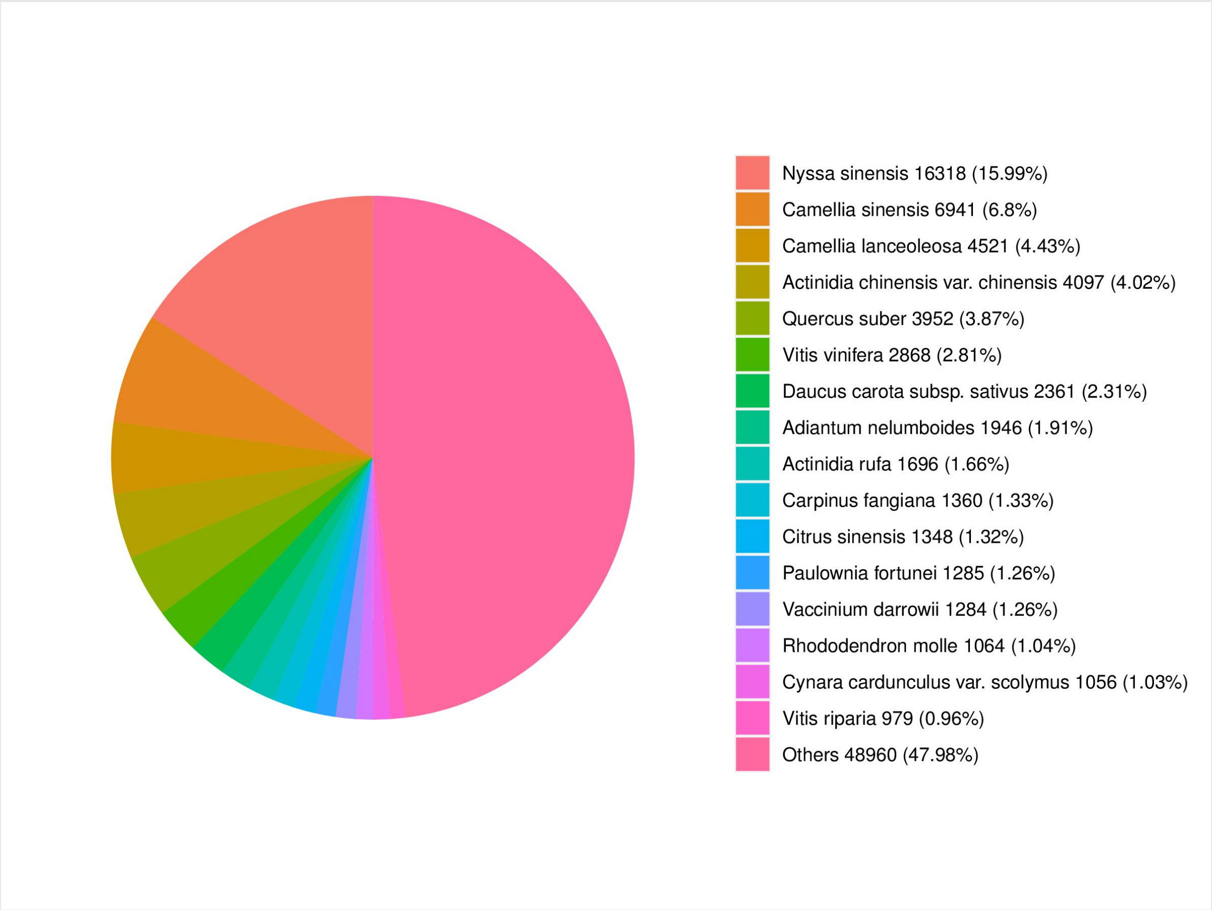

Figure S3B Species classification statistics based on NR database alignment at five developmental stages

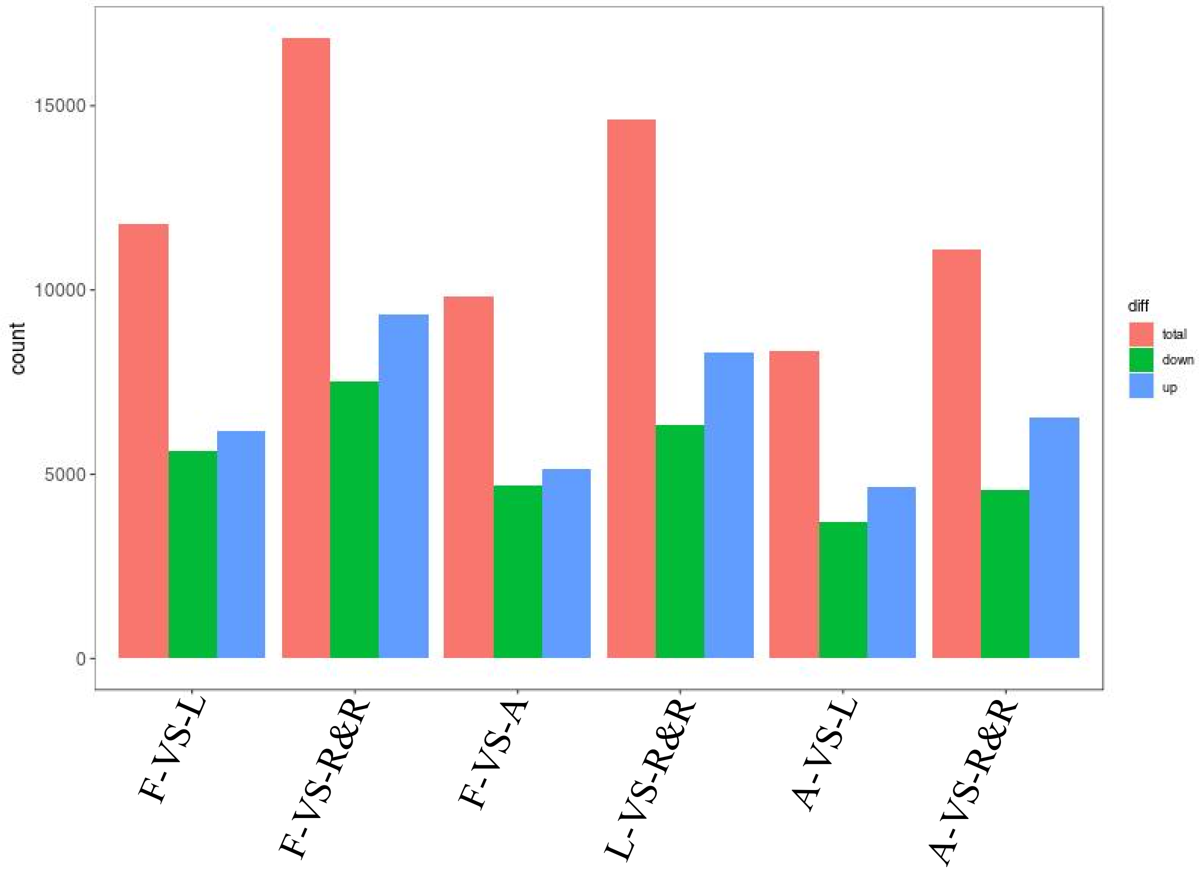

Figure S4A Differential gene counts in four tissues grouped by differences

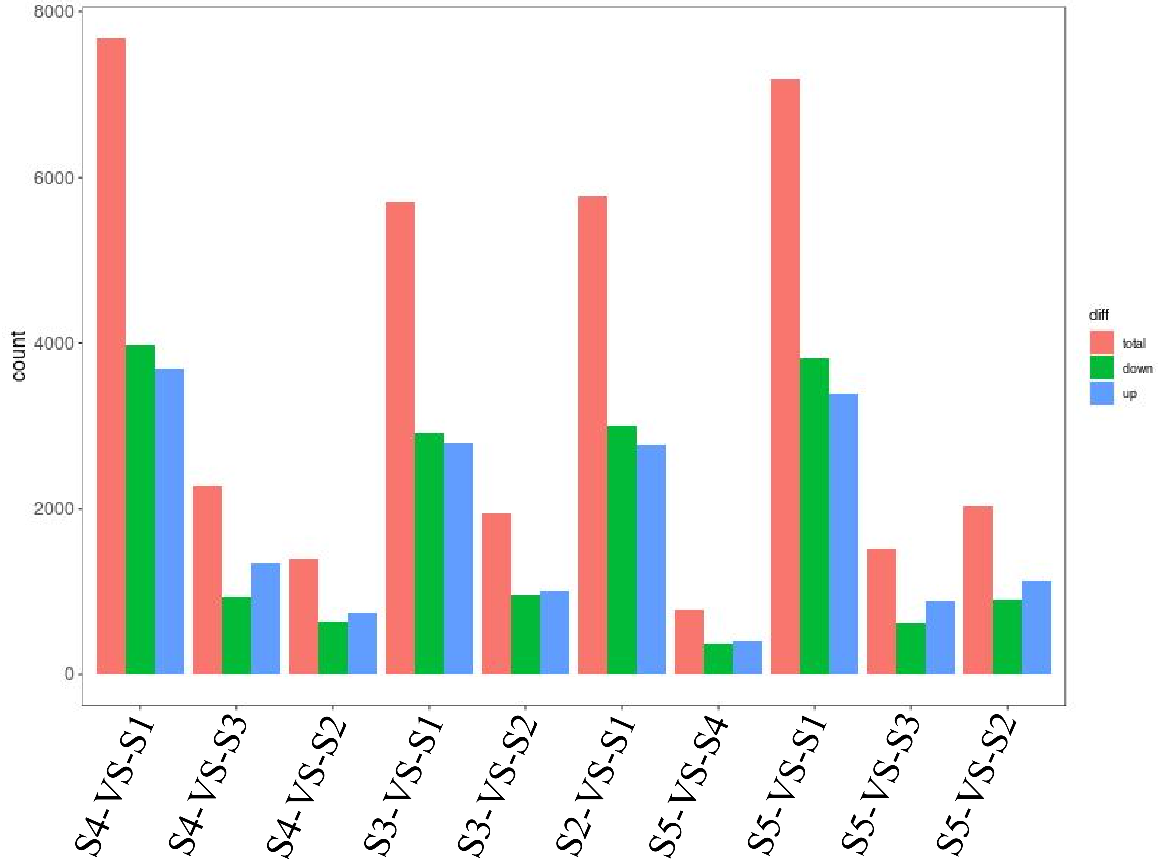

Figure S4B Differential gene counts in five developmental stages grouped by differences

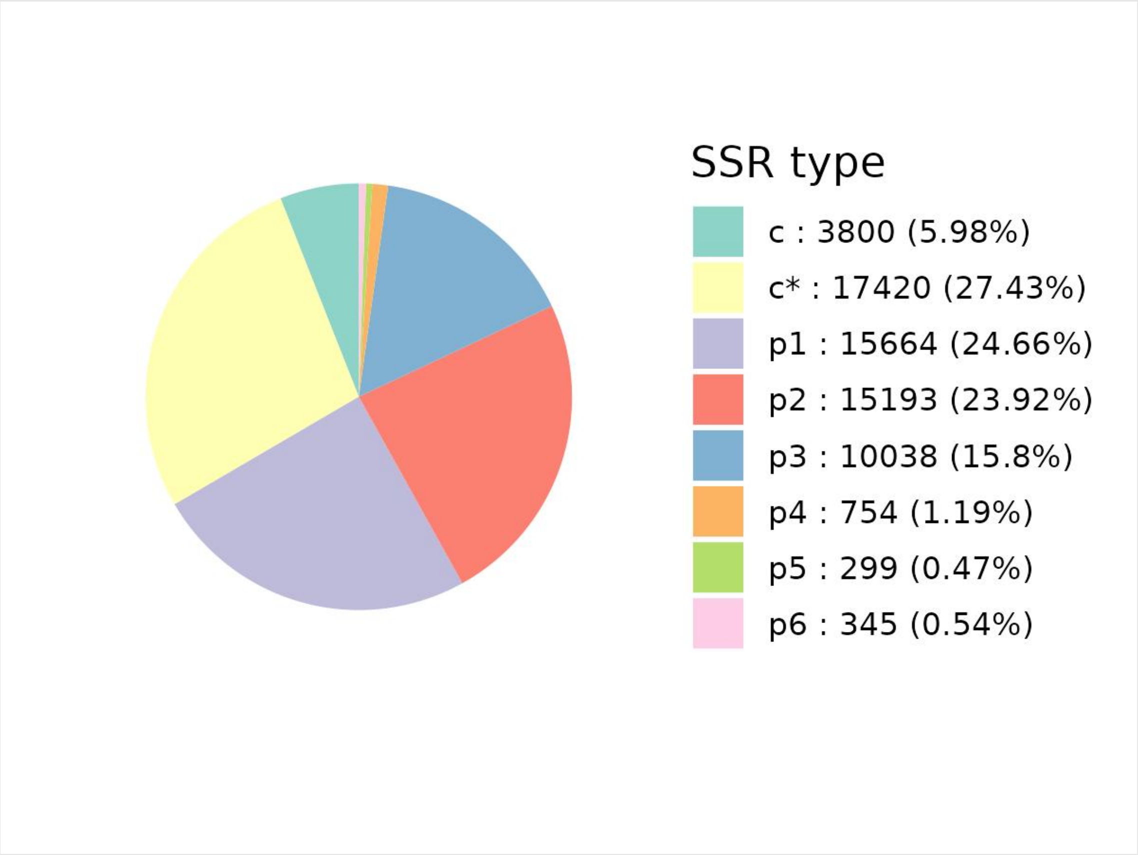

Figure S5A Unigene misa statistical graph at four tissues

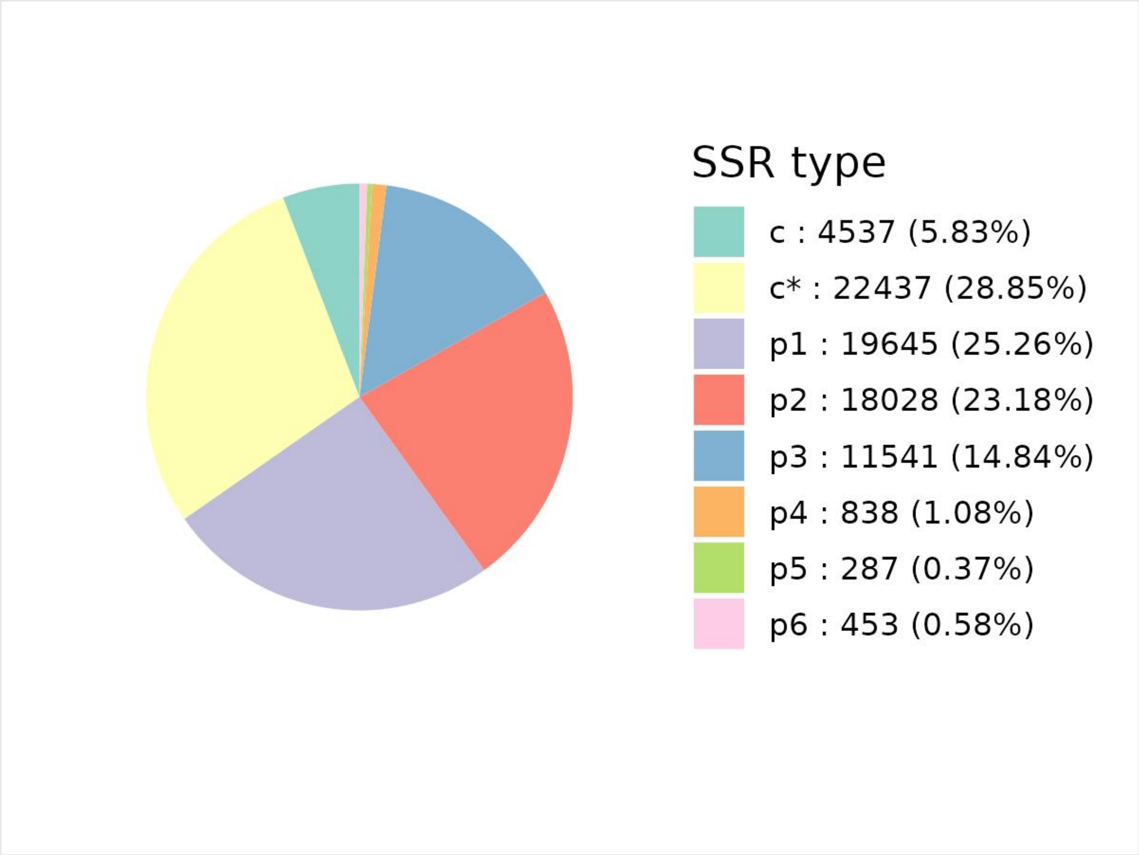

Figure S5B Unigene misa statistical graph at five developmental stages

Table S1 Information of TPS subfamily genes used for phylogenetic analysis in Fig. 3

Subfamily	Name	Accession No.	Species	Function/Product
TPS-b	FhLin	AFP23421.1	*Freesia hybrid cultivar*	linalool
TPS-b	SlTPS38	AEP82768.1	*Solanum lycopersicum*	α-bergamotene
TPS-b	VvaTerP	NP_001268216.1	*Vitis vinifera*	(-)-alpha-terpineol
TPS-b	PcAFS1	AAT70237.1	*PyruscommunisL.*	(E,E)-a-farnesene
TPS-g	AmMyr	AAO41727.1	*Antirrhinummajus*	myrcene
TPS-g	AtTPS14	NP_176361.2	*Arabidopsisthaliana*	(3S)-linalool
TPS-g	GmNES	AEE92791.1	*Glycinemax*	nerol
TPS-a	Cstps1	AAQ04608.1	*Citrus sinensis*	valencene
TPS-a	MdGDS-RG1	AGB14625.1	*Malus domestica*	germacrene-D
TPS-a	SlTPS12	AEP82783.1	Solanum lycopersicum	β-caryophyllene and α-humulene
TPS-c	OsCyc2	BAD42452.1	*Oryza sativa*	ent-copalyl diphosphate
TPS-c	SlTPS41	AEP82765.1	*Solanum lycopersicum*	Unknown
TPS-e/f	OsDTC2/KS8	XP_015617513.1	*Oryza sativa*	stemar-13-ene
TPS-e/f	LnTPS3	AKQ19359.1	*Laurus nobilis*	geranyllinalool
TPS-e/f	PdTPS	XP_017696375.1	*Phoenix dactylifera*	Unknown

Table S2 Primers of qRT-PCR used in Fig.5 and Fig.7

**Gene name**	**F-primer(5'to3')**	**R-primer(5'to3')**
NjActin	ATGGCGTGACTCATGTGGTT	ACAGTCTCAAAATCGGCGGT
NjTPS-29	GCCGAGATATCCAGGTGGTG	TCCTTCGGTTCTGGGAGGAT
NjTPS-32	GGGGTTACTACAGCTGGCTC	ACCCAAATCGTCCCACAGAC
NjTPS-33	GCCGAGATATCCAGGTGGTG	TCCTTCGGTTCTGGGAGGAT
NjTPS-34	AGCATGTGGTAAAGGCGTCA	AGCAGCGTTTCATCATGGGA
NjTPS-35	GGCATGGGAGTAAAGGCAGT	ACCCTATCGTCTGTGAGCCT
NjTPS-36	GGCATGGGAGTAAAGGCAGT	ACCCTATCGTCTGTGAGCCT
NjTPS-49	GCACTCCACCGCTCTATCTC	CCGTTTAGCTGCTCGGTACT
NjTPS-51	CCGTTTAGCTGCTCGGTACT	GCACTCCACCGCTCTATCTC
NjTPS-54	GGGGTTACTACAGCTGGCTC	ACCCAAATCGTCCCACAGTC
NjTPS-56	ACCCAAATCGTCCCACAGTC	GGGGTTACTACAGCTGGCTC
NjTPS-57	TGCTTTTTGTGGACCGTTGG	AAGGACGCTCCATCCGTTTT
NjTPS-59	ATCTGCGAATCGCGTTTGTG	ATCCTCCCAGAACCGAAGGA
