# Supplementary material for: Identification of Key Genes Involved in Sesquiterpene Synthesis in Nardostachys jatamansi Based on Transcriptome and Component Analysis
Source: Genes (Basel). 2024 Nov 28;15(12):1539. doi: 10.3390/genes15121539 (PMC11675428; doi:10.3390/genes15121539)
Supplement: Supplementary file 1 [file genes-15-01539-s001.zip › Additional File S2.pdf]

Additional File S2：Thefull-lengthaminoacidsequenceofNjTPSsinthisstudy

>NjTPS-1

MYENKEDMNPIVLQLAKLDYNMVQAVHLEDLKNVSRWWKSTSLGENLSFA
RDRLMENYIWTVEANFEPHLQYSRRMNTKVFALITTIDDVYDVYGTLDEL
KLFTDILERWDVNAIDQLPDYMKICFLVLFNTVNDMGYDALKNQNFNIIP
HLRNAWVDLCKSYMVEAKWYNNGYIPTLDEYLENAWISISAPTILLHAYA
FYSNPLTIDAFQCFEKNPNLVRWSSIILRLSNDLGTSNDELKRGDTLKSV
QCYMHETGVSEEKAREHIHYLVSETWKKMNEDRVIKDSPISNQVFVETAM
NIARMSQCMYQHGDGHGIENRETKDRVLSLLINPIHI

>NjTPS-2

MVRILDLKPRMDAVYHWINPSPLDKNTEELIEKIRERFEKVDLSISAYDT
AWVAMVTSALRHQEPCFPGCLEWILENQKADGSWGLNLSHPSLLKDSLSS
TLACVIALQKWNVGEKHIQRGLVFIGSKKYAAVDKYQSSPIGFDINFPAM
IKYANNLGLNLPLDSAFIDLMLHNRDTKLQRCKPRDLAYFAEGLVGESSY
DWEEIIKNGQGSNGSLFNSPAATAAVLINTRNDKCYDYLNSLLKINHGKG
VPTIYPFHLYTRLCMVDTIDRLGINRHFANELKVILDETYRCWLQKSEEI
YSDVSCCSMAFRLLRKNGYHVSSDALEEFIDEEHFFSTLSPQFRNTSTVV
QLYRASQMSFFQKEPVLDKINEWTTNFLRHQLLNHEIFDDDLLREVNYAF
EYPIDNMPRLTNRRGIELYNTDSFRMLKTSYRCCSVNNEEFLVLSQQEFN
KTQQIHLEEYKQVEEWLKKHRIGGLEFEWHMVTSSYFLAASGYIIPELSD
ARIIWAKVSVLGTIIDDLFDIDGTQEELLNLLHLIQNWDGNSNISSLDYS
SERTEIMFLALTEIINEQAAIGLIRQGRCIKKELIQVWQNFCKSCFKDVE
WWANKSTPTLDEYLANGCQTSGIGLWSTTFYFVGIQLSQDTLISEEYQII
YKHLGLIMRLFNDYQGVIDERDKVQRKMNGCLLLVSLSGGALTIEEARTE
VRKMIDISRKEVLRMMLTASSTTQKILMESFFCFHQQAYYLYTGNDKYRI
PSKKGLNDINRLLYEPLNLLPSYIREDKFA

>NjTPS-3

MVRILDLKPRMDAVYHWINPSPLDKNTEELIEKIRERFEKVDLSISAYDT
AWVAMVTSALRHQEPCFPGCLEWILENQKADGSWGLNLSHPSLLKDSLSS
TLACVIALQKWNVGEKHIQRGLVFIGSKKYAAVDKYQSSPIGFDINFPAM
IKYANNLGLNLPLDSAFIDLMLHNRDTKLQRCKPRDLAYFAEGLVGESSY
DWEEIIKNGQGSNGSLFNSPAATAAVLINTRNDKCYDYLNSLLKINHGKG
VPTIYPFHLYTRLCMVDTIDRLGINRHFANELKVILDETYRCWLQKSEEI
YSDVSCCSMAFRLLRKNRYHVSSDALEEFIDEEHFFSTLSPQFRNTSTVV

QLYRASQMSFFQKEPVLDKINEWTTNFLRHQLLNHEIYDDHLLREVKYAF
EYPIDNLPRLTNRRAIELYNIDSFRMLKTSYRCCSVNNEDFLVLSQQEFT
RTQQIHLEEYKQVEEYLYMHTSLNIHLRMNVK

>NjTPS-4

MSLTIFSSLKFVTSSTQLPSNGRIRQTQFIKCIPDDTRTCDQAIAMPRRL
ANYHPSIWEDDYVQSFGNNYTGESWSKRASELKEEVRGMFGKVEDSLMKV
ELIDTLQRLGISYQFEHEIKNTLENIYNEHYRSERWNNKEDYNLFAIALE
FRILRQHGYNVPQEIFNKFKDDHGNFKSSLCQDIKGMLYFYEASFLSIRG
ESILDDARDFTTKTLEEYLKNNKLVINNLDEILDLLVDHALELPLHWRML
RLEARWFIDVYERKQDVDTTLLEFAKIDFNMVQASHQEELKQMSRWWKST
YLGEKLRFARDRLMENYFWTTGVMFEPEYEYSRRMSTKINSIVTIIDDIY
DVYGSLQELQLFTNAVQRWDMKAMDELPDYMKICFLALYNSTNEMAYNTL
VQKGVYVISPLTKAV

>NjTPS-5

MDRSWMKKSRGDREYYLGVTEFLEFVKNNCQSPTGLYNCPCKRCNNMCDR
LTLEDIYNHLINFGIMQTYTVWNLHGEVSDIRSSYIRNTVDPEINLTMDF
VQDAFPFHELYDPPVLGDPAVPHVDPIAEEALTNYQQLLHEAQTPLYEGS
SETALGALLEAMSIKGSTNTSDATFTKWAKEHRRLLPAGNKYPESYAAAR
KSLKNIGMGYETIHACYYGCYLFYKEGALYEQCPVCGESRWEEDRTDGGK
RVPRKTVRYFPLTPRLQRLYMSPHTAKEMRWHGERERDDDFLRHPGDGEA
WKDFDRSYPQFAREFRNVRLALATDGFSPYGSTAVPYSLWPIVVIPYNFP
PSMCMKKEFNILTMLISGPKSPGKCLNVFMRPLIDELKVLWDTGVSTWDR
HGSESFMMRAAVMWTISDFPGLGMLGGIQTKGYKACPVCLDDIDATHSRS
RMSYMGARRWLPNDHRYRTDARAFNGKEERRAAPVTRSGSQVYEATLKHE
YPVLSLHPHKDFSKGKEKLCWTHPSIFWELPYWKKSRVRHALDVMHIEKN
VCDNVFGTVLGLDGKTKDDAKARKTLEDMGIRPHLWVKVGSSVKSTLPPA
IYTVPPKDRTDVFDQISAVKYPRGYAGSLKNKVNVSDKKFYGLKTHDCHV
LLQRVLPVVIRPYLGKYVADALVELSRFFQMLCARELKKSDVRQMQTDIV
MILCKLEMIFPPAFFTIMVHLCIHLPEQALLTGPVHYTCMPVVLFKCRW

>NjTPS-6

MAFRLLLRINGYQVSSDVLGEFVEQEHYLKTVSLQFTNTETIIQLYRASQI
PLYQNESILDKINAWTSTFLRHQLLNKQIYDERLRKEVEYAFGYPYINLD
RLRHRRGIELYPTDKFRMLKTSYRSSNIDNKDFLMFAQDDFNKCQAVQLE
EIQQLEKWAKNSRIANLDFDWHILTSSYIMKSGSLFAPEFKDSRIAWTQN
SFVGTIIDDVFDIDGSKEELLNLIELVERWDGHSNVSDVAYSSERIEILF
LAIQEIVNDQASRFLTRNGRCAKHEFLQMWRNFLGSMFREWEWWKTMSTP
TLDEFLTNGIITIGTGLFTINFYFLGVELSNYLFKSEEYRMLESHLGGIA

RLYNDYQSLERDLIQRKMNACYLIVSNSHGTKTLEDAKAQVRKMIEFRNR
EALRMLLQPDPDSVIPKQVVESFFNLNKLCYYLYHEADEFRAPGKTLDDI
RALLYDPINLL

>NjTPS-7

MFKSNKVELSASPYDTAWVAMVPSPNSSNAPCFPECLDWLLKNQLGNGSW
GLPSHSHLLLKDTLSSTLASVLALKRWNVGQSHINKGLHFMELNFQSAID
KNQHSPIGFDIIFPGMLNYAKDLDLKLPLEPTLLNAMLHTRDLELNRCYE
SKAEAYLAYVSEGMGKLQQDWEIVMKKYQRKNGSIFNSPATTAAVLTHHL
PDAASLNYIRLLLDKFGNAVPTVYPLDIYVRLCMIDNLERLGIDWHFRDE
IQTVLDETYRCWLQGDEQIFTDISTCAIAFRLLRMNGYDVSSDALTQIAE
EGNYLNSPGDRNLKGISDELELYKASQIIISPDESNLRKHNSKSSHILKY
ALSNDSFCSDKLATYIRQEVDDGLKFPFYASLERMENRRNIEQYSADCSV
IRVLKTSYCSPNIGNKDFLKLAIEDFNFIQSIHREEIKHVESWVIEGKLD
KLKFARQREAYCLFSAAATFFTPELSDARIIWAQNAVLTIVVDDFFDNAG
SPHEFLNLIRLAEK

>NjTPS-8

MMLASSHYGHKTTTASTSTVPSIEGTKERIRKMFKSNKVELSASPYDTAW
VAMVPSPNSSNAPCFPECLDWLLKNQLGNGSWGLPSHSHLLLKDTLSSTL
ASVLALKRWNVGQSHINKGLHFMELNFQSAIDKNQHSPIGFDIIFPGMLN
YAKDLDLKLPLEPTLLNAMLHTRDLELNRCYESKAEAYLAYVSEGMGKLQ
QDWEIVMKKYQRKNGSIFNSPATTAAVLTHHLPDAASLNYIRLLLDKFGN
AVPTVYPLDIYVRLCMIDNLERLGIDWHFRDEIQTVLDETYRCWLQGDEQ
IFTDISTCAIAFRLLRMNGYDVSSDALTQIAEEGNYLNSPGDRNLKGISD
ELELYKASQIIISPDESNLRKHNSKSSHILKYALSNDSFCSDKLATYIRQ
EVDDGLKFPFYASLERMENRRNIEQYSADCSVIRVLKTSYCSPNIGNKDF
LKLAIEDFNFIQSIHREEIKHVESWVIEGKLDKLKFARQREAYCLFSAAA
TFFTPELSDARIIWAQNAVLTIVVDDFFDNAGSPHEFLNLIRLAEKWNVD
IESDCCSKEVGILFFAIHNANTEIADKAFICQGRSVTNHIVQIWLDYLKA
LWIEAEWTRNKYVPSVDEYMENAYITVALGPIILPALYVVGEELSEEAVR
SIEFNKMFQLVSSCGRIINDTQTLERETEQGKLNIVSLLMIHGGITKEEA
IDEARNLIKRQREELLSLVVKKGSVVPRACKELFWKMAKVLHLFYDHDDG
LTSHHLMKAVNDIIYEPIP

>NjTPS-9

MMLASSHYGHKTTTASTSTVPYKKQSIEGTKERIRKMFKSNKVELSASPY
DTAWVAMVPSPNSSNAPCFPECLDWLLKNQLGNGSWGLPSHSHLLLKDTL
SSTLASVLALKRWNVGQSHINKGLHFMELNFQSAIDKNQHSPIGFDIIFP
GMLNYAKDLDLKLPLEPTLLNAMLHTRDLELNRCYESKAEAYLAYVSEGM

GKLQQDWEIVMKKYQRKNGSIFNSPATTAAVLTHHLPDAASLNYIRLLLD
KFGNAVPTVYPLDIYVRLCMIDNLERLGIDWHFRDEIQTVLDETYRCWLQ
GDEQIFTDISTCAIAFRLLRMNGYDVSSDALTQIAEEGNYLNSPGDRNLK
GISDELELYKASQIIISPDESNLRKHNSKSSHILKYALSNDSFCSDKLAT
YIRQEVDDGLKFPFYASLERMENRRNIEQYSADCSVIRVLKTSYCSPNIG
NKDFLKLAIEDFNFIQSIHREEIKHVESWVIEGKLDKLKFARQREAYCLF
SAAATFFTPELSDARIIWAQNAVLTIVVDDFFDNAGSPHEFLNLIRLAEK
WNVDIESDCCSKEVGILFFAIHNANTEIADKAFICQGRSVTNHIVQIWLD
YLKALWIEAEWTRNKYVPSVDEYMENAYITVALGPIILPALYVVGEELSE
EAVRSIEFNKMFQLVSSCGRIINDTQTLEVIIHTHT

>NjTPS-10

MSSFLQTASSHYGHKTTTASTSTVPYKKQSIEGTKERIRKMFKSNKVELS
ASPYDTAWVAMVPSPNSSNAPCFPECLDWLLKNQLGNGSWGLPSHSHLLL
KDTLSSTLASVLALKRWNVGQSHINKGLHFMELNFQSAIDKNQHSPIGFD
IIFPGMLNYAKDLDLKLPLEPTLLNAMLHTRDLELNRCYESKAEAYLAYV
SEGMGKLQQDWEIVMKKYQRKNGSIFNSPATTAAVLTHHLPDAASLNYIR
LLLDKFGNAVPTVYPLDIYVRLCMIDNLERLGIDWHFRDEIQTVLDETYR
CWLQGDEQIFTDISTCAIAFRLLRMNGYDVSSDALTQIAEEGNYLNSPGD
RNLKGISDELELYKASQIIISPDESSALRKQNLQSSNFLKQMLSDDSYCS
DKLSRSISQEVDDALNFPFCASLERMANRRYIEQYNVDTSTIRVLKTSYF
SSNIGNKDFLKLAVEDFNKCQSRHREDAAYLARWVIENRLDKLKFVRQKS
FGYMSFSAAATSFTPKLSDARMSWAKNALLTTVVDDFFDIGGSMDELLNL
IYLVDKWDNVDIESDCCSEHVGIIFSALQRGINEIAELAFVYQERNVTSH
IVEIWLDLLKSMLREAEWSRDRYVPSMEEYMENGYVSFALGPILLPALYL
VGPKLSDESARSFQLKKLFRLMSNCGRLLNDIQGFKRESKEGKLNSVSLR
MMMMMNEGDTDEGIIVNELKMLVESYKEELLRIVIEEKESVLPRECKELF
WKMTKVVHQFYLKDDGFTSQHMMKAVNDVIYQPIILEEHQLK

>NjTPS-11

MYLSQSFTLTKTYYPYRITASFHSHSTAKTATNATTTTTVPSIEDGSKER
IRKLFKKVEVSVSSYDTAWVAMIPGRDGYGPIFPECLDWLLNNQLHDGSW
GLNPPFLLKDTLSSTLASILALKRWGVGQHHINKGMKFMDLNFHSAIDKN
HHSPIGFDIIFPGMLDYAKDLDLKLPLKPALSDAIFLNRESELKRCYESE
AHERDAYLAYVSEGIGKSQDWKMVMKHQSKNGSLFNSPATTAAALTHHLP
SDSGLLNYIRLLLHKFGNAVPTSYPVDIFVRLCMIDNLERLGIDWHFRDE
IQSVLDETYRCWIKGDEEIFMDVSTCAIAFRLLRMNGYDVSSDLLIRTAE
ENDCFSSSGGHLKGISDTLELYRASQIIISPDEFNLRKHNSKSSHILKYA
LSNDSFCSDKLATYIRQEVDDGLKFPFYASLERMENRRNIEQYSADCSVI
RVLKTSYCSPNIGNKDFLKLAIEDFNFIQSIHREEIKHVESWVIEGKLDK
LKFARQREAYCLFSAAATFFTPELSDARIIWAQNAVLTIVVDDFFDNAGS
PHEFLNL

>NjTPS-12

MMLASSHYGHKTTTASTSTVPYKKQSIEGTKERIRKMFKSNKVELSASPY
DTAWVAMVPSPNSSNAPCFPECLDWLLKNQLGNGSWGLPSHSHLLLKDTL
SSTLASVLALKRWNVGQSHINKGLHFMELNFQSAIDKNQHSPIGFDIIFP
GMLNYAKDLDLKLPLEPTLLNAMLHTRDLELNRCYESKAEAYLAYVSEGM
GKLQQDWEIVMKKYQRKNGSIFNSPATTAAVLTHHLPDAASLNYIRLLLD
KFGNAVPTVYPLDIYVRLCMIDNLERLGIDWHFRDEIQTVLDETYRCWLQ
GDEQIFTDISTCAIAFRLLRMNGYDVSSDALTQIAEEGNYLNSPGDRNLK
GISDELELYKASQIIISPDESNLRKHNSKSSHILKYALSNDSFCSDKLAT
YIRQEVDDGLKFPFYASLERMENRRNIEQYSADCSVIRVLKTSYCSPNIG
NKDFLKLAIEDFNFIQSIHREEIKHVESWVIEGKLDKLKFARQREAYCLF
SAAATFFTPELSDARIIWAQNAVLTIVVDDFFDNAGSPHEFLNLIRLAEK

>NjTPS-13

MMLASSHYGHKTTTASTSTVPYKKQSIEGTKERIRKMFKSNKVELSASPY
DTAWVAMVPSPNSSNAPCFPECLDWLLKNQLGNGSWGLPSHSHLLLKDTL
SSTLASVLALKRWNVGQSHINKGLHFMELNFQSAIDKNQHSPIGFDIIFP
GMLNYAKDLDLKLPLEPTLLNAMLHTRDLELNRCYESKAEAYLAYVSEGM
GKLQQDWEIVMKKYQRKNGSIFNSPATTAAVLTHHLPDAASLNYIRLLLD
KFGNAVPTVYPLDIYVRLCMIDNLERLGIDWHFRDEIQTVLDETYRCWLQ
GDEQIFTDISTCAIAFRLLRMNGYDVSSDALTQIAEEGNYLNSPGDRNLK
GISDELELYKASQIIISPDESSALRKQNLQSSNFLKQMLSDDSYCSDKLS
RSISQEVDDALNFPFCASLERMANRRYIEQYNVDTSTIRVLKTSYFSSNI
GNKDFLKLAVEDFNKCQSRHREDAAYLARWVIENRLDKLKFVRQKSFGYM
SFSAAATSFTPKLSDARMSWAKNALLTTVVDDFFDIGGSMDELLNLIYLV
DKWDNVDIESDCCSEHVGIIFSALQRGINEIAELAFVYQERNVTSHIVEI
WLDLLKSMLREAEWSRDRYVPSMEEYMENGYVSFALGPILLPALYLVGPK
LSDESARSFQLKKLFRLMSNCGRLLNDIQGFKRESKEGKLNSVSLRMMMM
MNEGDTDEGIIVNELKMLVESYKEELLRIVIEEKESVLPRECKELFWKMT
KVVHQFYLKDDGFTSQHMMKAVNDVIYQPIILEQ

>NjTPS-14

MMLASSHYGHKTTTASTSTVPYKKQSIEGTKERIRKMFKSNKVELSASPY
DTAWVAMVPSPNSSNAPCFPECLDWLLKNQLGNGSWGLPSHSHLLLKDTL
SSTLASVLALKRWNVGQSHINKGLHFMELNFQSAIDKNQHSPIGFDIIFP
GMLNYAKDLDLKLPLEPTLLNAMLHTRDLELNRCYESKAEAYLAYVSEGM
GKLQQDWEIVMKKYQRKNGSIFNSPATTAAVLTHHLPDAASLNYIRLLLD
KFGNAVPTVYPLDIYVRLCMIDNLERLGIDWHFRDEIQTVLDETYRCWLQ
GDEQIFTDISTCAIAFRLLRMNGYDVSSDALTQIAEEGNYLNSPGDRNLK
GISDELELYKASQIIISPDESNLRKHNSKSSHILKYALSNDSFCSDKLAT

YIRQEVDDGLKFPFYASLERMENRRNIEQYSADCSVIRVLKTSYCSPNIG
NKDFLKLAIEDFNFIQSIHREEIKHVESWVIEGKLDKLKFARQREAYCLF
SAAATFFTPELSDARIIWAQNAVLTIVVDDFFDNAGSPHEFLNLIRLAEK

>NjTPS-15

MMLASSHYGHKTTTASTSTVPYKKQSIEGTKERIRKMFKSNKVELSASPY
DTAWVAMVPSPNSSNAPCFPECLDWLLKNQLGNGSWGLPSHSHLLLKDTL
SSTLASVLALKRWNVGQSHINKGLHFMELNFQSAIDKNQHSPIGFDIIFP
GMLNYAKDLDLKLPLEPTLLNAMLHTRDLELNRCYESKAEAYLAYVSEGM
GKLQQDWEIVMKKYQRKNGSIFNSPATTAAVLTHHLPDAASLNYIRLLLD
KFGNAVPTVYPLDIYVRLCMIDNLERLGIDWHFRDEIQTVLDETYRCWLQ
GDEQIFTDISTCAIAFRLLRMNGYDVSSDALTQIAEEGNYLNSPGDRNLK
GISDELELYKASQIIISPDESNLRKHNSKSSHILKYALSNDSFCSDKLAT
YIRQEVDDGLKFPFYASLERMENRRNIEQYSADCSVIRVLKTSYCSPNIG
NKDFLKLAIEDFNFIQSIHREEIKHVESWVIEGKLDKLKFARQREAYCLF
SAAATFFTPELSDARIIWAQNAVLTIVVDDFFDNAGSPHEFLNLIRLAEK
WNVDIESDCCSKEVGILFFAIHNANTEIADKAFICQGRSVTNHIVQIWLD
YLKALWIEAEWTRNKYVPSVDEYMENAYITVALGPIILPALYVVGEELSE
EAVRSIEFNKMFQLVSSCGRIINDTQTLERETEQGKLNIVSLLMIHGGIT
KEEAIDEARNLIKRQREELLSLVVKKGSVVPRACKELFWKMAKVLHLFYD
HDDGLTSHHLMKAVNDIIYEPIP

>NjTPS-16

MMLASSHYGHKTTTASTSTVPYKKQSIEGTKERIRKMFKSNKVELSASPY
DTAWVAMVPSPNSSNAPCFPECLDWLLKNQLGNGSWGLPSHSHLLLKDTL
SSTLASVLALKRWNVGQSHINKGLHFMELNFQSAIDKNQHSPIGFDIIFP
GMLNYAKDLDLKLPLEPTLLNAMLHTRDLELNRCYESKAEAYLAYVSEGM
GKLQQDWEIVMKKYQRKNGSIFNSPATTAAVLTHHLPDAASLNYIRLLLD
KFGNAVPTVYPLDIYVRLCMIDNLERLGIDWHFRDEIQTVLDETYRCWLQ
GDEQIFTDISTCAIAFRLLRMNGYDVSSDALTQIAEEGNYLNSPGDRNLK
GISDELELYKASQIIISPDESSALRKQNLQSSNFLKQMLSDDSYCSDKLS
RSISQEVDDALNFPFCASLERMANRRYIEQYNVDTSTIRVLKTSYFSSNI
GNKDFLKLAVEDFNKCQSRHREDAAYLARWVIENRLDKLKFVRQKSFGYM
SFSAAATSFTPKLSDARMSWAKNALLTTVVDDFFDIGGSMDELLNLIYLV
DKWDNVDIESDCCSEHVGIIFSALQRGINEIAELAFVYQERNVTSHIVEI
WLDLLKSMLREAEWSRDRYVPSMEEYMENGYVSFALGPILLPALYLVGPK
LSDESARSFQLKKLFRLMSNCGRLLNDIQGFKRESKEGKLNSVSLRMMMM
MNEGDTDEGIIVNELKMLVESYKEELLRIVIEEKESVLPRECKELFWKMT
KVVHQFYLKDDGFTSQHMMKAVNDVIYQPIILEEHQLK

>NjTPS-17

MSSFLQTASSHYGHKTTTASTSTVPSIEGTKERIRKMFKSNKVELSASPY
DTAWVAMVPSPNSSNAPCFPECLDWLLKNQLGNGSWGLPSHSHLLLKDTL
SSTLASVLALKRWNVGQSHINKGLHFMELNFQSAIDKNQHSPIGFDIIFP
GMLNYAKDLDLKLPLEPTLLNAMLHTRDLELNRCYESKAEAYLAYVSEGM
GKLQQDWEIVMKKYQRKNGSIFNSPATTAAVLTHHLPDAASLNYIRLLLD
KFGNAVPTVYPLDIYVRLCMIDNLERLGIDWHFRDEIQTVLDETYRCWLQ
GDEQIFTDISTCAIAFRLLRMNGYDVSSDALTQIAEEGNYLNSPGDRNLK
GISDELELYKASQIIISPDESSALRKQNLQSSNFLKQMLSDDSYCSDKLS
RSISQEVDDALNFPFCASLERMANRRYIEQYNVDTSTIRVLKTSYFSSNI
GNKDFLKLAVEDFNKCQSRHREDAAYLARWVIENRLDKLKFVRQKSFGYM
SFSAAATSFTPKLSDARMSWAKNALLTTVVDDFFDIGGSMDELLNLIYLV
DKWDNVDIESDCCSEHVGIIFSALQRGINEIAELAFVYQERNVTSHIVEI
WLDLLKSMLREAEWSRDRYVPSMEEYMENGYVSFALGPILLPALYLVGPK
LSDESARSFQLKKLFRLMSNCGRLLNDIQGFKRESKEGKLNSVSLRMMMM
MNEGDTDEGIIVNELKMLVESYKEELLRIVIEEKESVLPRECKELFWKMT
KVVHQFYLKDDGFTSQHMMKAVNDVIYQPIILEEHQLK

>NjTPS-18

MFKSNKVELSASPYDTAWVAMVPSPNSSNAPCFPECLDWLLKNQLGNGSW
GLPSHSHLLLKDTLSSTLASVLALKRWNVGQSHINKGLHFMELNFQSAID
KNQHSPIGFDIIFPGMLNYAKDLDLKLPLEPTLLNAMLHTRDLELNRCYE
SKAEAYLAYVSEGMGKLQQDWEIVMKKYQRKNGSIFNSPATTAAVLTHHL
PDAASLNYIRLLLDKFGNAVPTVYPLDIYVRLCMIDNLERLGIDWHFRDE
IQTVLDETYRCWLQGDEQIFTDISTCAIAFRLLRMNGYDVSSDALTQIAE
EGNYLNSPGDRNLKGISDELELYKASQIIISPDESNLRKHNSKSSHILKY
ALSNDSFCSDKLATYIRQEVDDGLKFPFYASLERMENRRNIEQYSADCSV
IRVLKTSYCSPNIGNKDFLKLAIEDFNFIQSIHREEIKHVESWVIEGKLD
KLKFARQREAYCLFSAAATFFTPELSDARIIWAQNAVLTIVVDDFFDNAG
SPHEFLNLIRLAEKWNVDIESDCCSKEVGILFFAIHNANTEIADKAFICQ
GRSVTNHIVQIWLDYLKALWIEAEWTRNKYVPSVDEYMENAYITVALGPI
ILPALYVVGEELSEEAVRSIEFNKMFQLVSSCGRIINDTQTLERETEQGK
LNIVSLLMIHGGITKEEAIDEARNLIKRQREELLSLVVKKGSVVPRACKE
LFWKMAKVLHLFYDHDDGLTSHHLMKAVNDIIYEPIP

>NjTPS-19

MFKSNKVELSASPYDTAWVAMVPSPNSSNAPCFPECLDWLLKNQLGNGSW
GLPSHSHLLLKDTLSSTLASVLALKRWNVGQSHINKGLHFMELNFQSAID
KNQHSPIGFDIIFPGMLNYAKDLDLKLPLEPTLLNAMLHTRDLELNRCYE
SKAEAYLAYVSEGMGKLQQDWEIVMKKYQRKNGSIFNSPATTAAVLTHHL

PDAASLNYIRLLLDKFGNAVPTVYPLDIYVRLCMIDNLERLGIDWHFRDE
IQTVLDETYRCWLQGDEQIFTDISTCAIAFRLLRMNGYDVSSDALTQIAE
EGNYLNSPGDRNLKGISDELELYKASQIIISPDESSALRKQNLQSSNFLK
QMLSDDSYCSDKLSRSISQEVDDALNFPFCASLERMANRRYIEQYNVDTS
TIRVLKTSYFSSNIGNKDFLKLAVEDFNKCQSRHREDAAYLARWVIENRL
DKLKFVRQKSFGYMSFSAAATSFTPKLSDARMSWAKNALLTTVVDDFFDI
GGSMDELLNLIYLVDKWDNVDIESDCCSEHVGIIFSALQRGINEIAELAF
VYQERNVTSHIVEIWLDLLKSMLREAEWSRDRYVPSMEEYMENGYVSFAL
GPILLPALYLVGPKLSDESARSFQLKKLFRLMSNCGRLLNDIQGFKRESK
EGKLNSVSLRMMMMMNEGDTDEGIIVNELKMLVESYKEELLRIVIEEKES
VLPRECKELFWKMTKVVHQFYLKDDGFTSQHMMKAVNDVIYQPIILEEHQ
LK

>NjTPS-20

MLESMDDGEISVSAYDTAWVALVEDINGSGAPQFPSSLDWIADNQLPDGS
WGDRYIFSAFERIINTLACIIALRTWNIHHQKSEKGMLFIKENIGKLEEE
NEEHMPIGFEVAFPSLVEIAKNLGIQIFPEESAVLQEIYARRNLKLTRIP
KDIMHKIPTTLLHSLEGMKELEWEKLIKLQCKDGSFLFSPSSTAFALIHT
HDSNALSYLTNTVHKFNGGGIFLFHYWKLLFFLLFLLIMWFVHTTLCLFS
VPNVYPVDLFEHIWAVDRLQRLGISRHFKSQIKDCLDYVHKYWREEGICW
ARNSPVQDIDDTAMGFRLLRLHGYPVSADVFENFKNGEEFFCFNGQSNQA
VTGMYNLLRASQIVLPGEKILEDAKKFSYQFLKHKRAANQILDKWIITKD
LPGEVNYALDMPWYASLSRVETRYYLEQYGGQDDVWIGKTLYRMSKVNNN
TYMELAKLDYTNCQAMHLEEWSRIKQWYEECKLGEYGASQESLVLLAYYL
ASASIYEPEKSKERIAWAKTTTLMETIMSYFGTKNMSIEQRRSFVRDFKK
CSNNLHFTYGRYKTGQGLMGVLLDTVYQFSLEALRTHGRDIHRHLTQAWE
TWMLTWQEEGDMYRGQAELVVRTINLCAGSCSSEEQLLNMMSHPQYHRLS
QLTNKICHRLRLFQSHKEYEGESINTENGGINEEEIEADMRELVEAVIYN
EDDDVDGNIKQTFLTVAKTFYYTAYCNPHTISLHISKVLFESLF

>NjTPS-21

MLESMDDGEISVSAYDTAWVALVEDINGSGAPQFPSSLDWIADNQLPDGS
WGDRYIFSAFERIINTLACIIALRTWNIHHQKSEKGMLFIKENIGKLEEE
NEEHMPIGFEVAFPSLVEIAKNLGIQIFPEESAVLQEIYARRNLKLTRIP
KDIMHKIPTTLLHSLEGMKELEWEKLIKLQCKDGSFLFSPSSTAFALIHT
HDSNALSYLTNTVHKFNGGVPNVYPVDLFEHIWAVDRLQRLGISRHFKSQ
IKDCLDYVHKYWREEGICWARNSPVQDIDDTAMGFRLLRLHGYPVSADVF
ENFKNGEEFFCFNGQSNQAVTGMYNLLRASQIVLPGEKILEDAKKFSYQF
LKHKRAANQILDKWIITKDLPGEVNYALDMPWYASLSRVETRYYLEQYGG
QDDVWIGKTLYRMSKVNNNTYMELAKLDYTNCQAMHLEEWSRIKQWYEEC
KLGEYGASQESLVLLAYYLASASIYEPEKSKERIAWAKTTTLMETIMSYF
GTKNMSIEQRRSFVRDFKKCSNNLHFTYGRYKTGQGLMGVLLDTVYQFSL

EALRTHGRDIHRHLTQAWETWMLTWQEEGDMYRGQAELVVRTINLCAGSC
SSEEQLLNMMSHPQYHRLSQLTNKICHRLRLFQSHKEYEGESINTENGGI
NEEEIEADMRELVEAVIYNEDDDVDGNIKQTFLTVAKTFYYTAYCNPHTI
SLHISKVLFESLF

>NjTPS-22

MLESMDDGEISVSAYDTAWVALVEDINGSGAPQFPSSLDWIADNQLPDGS
WGDRYIFSAFERIINTLACIIALRTWNIHHQKSEKGMLFIKENIGKLEEE
NEEHMPIGFEVAFPSLVEIAKNLGIQIFPEESAVLQEIYARRNLKLTRIP
KDIMHKIPTTLLHSLEGMKELEWEKLIKLQCKDGSFLFSPSSTAFALIHT
HDSNALSYLTNTVHKFNGGVPNVYPVDLFEHIWAVDRLQRLGISRHFKSQ
IKDCLDYVHKYWREEGICWARNSPVQDIDDTAMGFRLLRLHGYPVSADVF
ENFKNGEEFFCFNGQSNQAVTGMYNLLRASQIVLPGEKILEDAKKFSYQF
LKHKRAANQILDKWIITKDLPGEVNYALDMPWYASLSRVETRYYLEQYGG
QDDVWIGKTLYRMSKVNNNTYMELAKLDYTNCQAMHLEEWSRIKQWYEEC
KLGEYGASQESLVLLAYYLASASIYEPEKSKERIAWAKTTTLMETIMSYF
GTKNMSIEQRRSFVRDFKKCSNNLHFTYGRYKTGQGLMGVLLDTVYQFSL
EALRTHGRDIHRHLTQAWETWMLTWQEEGDVYRGQSELVVRTINLCAGSC
SSEEQLLNMMSHPQYHRLSQLTNKICHRLRLFQSHKEYEGESINTENGGI
NEEEIEADMRELVEAVIYNEDDDVDGNIKQTFLTVAKTFYYTAYCNPHTI
SLHISKVLFESLF

>NjTPS-23

MGFRLLRLHGYPVSADVFENFKNGEEFFCFNGQSNQAVTGMYNLLRASQI
VLPGEKILEDAKKFSYQFLKHKRAANQILDKWIITKDLPGEVNYALDMPW
YASLSRVETRYYLEQYGGQDDVWIGKTLYRMSKVNNNTYMELAKLDYTNC
QAMHLEEWSRIKQWYEECKLGEYGASQESLVLLAYYLASASIYEPEKSKE
RIAWAKTTTLMETIMSYFGTKNMSIEQRRSFVRDFKKCSNNLHFTYGRYK
TGQGLMGVLLDTVYQFSLEALRTHGRDIHRHLTQAWETWMLTWQEEGDMY
RGQAELVVRTINLCAGSCSSEEQLLNMMSHPQYHRLSQLTNKICHRLRLF
QSHKEYEGESINTENGGINEEEIEADMRELVEAVIYNEDDDVDGNIKQTF
LTVAKTFYYTAYCNPHTISLHISKVLFESLF

>NjTPS-24

MLESMDDGEISVSAYDTAWVALVEDINGSGAPQFPSSLDWIADNQLPDGS
WGDRYIFSAFERIINTLACIIALRTWNIHHQKSEKGMLFIKENIGKLEEE
NEEHMPIGFEVAFPSLVEIAKNLGIQIFPEESAVLQEIYARRNLKLTRIP
KDIMHKIPTTLLHSLEGMKELEWEKLIKLQCKDGSFLFSPSSTAFALIHT
HDSNALSYLTNTVHKFNGGVPNVYPVDLFEHIWAVDRLQRLGISRHFKSQ
IKDCLDYVHKYWREEGICWARNSPVQDIDDTAMGFRLLRLHGYPVSADVF

ENFKNGEEFFCFNGQSNQAVTGMYNLLRASQIVLPGEKILEDAKKFSYQF
LKHKRAANQILDKWIITKDLPGEVNYALDMPWYASLSRVETRYYLEQYGG
QDDVWIGKTLYRMSKVNNNTYMELAKLDYTNCQAMHLEEWSRIKQWYEEC
KLGEYGASQESLVLLAYYLASASIYEPEKSKERIAWAKTTTLMETIMSYF
GTKNMSIEQRRSFVRDFKKCSNNLHFTYGRYKTGQGLMGVLLDTVYQFSL
EALRTHGRDIHRHLTQAWETWMLTWQEEGDMYRGQAELVVRTINLCAGSC
SSEEQLLNMMSHPQYHRLSQLTNKICHRLRLFQSHKEYEGESINTENGGI
KEEEIEADMRELVEAVIYNEDDDVDGNIKQTFLTVAKTFYYTAYCNPHTI
SLHISKVLFESLF

>NjTPS-25

MSIIIATNGTEHPIFRPLANFPPSLWGNLFTSFSMDNQAREIYAKEHEGL
KEKVRVMLLDTTNYKISEKINFINTVERLGVSYHFEKEIEELLHQMFDAH
SKLLDDIQEFDLFTLGIYFRILRQHGYKISCDVFNKLKDSNGEFKDELKD
DVNGMLSLYEATHVRTHGENILDEALIYTKAQLESMAAASLSPFLAKQVK
HALMQALHKGIPRIEARNYISVYEEDPNKNDLLSRFSKIDFNLVQMIHKQ
ELCDTFRWWKDLEFESKLSFARNRVVEAYLWTLSAYYEPKYSSARIILVK
IMLILSVTDDTYDAYGTLDELQLFTDAVQRLDMSSINQLPDYMKTIYKAL
LDLFDEIEDRLSKHETDHSYRVAYAKYVYKEIVRCYDMEYKWFNKNYVPA
FEEYMQKALVTSGNRLLITFSFLGMDEVATIQAFEWVKSNAKMIVSSNKV
LRLIDDIMSHEEEDERGHVATGIECFVKEHGLTREEVIVEFHKRIDDAWK
DINEEFITPNNLPIEILTRVLNLTRIGDVVYKYDDGYTHPEKALKDHIIS
LFVDPVSI

>NjTPS-26

MSIIIATNGTEHPIFRPLANFPPSLWGNLFTSFSMDNQAREIYAKEHEGL
KEKVRVMLLDTTNYKISEKINFINTVERLGVSYHFEKEIEELLHQMFDAH
SKLLDDIQEFDLFTLGIYFRILRQHGYKISCDVFNKLKDSNGEFKDELKD
DVNGMLSLYEATHVRTHGENILDEALIYTKAQLESMAAASLSPFLAKQVK
HALMQALHKGIPRIEARNYISVYEEDPNKNDLLSRFSKIDFNLVQMIHKQ
ELCDTFRWWKDLEFESKLSFARNRVVEAYLWTLSAYYEPKYSSARIILVK
IMLILSVTDDTYDAYGTFDELELFTDAIQRWDMSAINQLPDYMKNIYRAL
LDLYDEIDDQLSKQESNCSYGVTYSKEMFKEMVRSYEIENKWFTKKYVPA
FEEYMKNGLVTGGNRLLVISSFLGMGEVATIQAFKWVKSNPKMIIASNTI
VRLIDDIMSHEEEDERGHAATGIECYRKEHGLTKEEVVVEFYQMNENAWK
DINEEFTRPNNLSVDILMRALNLTRIAEVVYKYDDGYTHPEKTFKDYIIS
LFVDPITI

>NjTPS-27

MSCMRSISSPSQLLSKSYDNIIDSSSSSSRSFSWSFKSKNPAAAAACTSM

CLSSRSSPSAVVVPPTSNNGSFLKYLQQSTVLVPQEIDDNSRTMELIEET
RKELVKVREPVEKMRLIEALQRLGISYHFENEINIILENLSGGGGRHSDE
DLFTTSLRFRLLRHNGHHISNDVFEKFVDENGKFKESLKEDTMGMLSLYE
ASYMGANGEDILLQAMEFTKNHLKESLPLMESNLGKQVLQSLELPKNLRM
ARLEARRYIEEYSNESDHNLALLELAKLDYNQVQSLHQMELAEISRWWKH
LGLVDKLSFARDRPLECFLWTVGILPEPKDSGCRIELAKTIAILLVIDDI
FDTHGSYDELVLFTNAIRRFDLMINLFV

>NjTPS-28

MSCMRSISSPSQLLSKSYDNIIDSSSSSSRSFSWSFKSKNPAAAAACTSM
CLSSRSSPSAVVVPPTSNNGSFLKYLQQSTVLVPQEIDDNSRTMELIEET
RKELVKVREPVEKMRLIEALQRLGISYHFENEINIILENLSGGGGRHSDE
DLFTTSLRFRLLRHNGHHISNDVFEKFVDENGKFKESLKEDTMGMLSLYE
ASYMGANGEDILLQAMEFTKNHLKESLPLMESNLGKQVLQSLELPKNLRM
ARLEARRYIEEYSNESDHNLALLELAKLDYNQVQSLHQMELAEISRWWKH
LGLVDKLSFARDRPLECFLWTVGILPEPKDSGCRIELAKTIAILLVIDDI
FDTHGSYDELVLFTNAIRRFDLMINLFV

>NjTPS-29

MSCMRSISSPSQLLSKSYDNIIDSSSSSSRSFSWSFKSKNPAAAAACTSM
CLSSRSSPSAVVVPPTSNNGSFLKYLQQSTVLVPQEIDDNSRTMELIEET
RKELVKVREPVEKMRLIEALQRLGISYHFENEINIILENLSGGGGRHSDE
DLFTTSLRFRLLRHNGHHISNDVFEKFVDENGKFKESLKEDTMGMLSLYE
ASYMGANGEDILLQAMEFTKNHLKESLPLMESNLGKQVLQSLELPKNLRM
ARLEARRYIEEYSNESDHNLALLELAKLDYNQVQSLHQMELAEISRWWKH
LGLVDKLSFARDRPLECFLWTVGILPEPKDSGCRIELAKTIAILLVIDDI
FDTHGSYDELVLFTNAIRRWDLNAMEELPEYMKICYMALYNTTNEICYKV
LKENGWSVLPFLKTTWIDMIEGFMVEAKWLNNEEVPNLEEYIENGVTTAG
SYMALVHIFFLIGEGVNEDNVKLLLNPYPKLFSSAGRILRLWDDLGTSKE
EQERGDVASSIQLFMKENNITCEEEARNQIIQIVQNLWKELNGELMAPNA
LPLPIIKACLNMARASQVVYQHDGDSYFSNVDNYVQSLFYTPIRM

>NjTPS-30

MSCMRSISSPSQLLSKSYDNIIDSSSSSSRSFSWSFKSKNPAAAAACTSM
CLSSRSSPSSAVVVPPTSNNGSFLKYLQQSTVLVPQEIDDNSRTMELIEE
TRKELVKVREPVEKMRLIEALQRLGISYHFENEINIILENLSGGGGRHSD
EDLFTTSLRFRLLRHNGHHISNDVFEKFVDENGKFKESLKEDTMGMLSLY
EASYMGANGEDILLQAMEFTKNHLKESLPLMESNLGKQVLQSLELPKNLR
MARLEARRYIEEYSNESDHNLALLELAKLDYNQVQSLHQMELAEISRWWK
HLGLVDKLSFARDRPLECFLWTVGILPEPKDSGCRIELAKTIAILLVIDD

IFDTHGSYDELVLFTNAIRRFDLMINLFV

>NjTPS-31

MSCMRSISSPSQLLSKSYDNIIDSSSSSSRSFSWSFKSKNPAAAAACTSM
CLSSRSSPSAVVVPPTSNNGSFLKYLQQSTVLVPQEIDDNSRTMELIEET
RKELVKVREPVEKMRLIEALQRLGISYHFENEINIILENLSGGGGRHSDE
DLFTTSLRFRLLRHNGHHISNDVFEKFVDENGKFKESLKEDTMGMLSLYE
ASYMGANGEDILLQAMEFTKNHLKESLPLMESNLGKQVLQSLELPKNLRM
ARLEARRYIEEYSNESDHNLALLELAKLDYNQVQSLHQMELAEISRWWKH
LGLVDKLSFARDRPLECFLWTVGILPEPKDSGCRIELAKTIAILLVIDDI
FDTHGSYDELVLFTNAIRRFDLMINLFV

>NjTPS-32

MISSSSVRSLYFPKTNIITSKVPSLLINNINVPSNNSSIRACISMSSLPV
SKSTSSSTAAPLIRDNGSLLKFITQTPQVEVDESKRIMELVETTRRTLRK
ASSDPTDKMKLIDSLQRLGLNHHFEEDINVVLQEFANEQKNTNEDLFTTS
LRFRLLRHNGYNVTPDIFNKFTEKNGKFKESLSEDTIGILSLYEASYLGA
KGEEILSEAIKFSESKLRESAGHVAPQIRRQILQSLELPRHLRMARLESR
RFIEEDYSKEIGCDLSLLELAKLDFNYVQSLHQMELAEISRWWKQLGLAD
KLPFARDRPLECFLWTVGLLPEPKHSECRIELAKTIAVLLVIDDIFDTYG
SFDQLVLFTNAIRRWDLDAMEELPEYMKICYMALYNTTNEICYKVLKENG
WSVLPYLKRTWIDMIEGFMVEAEWLNSGQVPNLEEYIENGVTTAGSYMAL
VHIFFLIGDGVTDDNVKLLLDPYPKLFSSAGRILRLWDDLGTAKVTFN

>NjTPS-33

MCLSSRSSPSAVVVPPTSNNGSFLKYLQQSTVLVPQEIDDNSRTMELIEE
TRKELVKVREPVEKMRLIEALQRLGISYHFENEINIILENLSGGGGRHSD
EDLFTTSLRFRLLRHNGHHISNDVFEKFVDENGKFKESLKEDTMGMLSLY
EASYMGANGEDILLQAMEFTKNHLKESLPLMESNLGKQVLQSLELPKNLR
MARLEARRYIEEYSNESDHNLALLELAKLDYNQVQSLHQMELAEISRWWK
HLGLVDKLSFARDRPLECFLWTVGILPEPKDSGCRIELAKTIAILLVIDD
IFDTHGSYDELVLFTNAIRRFDLMINLFV

>NjTPS-34

MNTCIQALSPPPPTKAIQLRPIANFHPSIWGNYFLKYASDHHTQSDDGTD
EQHGQLKEDVRKKLVVNDERAGEQLMLIDAIQRLGVAYHFQTEIDVVLNN
QLLKFNEDDDLYMVSLRFRLLRQQGHHVSSGVFEKFKDVEGRFKEILIND
VRGLLSLYESTHMRVHKEEILEEALQFTTTHLEHVVKASLTDTILLSQVV
HALNMPIRKGLTRIEARNYIPIYQQDKSHDETLLKFAKLDFNMLQKVHQR

ELGDITRWWKDLNVAEKLPYARDRLVEGYFWILGVYFEPRYSRARKILTK
VFSLISLIDDTYDSYGTFEELSLFTDAIQRWDVNAKNQLPEYMRHIYGEL
LDVYNAMEEELSKEGISYRIDYAKQTMKQQVRTYFDEAIWYNNGYVPTME
EYLKVALVSCGYIILATTSFVGMGVSDVTKQDFDWVSSNPLIVQASSVIC
RLTDDDVGHEFEQERGHVASAVECYMKQHNATKEEALVEFKKRITNAWKD
MNRECLHPLPVPMHLLERVPNFARFMYLFYKGEDCYTHSETRMKSFITSL
LVESAPN

>NjTPS-35

MDRSIQASSAPLPLSVLEPTRPIANFHPTIWGNYFLKFASDPGTNDDSDI
NQQIAQMKEDVRKMMVNSGDRREQQLKLIDEIQRLGVSYHFKSEIDVVLN
DHLLTLNDNGGDLYMESLRFRLLRQHGHNVSCDVFEKFKDGEGRFKEYLT
DDVRGLLSLYEATHMRVHKEEILDEALEFTTSHLEQVVKYSLSDHVLASK
VVHALKMPIRKGLTRLEARHYIPIYQMDNSHDETLLKFAKLDFNKLQKLH
QSELGDMTRWWKDFNVAEKLPYARDRFVECYFWALGVYFEPQYSHARRML
TKVIAFISLIDDTYDSYGTFEELSLFTDAIQRWDVNAKNQLPEYMRHIYG
ELLDVYNAMEEELSKEGISYRIDYAKQTMKQQVRTYFDEAIWYNNGYVPT
MEEYLKVALVSCGYIMAATTSFVGMGVKAVPKQAFDWVSSNPLMVQASSI
INRLTDDRVGHELEQQRGHVASGVECYMKQHNATEEEVLVEFNKRITSAW
KDMNQECLHPFPVPIHLLERVLNLARFMNIFYKDEDCYTHSNTRMKGIIT
SILIESIPS

>NjTPS-36

MNTCIQALSPPPPTKAIQLRPIANFHPSIWGNYFLKYASDHHTQSDDGTD
EQHGQLKEDVRKKLVVNDERAGEQLMLIDAIQRLGVAYHFQTEIDVVLNN
QLQLLNNQDDDLHMVSLRFRLLRQHGHNVSCGVFGKFKDIEGRFKECLMD
DVRGLLSLYESTHMRVHKEDILEEALEFTTTHLEQVVKSPFSCSVLVSQV
VHALNMPIRKGLTRIEARHFIPIYQQDESHDETLLKFAKLDFNMLQKVHQ
REVADITMWWKDLNVSEKLPYARDRAVECYFWILGVYFEPQYSRARRILT
KVICMTSLIDDTYDSYGTFEELSLFTDAIQRWDVNAKNQLPEYMRHIFGE
LLDVYGAMEEELSKEGISYRVDYAKQIMIQLVTAYNHEAIWYHDGYVPTL
EEYLEVALVSCGYIMAATTSFVGMGVKAVPKQAFDWVSSNPLMVQASSII
NRLTDDRVGHELEQQRGHVASGVECYMKQHNATEEEVLVEFNKRITSAWK
DMNQECLHPFPVPIHLLERVLNLARFMNIFYKDEDCYTHSNTRMKGIITS
ILIESIPS

>NjTPS-37

MANHYTTTDKWSITQEYLNFEDTTANCGNFYVENEQKLTQIRRKLMTVDE
DPLDCLKLVDAIHKLGIEYHFQDEINDFTERHYTTTMTKNVVPIQQGSLF
EGSLSFRLLRQQGYYVSANLFKNCYDGQGKFNTKLGQDIMGLMGLYEASQ

LNIEGEDILEEAGIISRRILNSLIGHVNNHENRTIMHMLSHPYHKSLARS
TAKDFIKVNNVDFGLNVTELPVLQELAKMDLDLVQSIHQKELLEISKWWK
ELGLGKELKFARNQPLKWHMWSIAALTNPSLSKQRIELTKPISLIYIIDD
IFDVYGTLDELIIFTKVVNRWEVSTMEQLPDYMKICFRALYDVTNEISYS
VYQDYGWNPVGSLRKAWGSLFDAFLVEAKWFASGNVPKSEEYLKNGIVSS
GANVVLVHMFFLLGEGTNKKNAKIVEDYPDIITYVATILRLWDDLGSSKV
KKLNNSALWTLSLSN

>NjTPS-38

MAIPTPQDHIIVRRVADYHPPIWDYDYVQSLTSKYLGDSYQKRADKLKEE
VRNMLNKVEDPLSKLELVDTLQRLGIYYHFEDEIKRILQSIHNDDNYSSD
EENKEDLYTTSLKFRLLRQHGYDIPQEVFSTFKDESGTFKVHVPEDIKGI
LSFYESTFLSTRGESILDEAREFTTQNMKEYLKKIIDINKSDDVMATQVS
HALEMPLHWRMLRLETRWFIDVYEKTDNKNEILLEFAKLDYNMVQAIHQK
DLKYTSRWWKSTKLGEKLSFARDRLEENFFWNVGFTFEPQFEYCRRMETK
LLSLITIIDDIYDIYGTLDELQLFTNAVERWDINAMEQLPDYMKTCFLSL
YNTINEFAYDALKEQNVNVISILKNSWADLCNSYFIEAKWYHTGYKPNLD
EYLENARISVSAPLILTHAYYFLTYPHPNVALECFEKYSSLICSTSIIVR
LADDLATSQSEMKRGDTPKSIQCYMYETGASEEEAREYIRYLISETWKKM
NEDRVVNKDDSLFSPIFVEMALNIARMGQCIYDHGDGFGIANRETMDRVT
SLFVEPISL

>NjTPS-39

MAYSLLSTFHLSTIPSNDKLLPPRPQLTSIRTSKGSKSSKCMAIPTPQDH
IIVRRVADYHPPIWDYDYVQSLTSKYLGDSYQKRADKLKEEVRNMLNKVE
DPLSKLELVDTLQRLGIYYHFEDEIKRILQSIHNDDNYSSDEENKEDLYT
TSLKFRLLRQHGYDIPQEVFSTFKDESGTFKVHVPEDIKGILSFYESTFL
STRGESILDEAREFTTQNMKEYLKKIIDINKSDDVMATQVSHALEMPLHW
RMLRLETRWFIDVYEKTDNKNEILLEFAKLDYNMVQVIHQEDLKYTSRWW
KSTKLGEKLSFARDRLEENFFWNVGFTFEPQFEYCRRMETKLLSLITIID
DIYDIYGTLDELQLFTNAVERWDINAMEQLPDYMKTCFLSLYNTTNETAY
DALKEQNVNIISFLKNSWADLCNSYFIEAKWYHTGYKPNLDEYLENARIS
VTAPLILTHAYYFLTNPHPNVALECFEKYSSLICSTSIIVRLADDLATSQ
SEMKRGDTPKSIQCYMYETGASEEEAREYIRYLISETWKKMNEDRVVNKD
DSLFSPIFVEMALNIARMGQCIYDHGDGFGIANRETMDRVRSLFIEPISL

>NjTPS-40

MAIPTPQDHIIVRRVADYHPPIWDYDYVQSLTSKYLGDSYQKRADKLKEE
VRNMLNKVEDPLSKLELVDTLQRLGIYYHFEDEIKRILQSIHNDDNYSSD
EENKEDLYTTSLKFRLLRQHGYDIPQEVFSTFKDESGTFKVHVPEDIKGI

LSFYESTFLSTRGESILDEAREFTTQNMKEYLKKIIDINKSDDVMATQVS
HALEMPLHWRMLRLETRWFIDVYEKTDNKNEILLEFAKLDYNMVQAIHQK
DLKYTSRWWKSTKLGEKLSFARDRLEENFFWNVGFTFEPQFEYCRRMETK
LLSLITIIDDIYDIYGTLDELQLFTNAVERWDINAMEQLPDYMKTCFLSL
YNTINEFAYDALKEQNVNVISILKNSVIKFIYLF

>NjTPS-41

MALAYSHLSTFHLSTIPSNDKLLPPPQLTSIRTSKGSKSSKCMAIPTPQD
HIIVRRVADYHPPIWDYDYVQSLTSKYLGDSYQKRADKLKEEVRNMLNKV
EDPLSKLELVDTLQRLGIYYHFEDEIKRILQSIHNDDNYSSDEENKEDLY
TTSLKFRLLRQHGYDIPQEVFSTFKDESGTFKVHVPEDIKGILSFYESTF
LSTRGESILDEAREFTTQNMKEYLKKIIDINKSDDVMATQVSHALEMPLH
WRMLRLETRWFIDVYEKTDNKNEILLEFAKLDYNMVQVIHQEDLKYTSRW
WKSTKLGEKLSFARDRLEENFFWNVGFTFEPQFEYCRRMETKLLSLITII
DDIYDIYGTLDELQLFTNAVERWDINAMEQLPDYMKTCFLSLYNTTNETA
YDALKEQNVNIISFLKNSWADLCNSYFIEAKWYHTGYKPNLDEYLENARI
SVTAPLILTHAYYFLTNPHPNVALECFEKYSSLICSTSIIVRLADDLATS
QSEMKRGDTPKSIQCYMYETGASEEEAREYIRYLISETWKKMNEDRVVNK
DDSLFSPIFVEMALNIARMGQCIYDHGDGFGIANRETMDRVRSLFIEPIS
L

>NjTPS-42

MIYELGWNWDELQLIAQGTLAGHLLECGSQLTGGYFMHPGDKYRDIPFEE
LLDLSLPYAEINFNGDLCVAKAEGSGGILNVSTCSQQLLYEIGDPSAYIT
PDVIIDFRDVTFRPLSRNKVLCVGAKPSDRLIPEKLLLLSPMGIGWKGWG
EISYGGYECVKRAKAAEFLVRAWMEEVYPGINKCIISYIIGVDSLSATEI
DNVTLSGIRDIRLRMDGLFKQEEHAIRFIKEFIALYTNGPAGGGGISTGH
KKETTLRKALITREDVHYHVTAKEYKMIESESDSTVPKTDVKENHADKIR
ISTSIQTETKTNSCMEEEFSESEIQTGPAPSGLKIPLYDIAHSRTGDKGN
DLNFSIIPHFLPDIERLKKVITPEWVKKVVSVLANTTSFPDFEEIERREK
WVEENVKVEIYEVKGISSLNVVVREILDGGVNCSRRIDRHGKCVSDLVLC
QRVVLPPP

>NjTPS-43

MDDTVSSSDSAQNPKSSDHSVNSRCTEDSDSDGEEAHIVNCIIELRKNPQ
RRREKVYIGCGAGFGGDRPLAALKLLERVEELNYLVLECLAERTLLDRYE
SVESGGVGYDPRISEWMNLLLPLAVQRDVCIITNMGAHDSHGAREKVLEI
AKELGISVNVGIAYQFALTKAAMVPRMGEYRCSTYLGAAPIVECLEKYKP
NVIITSRVADASLFLAPMIYELGWNWDELQLIAQGTLAGHLLECGSQLTG
GYFMHPGDKYRDIPFEELLDLSLPYAEINFNGDLCVAKAEGSGGILNVST

CSQQLLYEIGDPSAYITPDVIIDFRDVTFRPLSRNKVLCVGAKPSDRLIP
EKLLLLSPMGIGWKGWGEISYGGYECVKRAKAAEFLVRAWMEEVYPGINK
CIISYIIGVDSLSATEIDNVTLSGIRDIRLRMDGLFKQEEHAIRFIKEFI
ALYTNGPAGGGGISTGHKKETTLRKALVMSLYNDIMCA

>NjTPS-44

MKLSIRKYLHLKQIIDFRDVTFRPLSRNKVLCVGAKPSDRLIPEKLLLLS
PMGIGWKGWGEISYGGYECVKRAKAAEFLVRAWMEEVYPGINKCIISYII
GVDSLSATEIDNVTLSGIRDIRLRMDGLFKQEEHAIRFIKEFIALYTNGP
AGGGGISTGHKKETTLRKALITREDVHYHVTAKEYKMIESESDSTVPKTD
VKENHADKIRISTSIQTETKTNSCMEEEFSESEIQTGPAPSGLKIPLYDI
AHSRTGDKGNDLNFSIIPHFLPDIERLKKVITPEWVKKVVSVLANTTSFP
DFEEIERREKWVEENVKVEIYEVKGISSLNVVVREILDGGVNCSRRIDRH
GKCVSDLVLCQRVVLPPP

>NjTPS-45

MDDTVSSSDSAQNPKSSDHSVNSRCTEDSDSDGEEAHIVNCIIELRKNPQ
RRREKVYIGCGAGFGGDRPLAALKLLERVEELNYLVLECLAERTLLDRYE
SVESGGVGYDPRISEWMNLLLPLAVQRDVCIITNMGAHDSHGAREKVLEI
AKELGISVNVGIAYQFALTKAAMVPRMGEYRCSTYLGAAPIVECLEKYKP
NVIITSRVADASLFLAPMIYELGWNWDELQLIAQGTLAGHLLECGSQLTG
GYFMHPGDKYRDIPFEELLDLSLPYAEINFNGDLCVAKAEGSGGILNVST
CSQQLLYEIGDPSAYITPDVIIDFRDVTFRPLSRNKVLCVGAKPSDRLIP
EKLLLLSPMGIGWKGWGEISYGGYECVKRAKAAEFLVRAWMEEVYPGINK
CIISYIIGVDSLSATEIDNVTLSGIRDIRLRMDGLFKQEEHAIRFIKEFI
ALYTNGPAGGGGISTGHKKETTLRKALITREDVHYHVTAKEYKMIESESD
STVPKTDVKENHADKIRISTSIQTETKTNSCMEEEFSESEIQTGPAPSGL
KIPLYDIAHSRTGDKGNDLNFSIIPHFLPDIERLKKVITPEWVKKVVSVL
ANTTSFPDFEEIERREKWVEENVKVEIYEVKGISSLNVVVREILDGGVNC
SRRIDRHGKCVSDLVLCQRVVLPPP

>NjTPS-46

MDDTVSSSDSAQNPKSSDHSVNSRCTEDSDSDGEEAHIVNCIIELRKNPQ
RRREKVYIGCGAGFGGDRPLAALKLLERVEELNYLVLECLAERTLLDRYE
SVESGGVGYDPRISEWMNLLLPLAVQRDVCIITNMGAHDSHGAREKVLEI
AKELGISVNVGIAYQFALTKAAMVPRMGEYRCSTYLGAAPIVECLEKYKP
NVIITSRVADASLFLAPMIYELGWNWDELQLIAQGTLAGHLLECGSQLTG
GYFMHPGDKYRDIPFEELLDLSLPYAEINFNGDLCVAKAEGSGGILNVST
CSQQLLYEIGDPSAYITPDVIIDFRDVTFRPLSRNKVLCVGAKPSDRLIP
EKLLLLSPMGIGWKGWGEISYGGYECVKRAKAAEFLVRAWMEEVYPGINK

CIISYIIGVDSLSATEIDNVTLSGIRDIRLRMDGLFKQEEHAIRFIKEFI
ALYTNGPAGGGGISTGHKKETTLRKALITREDVHYHVTAKEYKMIESESD
STVPKTDVKENHADKIRISTSIQTETKTNSCMEEEFSESEIQTGPAPSGL
KIPLYDIAHSRTGDKGNDLNFSIIPHFLPDIERLKKVITPEWVKKVVSVL
ANTTSFPDFEEIERREKWVEENVKVEIYEVKGISSLNVVVREILDGGVNC
SRRIDRHGKCVSDLVLCQRVVLPPP

>NjTPS-47

MKLSIRKYLHLKQIIDFRDVTFRPLSRNKVLCVGAKPSDRLIPEKLLLLS
PMGIGWKGWGEISYGGYECVKRAKAAEFLVRAWMEEVYPGINKCIISYII
GVDSLSATEIDNVTLSGIRDIRLRMDGLFKQEEHAIRFIKEFIALYTNGP
AGGGGISTGHKKETTLRKALITREDVHYHVTAKEYKMIESESDSTVPKTD
VKENHADKIRISTSIQTETKTNSCMEEEFSESEIQTGPAPSGLKIPLYDI
AHSRTGDKGNDLNFSIIPHFLPDIERLKKVITPEWVKKVVSVLANTTSFP
DFEEIERREKWVEENVKVEIYEVKGISSLNVVVREILDGGVNCSRRIDRH
GKCVSDLVLCQRVVLPPP

>NjTPS-48

MANHYTTTDKWSITQEYLNFEDTTANCGNFYVENEQKLTQIRRKLMTVDE
DPLDCLKLVDAIHKLGIEYHFQDEINDFTERHYTTTMTKNVVPIQQGSLF
EGSLSFRLLRQQGYYVSANLFKNCYDGQGKFNTKLGQDIMGLMGLYEASQ
LNIEGEDILEEAGIISRRILNSLIGHVNNHENRTIMHMLSHPYHKSLARS
TAKDFIKVNNVDFGLNVTELPVLQELAKMDLDLVQSIHQKELLEISKWWK
ELGLGKELKFARNQPLKWHMWSIAALTNPSLSKQRIELTKPISLIYIIDD
IFDVYGTLDELIIFTKVVNRWEVSTMEQLPDYMKICFRALYDVTNEISYS
VYQDYGWNPVGSLRKAWGSLFDAFLVEAKWFASGNVPKSEEYLKNGIVSS
GANVVLVHMFFLLGEGTNKKNAKIVEDYPDIITYVATILRLWDDLGSSKD
ENQDGNDGSYIICYMKENEDVSVEKARQHVYKMISNAWKQLNKEWLLQNR
YSTTFKKASMNLARMVSLMYSYDENQSLPKLEEYMKSVL

>NjTPS-49

MDSYLNASSAPPPKKNMQEPVRPIANFHPSVWGNYFLKYASNPEQSDGGA
EEQHEQLKEALRKKLVVNVANERAGEQLKLIDAIQRLGVAYQFENEIDVV
LNNQLQLLNNQDDDLHMVSLRFRLLRQHGHNVSCGVFGKFKDIEGRFKEC
LMDDVRGLLSLYESTHMRVHKEDILEEALEFTTTHLEQVVKSPFSCSVLA
SQVVHALKLPIRKVLTRIEARHFIPIYQQDESHDETLLKFAKLDFNMLQK
VHQREVADITMWWKDLNVSEKLPYARDRAVECYFWILGVYFEPQYSHARR
MLTKVIAFISLIDDTYDSYGTFEELSLFTDAIQRWDVNAKNQLPEYMRHI

YGELLDVYNAMEEELSKEGISYRIDYAKQTMKQQVRTYFDEAIWYNNGYV
PTMEEYLKVALVSCGYIMLSTTSFVGMGVSVVPNQAFDWVTSNPLIVEAS
SVVNRLSDDKVGHKIEQERGHVVSAVECYMKQHNRTEEETIAEFKKRVTT
AWKDMNQECLHPLPVPIHLLERVLNLA

>NjTPS-50

MDSYLNASSAPPPKKNMQEPVRPIANFHPSVWGNYFLKYASNPEQSDGGA
EEQHEQLKEALRKKLVVNVANERAGEQLKLIDAIQRLGVAYQFENEIDVV
LNNQLQLLNNQDDDLHMVSLRFRLLRQHGHNVSCGVFGKFKDIEGRFKEC
LMDDVRGLLSLYESTHMRVHKEDILEEALEFTTTHLEQVVKSPFSCSVLA
SQVVHALKLPIRKVLTRIEARHFIPIYQQDESHDETLLKFAKLDFNMLQK
VHQREVADITMWWKDLNVSEKLPYARDRAVECYFWILGVYFEPQYSRARR
ILTKVICMTSLIDDTYDSYGTFEELILFTDAIQRWDVNAKNQLPEYMRHI
FGELLDVYGAMEEELSKEGISYRVDYAKQIMIQLVTAYNHEAIWYHDGYV
PTLEEYLEVALVSCGYIMLSTTSFVGMGVSVVPNQAFDWVTSNPLIVEAS
SVVNRLSDDKVGHKIEQERGHVVSAVECYMKQHNRTEEETIAEFKKRVTT
AWKDMNQECLHPLPVPIHLLERVLNLA

>NjTPS-51

MDSYLNASSAPPPKKNMQEPVRPIANFHPSVWGNYFLKYASNPEQSDGGA
EEQHEQLKEALRKKLVVNVANERAGEQLKLIDAIQRLGVAYQFENEIDVV
LNNQLQLLNNQDDDLHMVSLRFRLLRQHGHNVSCGVFGKFKDIEGRFKEC
LMDDVRGLLSLYESTHMRVHKEDILEEALEFTTTHLEQVVKSPFSCSVLA
SQVVHALKLPIRKVLTRIEARHFIPIYQQDESHDETLLKFAKLDFNMLQK
VHQREVADITMWWKDLNVSEKLPYARDRAVECYFWILGVYFEPQYSRARR
ILTKVICMTSLIDDTYDSYGTFEELILFTDAIQRWDVNAKNQLPEYMRHI
FGELLDVYGAMEEELSKEGISYRVDYAKQIMIQLVTAYNHEAIWYHDGYV
PTLEEYLEVALVSCGYIMAATTSFVGMGVKAVPKQAFDWVSSNPLMVQAS
SIINRLTDDRVGHELEQQRGHVASGVECYMKQHNATEEEVLVEFNKRITS
AWKDMNQECLHPLPVPIHLLERVLNLARFMNIFYKDEDCYTHSNTRMKDF
ITSLLIESVPS

>NjTPS-52

MSIIIATNGTEHPIFRPLANFPPSLWGNLFTSFSMDNQAREIYAKEHEGL
KEKVRVMLLDTTNYKISEKINFINTVERLGVSYHFEKEIEELLHQMFDAH
SKLLDDIQEFDLFTLGIYFRILRQHGYKISCDVFNKLKDSNGEFKDELKD
DVNGMLSLYEATHVRTHGENILDEALIYTKAQLESMAAASLSPFLAKQVK

HALMQALHKGIPRIEARNYISVYEEDPNKNDLLLRFSKIDFNLVQMIHKQ
ELCDTFRWWKDLEFESKLSYARNRVTEAYSLQE

>NjTPS-53

MISSSSVRSLYFPKTNIITSKVPSLLINNINVPSNNSSIRACISMSSLPV
SKSTSSSTAAPLIRDNGSLLKFITQTPQVEVDESKRIMELVETTRRTLRK
ASSDPTDKMKLIDSLQRLGLNHHFEEDINVVLQEFANEQKNTNEDLFTTS
LRFRLLRHNGYNVTPDIFNKFTEKNGKFKESLSEDTIGILSLYEASYLGA
KGEEILSEAIKFSESKLRESAGHVAPQIRRQILQSLELPRHLRMARLESR
RFIEEDYSKEIGCDLSLLELAKLDFNYVQSLHQMELAEISRWWKQLGLAD
KLPFARDRPLECFLWTVGLLPEPKHSECRIELAKTIAVLLVIDDIFDTYG
SFDQLVLFTNAIRRWDLDAMEELPEYMKICYMALYNTTNEICYKVLKENG
WSVLPFLKTTWIDMIEGFMVEAEWLNSGQVPNLEEYIENGVTTAGSYMAL
VHIFFLIGDGVTDDNVKLLLDPYPKLFSSAGRILRLWDDLGTAKEEQERG
DVSSSIQLYMKENNISSESEGRNSNQKIKNYIRLKTIHNKIFKI

>NjTPS-54

MISSSSVRSLYFPKTNIITSKVPSLLINNINVPNNNSSIRACISMSSLPV
SKSTSSSTAAPLIRDNGSLLIKFITQTPQVEVDESKRIMELVETTRRTLR
KASSDPTDKMKLIDSLQRLGLNHHFEEDINVVLQEFANEQKNTNEDLFTT
SLRFRLLRHNGYNVTPDIFNKFTEKNGKFKESLSEDTIGILSLYEASYLG
AKGEEILSEAIKFSESKLRESAGHVAPQIRRQILQSLELPRHLRMARLES
RRFIEEDYSKEIGCDLSLLELAKLDFNYVQSLHQMELAEISRWWKQLGLA
DKLPFARDRPLECFLWTVGLLPEPKHSECRIELAKTIAVLLVIDDIFDTY
GSFDQLVLFTNAIRRWDLDAMEELPEYMKICYMALYNTTNEICYKVLKEN
GWSVLPFLKTTWIDMIEGFMVEAEWLNSGQVPNLEEYIENGVTTAGSYMA
LVHIFFLIGDGVTDDNVKLLLDPYPKLFSSAGRILRLWDDLGTAKEEQER
GDVSSSIQLYMKENNISSESEGRKQIIEIIHNLWKDLNGELIGSNAMPLP
IIKTSFNMARTSQVVYQHEDDSYFSSVDNYVQSLFFTPI

>NjTPS-55

MISSSSVRSLYFPKTNIITSKVPSLLINNINVPSNNSSIRACISMSSLPV
SKSTSSSTAAPLIRDNGSLLTKFITQTPQVEVDESKRIMELVETTRRTLR
KASSDPTDKMKLIDSLQRLGLNHHFEEDINVVLQEFANEQKNTNEDLFTT
SLRFRLLRHNGYNVTPDIFNKFTEKNGKFKESLSEDTIGILSLYEASYLG
AKGEEILSEAIKFSESKLRESAGHVAPQIRRQILQSLELPRHLRMARLES
RRFIEEDYSKEIGCDLSLLELAKLDFNYVQSLHQMELAEISRWWKQLGLA

DKLPFARDRPLECFLWTVGLLPEPKHSECRIELAKTIAVLLVIDDIFDTY
GSFDQLVLFTNAIRRFDILPLFLKFTKF

>NjTPS-56

MISSSSVRSLYFPKTNIITSKVPSLLINNINVPSNNSSIRACISMSSLPV
SKSTSSSTAAPLIRDNGSLLTKFITQTPQVEVDESKRIMELVETTRRTLR
KASSDPTDKMKLIDSLQRLGLNHHFEEDINVVLQEFANEQKNTNEDLFTT
SLRFRLLRHNGYNVTPDIFNKFTEKNGKFKESLSEDTIGILSLYEASYLG
AKGEEILSEAIKFSESKLRESAGHVAPQIRRQILQSLELPRHLRMARLES
RRFIEEDYSKEIGCDLSLLELAKLDFNYVQSLHQMELAEISRWWKQLGLA
DKLPFARDRPLECFLWTVGLLPEPKHSECRIELAKTIAVLLVIDDIFDTY
GSFDQLVLFTNAIRRWDLDAMEELPEYMKICYMALYNTTNEICYKVLKEN
GWSVLPFLKTTVILTYLYFK

>NjTPS-57

MISSSSVRSLYFPKTNIITSKVPSLLINNINVPSNNSSIRACISMSSLPV
SKSTSSSTAAPLIRDNGSLLKFITQTPQVEVDESKRIMELVETTRRTLRK
ASSDPTDKMKLIDSLQRLGLNHHFEEDINVVLQEFANEQKNTNEDLFTTS
LRFRLLRHNGYNVTPDIFNKFTEKNGKFKESLSEDTIGILSLYEASYLGA
KGEEILSEAIKFSESKLRESAGHVAPQIRRQILQSLELPRHLRMARLESR
RFIEEDYSKEIGCDLSLLELAKLDFNYVQSLHQMELAEISRWWKQLGLAD
KLPFARDRPLECFLWTVGLLPEPKHSECRIELAKTIAVLLVIDDIFDTYG
SFDQLVLFTNAIRRWDLDAMEELPEYMKICYMALYNTTNEICYKVLKENG
WSVLPFLKTTWIDMIEGFMVEAEWLNSGQVPNLEEYIENGVTTAGSYMAL
VHIFFLIGDGVTDDNVKLLLDPYPKLFSSAGRILRLWDDLGTAKEEQERG
DVSSSIQLYMKENNISSESEGRKQIIEIIHNLWKDLNGELIGSNAMPLPI
IKTSFNMARTSQVVYQHEDDSYFSSVDNYVQSLFFTPI

>NjTPS-58

MISSSSVRSLYFPKTNIITSKVPSLLINNINVPSNNSSIRACISMSSLPV
SKSTSSSTAAPLIRDNGSLLKFITQTPQVEVDESKRIMELVETTRRTLRK
ASSDPTDKMKLIDSLQRLGLNHHFEEDINVVLQEFANEQKNTNEDLFTTS
LRFRLLRHNGYNVTPDIFNKFTEKNGKFKESLSEDTIGILSLYEASYLGA
KGEEILSEAIKFSESKLRESAGHVAPQIRRQILQSLELPRHLRMARLESR
RFIEEDYSKEIGCDLSLLELAKLDFNYVQSLHQMELAEISRWWKQLGLAD
KLPFARDRPLECFLWTVGLLPEPKHSECRIELAKTIAVLLVIDDIFDTYG
SFDQLVLFTNAIRRWDLDAMEELPEYMKICYMALYNTTNEICYKVLKENG

WSVLPFLKTTWIDMIEGFMVEAEWLNSGQVPNLEEYIENGVTTAGSYMAL
VHIFFLIGDGVTDDNVKLLLDPYPKLFSSAGRILRLWDDLGTAKVTFN

>NjTPS-59

MSCMRSISSPSQLLSKSYDNIIDSSSSSSRSFSWSFKSKNPAAAARTSMC
LSSRSSPSAVVVPPTSNNGSFLKYLQQSTVLVPQEIDDNSRTMELIEETR
KELVKVREPVEKMRLIEALQRLGISYHFENEINIILENLSGGGGRHSDED
LFTTSLRFRLLRHNGHHISNDVFEKFVDENGKFKESLKEDTMGMLSLYEA
SYMGANGEDILLQAMEFTKNHLKESLPLMESNLGKQVLQSLELPKNLRMA
RLEARRYIEEYSNESDHNLALLELAKLDYNQVQSLHQMELAEISRWWKHL
GLVDKLSFARDRPLECFLWTVGILPEPKDSGCRIELAKTIAILLVIDDIF
DTHGSYDELVLFTNAIRRWDLNAMEELPEYMKICYMALYNTTNEICYKVL
KENGWSVLPFLKTTWIDMIEGFMVEAKWLNNEEVPNLEEYIENGVTTAGS
YMALVHIFFLIGEGVNEDNVKLLLNPYPKLFSSAGRILRLWDDLGTSKEE
QERGDVASSIQLFMKENNITCEEEARNQIIQIVQNLWKELNGELMAPNAL
PLPIIKACLNMARASQVVYQHDGDSYFSNVDNYVQSLFYTPIRM

>NjTPS-60

MAANCPLLQFSSHSLEAKFHSNRLRVSSPSASIAKLNRTCRWSVIRCSSS
NGREPYSLDGGEKNAEKALEEKRRAELSARIASGEFTVQKTGTSFQSVLI
NGLSKLGVPSEIIEPLSKLINGGEDYPKIPEAKGAISAIRSEAFFIPLYE
LYLTYGGIFRLTFGPKSFLIVSDPSIAKHILRDNSKAYSKGILAEILEFV
MGTGLIPADGDVWRVRRRAIVPSLHQKYVAAMISLFGQATDRLCKKLDAA
AYDGEDVEMESLFSRLTLDIIGKAVFNYDFDSLTTDTGIVEAVYTVLREA
EDRSVSPIPFWEIPIWKDISPKQKKVNEALKLINGTLDNLIAICKRMVDE
EELQFHEEYMNETDPSILHFLLASGDDVSSKQLRDDLMTMLIAGHETTAA
VLTWTFYLLTKEPSVMAKLQNEVDSVLGDRIPTIEDMKKLRYTTRVINES
LRLYPQPPVLIRRSIIDDTLGDYPIKRGEDIFISVWNLHRCPKHWEDAEK
FNPERWPLDGPNPNETNQNFSYLPFGGGPRKCVGDMFASFEAVVAVAMLV
RRFNFQMALSAPPVEMTTGATIHTTQGLNMSVTRRINPPLVSTFPVLQVN
SPADADPLPKGEVSSAARS

>NjTPS-61

MTFLTTNIPTNENSFLLKTHTNGLIKNPKFYLPFKIKATHIAPQSYNTRS
IKIKELLEPKPLDYSPPAFKFEEYMKSKAKVVHKALDAAIPLQDPVKIHE
AMRYSLLAGGKRVRPILCIAACELVGGGETAAIPMACALEMIHTMSLIHD
DLPCMDNDDLRRGKPTNHKMFGEDTAVLAGDALLSLAFEHVATTATAVAT

SSNVTARVVRAIGELASAVGTEGLVAGQIVDICSEGKEVSLNELEFIHVH
KTAKLLEAAVVCGSLIGGGTMEEVERLRKYARHIGLLFQVVDDILDVTKS
SKELGKTAGKDLVTDKATYPKLMGMAKAEEFAGELVERAVEELSYFDPVK
AAPLYHLANYIAYRQN

>NjTPS-62

MWKLKIAEGHGPYLYSTNNFVGRQIWEFDPEAGTPEEREEVEKAREYFRN
NRRQGVHPCGDMIMRMQLIKESGIDLLSIQPVRLGEKEEVNYEAVTTAVK
KAVRLNRAIQAHDGHWPAENAGPMFFTPPLIIALYISGAINTILRQEHKT
EMIRYIYNHQNKDGGWGFYIEGHSTMIGSALSYVALRLLGESAYGGNGAV
ERARKWILDHGGAGGIPSWGKTYLSVLGVYEWEGCNPLPPEFWLFPSALP
YHPAKMWCYCRTTYMPMSYLYGKKFHGPITDLVLELRQEIHPVPYDQINW
NKQRHNCCKDDMYYPHSTIQDLLWDGLHYFSEPIIKYWPFNKLRQRGINR
AVELMRYGAEESRYITIGCVEKSLQMMCWWAENPNGDEFKHHLARVPDYL
WLAEDGMKMQSFGSQVWDCVLATQAIIASNMVEEYGDSLKKAHFYIKESQ
IKQNPSGDFSKMCRQFTKGSWTFSDQDQGWVVSDCTAESLKCLLMLSQMP
TEIAGEKADVERLYDAVNVLLYLQSPLSGGFAIWEPPVPQPYLQMLNPSE
IFADIVVEKEHVECTASIIQALLAFKRLHPGHREKEIETAVPKAVRFLEE
KQWPDGSWYGYWGICFIYGTFFVLGGLVSAGKTYKNSEAVRKAVKFFLST
QNDEGGWGESIKSCPSEVTSLTALILSKIYVQLYFTYLDVLFNIKTGIHT
TGRQPYKFCTNILGYARAYARWAG

>NjTPS-63

MVVLTKPATEHFCLVKSYKPTTFFPTIPVVDLSKPDSKHLLVKACQEFGF
FKVINHGISMDSIAKLESEAINFFNLPLSEKQKAIPPHPFGYGNKRIGSN
GDIGWLEYLLLTTNQEFNYQTLVSIFGENPEIFWGSIKDYVEAVKKMSCE
ILEMLAEGLKIEEKDVFSKLLMNEESDSLFRMNHYPPCPSSSSSSKSEIE
GGLRNNQQQIGFGEHTDPQIISVLRSNNTSGLQISLKDGSWISVPPDQNS
FFVNVGDSLQVMTNGRFKSVKHRVLANSRKARLSMIYFGGPPLSEKIAPL
PSLLIKGKEDSLYKEFTWFEYKKSAYNSRLADNRLGLFEKITAL

>NjTPS-64

MKMVKLSKELLFFEMKNYNNNIFLASLLSIGITIFFLILKKSTSSTVKVG
VPGRLGLPFVGETISFLFAVNSTKGVYNFVTLRRLRYGKWFKTRLLGKVQ
VFVPTATGAKMIFTNEFMKFNKGYLKSMKNAVGPKSLFCVTHESHKRIRR
LLSDPFSMNSLSGFVPKFDKLLCDRLKKLENDGNSFVVLDFNMKITFDAM
CDMLMSITDASLLTQIENDCAAVSASIISFPFMIPGTTYYKGMKARERLM

ETFEEIIGRRRSGKECHEDFLQTMLKRESYPADEKLDDSEIKDNLLTLII
AGQATTAAAMMWSLKFLDENRTVQDKLREESLAILRNKTNGALLTLEDLN
NMSYASKVVKETLRMSNGVLWFPRRALEDCIVDGVEIKKGWNVNIDATCI
HYDPTIYKDPLQFNPSRFDEIQKPYTYMPFALGARTCLGIDMAKMTMVVY
LQRLTSGYKWKVDDHDPRLGTKTHIPRLRSGLPITLTALENKN

>NjTPS-65

MKTVSPHYPFGITAHGNSSPKFDFSNLHFPLRVQNYVRSLCPSSTSFCTS
KGCVGRVYALPDFDEFFWDKVPTPILDTVENPMHLKNLSPKELKQLADDI
RSELSFIMSKTQKSLKPSLAAVELTIALHYVFHAPMDKILWDAGIQTYAH
KILTGRRSLIHTLREKDGLSGYTSRFESEFDPFGAAHGCNSVSAGLGLAV
ARDLKGKRNRVVTVISNGTTMAGQVYEAMSNAGYLDSDMIVILNDSRHSL
HPKINDASNEQINALSSTLIKLQSSKFFRRFREMAKGLTKRIGRGMYEWA
AKVDEYARGMIGPPGSTLFEELGLYYIGPVDGHNIEDLICVLHEVASLDS
TGPVFVHVITKEDQDSPLVQQTHPETNNQEEGSSPPSDSDSYISNGEYQT
YSDRFVEALIMEAERDKDIVAVHAGMGMEKSLQLFQQKFPDKFFEVGIAE
QHAVTFSAGLACGGLKPFCIIPSAFLQRAYDQVV

>NjTPS-66

MEKQEERRSYRVILVPCPFQGHISPMLQLGTLLYSKGFSITVAHIKINAP
DPSNHPDFTFLPLDADLTEFDTSALNMQGLVETINGKCEAPLRKCLAEMK
SEPHDQVVCIIYDTLMHYAESVAQYVKIPSIVLHTTSASALLAYFAIPRL
LAEGNIPLKESMLDEIVPDLDPLRYKDIPIHNGHLEGLLRVIAIVSNIKS
SSAIIWNTAEHLESSSLKQLQQHYNQIPLFSVGPLNVMAPSLATSLLEED
SSCKPWLDKKAPNSVIYVSLGSLATMDQNELTEMAWGLANSGQFVLWVVR
PRSIRDSEWVELLPEGFKEVIGDRICITKWAPQKEVLAHSAVGGFWSHCG
WNSTLESISEGVPVICRPFAGDQYVNARYLTHAWKVGIEFGDAYERVKIA
KAIRTLMVDKEGEEIRRRANDMKEKIKNCTMEGGSSYDSLNGLAELISSF
KVPK

>NjTPS-67

MEMISFIISFSLFSIFAYILSLINRRKLPPGPIGLPIVGNLFDIGPKPHE
SLAKLAKKHGPLMTIRLGSVTSVVATSAEMAREILQKNDEACSGRLVPDA
VTALRDNHLAVLWISAGEEWRIIRRALNTFLTHHQTLDTLTRLRHVAVEE
MVVHVKACSEKNLAVDIGKLAFATALNQMSSTCISRNVDDVEGFLSAVKT
LMVVDGKFNIADIFPWLKPFDPMSIRKRAKAAYGWFDEIIEGFIDRRLKQ
RESEQMRYGDLLDSLLDYSDENESLFNLKHIKVLLVDLFLAGTETTSNTT

EWAMTELMLHPHIMERVRQEVSGSIKMKGNIEEVDILELPYLQAVVKETM
RLHLAVPLLVPHKTEIDVKLSGYIIPKNTQVLINAWAIARDAEYWDNPTS
FMPERFINNFDSDFKGQNFTFLPFGSGRRMCPGISLAQRVVSLMIASLVY
YFDWKLPNGTEALDMNDTFGLTLQRSTPLLVVPTTRNKTNNM

>NjTPS-68

MWRLKIADGGDGDPYLYSTNNYVGRQTWEFEPESGTPEERAKVEQARLLF
WNNRYHVKPSSDLLWRYQFLEEKKFKQTIAQVKVEEGDEITYETATTTLR
RSVHFFSALQASDGHWPAENAGPLFFLPPLVMCLYITGHLNTVFSAEHRK
EILRYLYCHQNEDGGWGLHIEGHSTMFCTTLSYICMRILGEGPDGGLNNA
CARARKWILDHGSVTAIPSWGKTWLSILGVFDWSGTNPMPPEFWILPSFL
PMYPAKMWCYCRMVYMPMSYLYGKRFVGPITPLILQLREELYAQPYGDVN
WKKVRHHCAQEDIYYPHPLIQDLMWDSLYIMTEPFLTRWPFSKLREKALQ
TTMKHIHYEDENSRYITIGCVEKILCMLACWVEDPNGDYFKKHLARIPDY
IWVAEDGM

>NjTPS-69

MEEQGKSERRRLVLVPSPFQGHLSPMLQLGTILHSKGFSITIFHAKTNAP
DPSKHPNFEFRAFAEDVPKFNLSGIVQFISDLNTAYKAQFTEWLVEIKEE
NKLGNKVACVIYDTLMYSAEAVACEMKIPSIVLRTSSASYLLAYRAIPKL
KEEGYFPVQDSILQDLVPGLDPLRFKDLPFSNLSMEDALELLAMTSNIRS
SSAIIWNTIDYLEQSSLSKLQQQYYQVPFFPVGPLHIMGTHSSTSLLKED
NSCIDWLNKQATNSVIYTSLGSIATMDEKELVETAWGLANSEQPFLWTVR
PGSVRGSEWVELLPEGFLEKVGERGKIVKWAPQIEVLAHRAVGGFWSHCG
WNSTLESISEGVPMVCWPCFLDQKVNARYVTYVWRVGLELENVLERGEIE
RVVKRLMVENEGEEIRERAMKKKEKIEESMREGGSSYNSLNYVIDLILSF
EL

>NjTPS-70

MEEQGKSERRRLVLVPSPFQGHLSPMLQLGTILHSKGFSITILHAKTNAP
DPSNHPNFEFRVIAENLPKFNLAGEVFQFISDLNTACKAQFTEWLVETKK
ENKLCNQVACVIYDTIMYSAEVVACEMKIPSIVLRTSSASYLLAYRAIPK
LKEEGYFPVQDSILQDLVPGLDPLRFKDLPFSNLSMEDALELLAMTSNIR
SSSAIIWNTIDYLEQSSLSKLQQQYYQVPFFPVGPLHIMGTHSSTSLLKE
DNSCIDWLNKQATNSVIYTSLGSIATMDEKELVETAWGLANSEQPFLWTV
RPGSVRGSEWVELLPEGFLEKVGERGKIVKWAPQIEVLAHRAVGGFWSHC
GWNSTLESISEGVPMVCWPCFLDQKVNARYVTYVWRVGLELENVLERGEI

ERVVKRLMVENEGEEIRERAMKKKEKIEESMREGGSSYNSLNYVIDLILS
FEL

>NjTPS-71

MSVASSSSTSSVLKNIRSSFPLFHSQDGFSSNLLTAGFPINNKRKVGSRV
IMGSKYNNGSNNNEKEILVKDEEAQRLNQHFKKSLNYSGTKPATPILDTI
NYPNHMKNLSIKELEQLANELREEIVHTVSRIGGHLSSSLGVAELTVALH
HVFNTPEDKIIWDVGHQAYSHKILTGRRSKMSTIRQTCGLSGFPKRDESV
HDAFGVGHSSTSISAGLGMAVGRDLKGDDNHVISVIGDGAMTAGQAYEAM
NNAGYLESNLIIILNDNRQVSLPTATIDGPAPPVGALSKALTRLQSSRKL
RQLFEAAKGVTKQLGDQTHKLAAKVDSYVRGIAGAQGASLFEELGLYYIG
PVDGHNVEDLVYILNQVKSMPASGPVLIHIITEKGKGYPPAELAADKMHG
VVKFDPKTGKQSKEKSRTLSYTQYFAESLIAEAKGDDKIIAIHAAMGGGT
GLNSFQKHFPHRCFDVGIAEQHAVTFAAGLATEGFKPFCAIYSSFLQRGY
DQVVHDVDLQKLPVRFAIDRAGLVGADGPTHCGAFDTTFMASLPNMVVMA
PSCENELINMVATAAAINDRPSCFRYPRGNGIGSILQPNYKGTPLEVGKG
RILREGSRVAILGYGTIVQNCLEASKLLQMLGVSITVADARFCKPLDGNL
VRQLVQEHEILITVEEGSIGGFGSHVSQYMGLNGLLDGNLKWRAMMLPDR
YIDHGAQTNQIEEAGLSSKQIAATVLSLIGESRESIHLVNL

>NjTPS-72

MAAISSSSSSTSSVLKNSRGSIPSSQFNNGFPSKRLIPAFPINKRRKDGK
GVIMAANHHNNNNNGSSNNEKEVMVNGEETQIIKQRFKKSLNYSGDKPAT
PILDTINYPNHMKNLSIKELEELADELREEIVHTISKIGGHLSSSLGVAE
LTVALHHVFNTPQDKIIWDVGHQAYPHKILTGRRSKMSTIRQPSGLAGFP
KRDESVHDAFGVGHSSTSISAGLGMAIGRDLLGNDNHVISVIGDGAMTAG
QAYEAMNNAGYLESNLIIILNDNRQVSLPTATVDGPALPVGALSQSLTRL
QSSHKLRQLREPTNGVTKQLGDETHKLAAKVDSYVKGMTGGHGASLFEEL
GLYYIGPVDGHNIEDLVYILSQVKDMPASGPVLIHLITEKGKGYPPAELA
ADKMHGVVKFDPKTGKQSKASSKTLSYTQYFAESLIAEAEQDDKIVAIHA
AMGGGTGLNAFQKHFPHRCFDVGIAEQHAVTFAAGLATEGLKPFCAIYSS
FLQRGYDQVVHDVDLQKLPVRFAIDRAGLVGADGPTHCGAFDTTFMASLP
NMVVMAPSCENELINMVATAAAIDDRPSCFRYPRGNGIGSILQPNYKGTP
LEVGKGKILREGNRVAILGYGTIIQNCLEASELLQLHGVSITVADARFCK
PLDGNLVKQLAQEHEILITIEEGSIGGFSSHVAQYMCSNGLLDGNLKWRA
MMLPDRYIDHGAQTSQIEEAGLSSKQIAATVLSLMGESKESLHFINL

>NjTPS-73

MDTLLKTHNKLQFPHPLHGFTSKISSFSSTKPQNHEIRFGFGSKRSTLKL
GKITSLRASSSTLLELVQETKKENLDFELPLYDPSKGLVVDLAVVGGGPA
GLAVAQQVSEAGLSVCSIDPCPKLIWPNNYGVWVDEFEAMDLLDCLDTTW
SGAVVYIDENSKKELGRPYGRVNRKQLKSKMMQKCITNGVKFHQAKVIKV
IHEETKSFLICNDGVTVQASVVLDATGFSRCLVQYDKPYNPGYQVAYGIL
AEVEEHPFDVDKMVFMDWRDSHLNKNLELKEKNSKIPTFLYAMPFSSNKI
FLEETSLVARPGLQMEDIQDRMVARLKHLGIKVKSIEEDERCVIPMGGPL
PVLPQRVVGIGGTAGMVHPSTGYMVARTLAAAPIVAKSIVEYLGSEKSLL
GNELSGKVWRDLWPIERRRQREFFCFGMDVLLRLDLRGTRRFFSAFFDLE
PRYWHGFLSSRLFLRELLFFGLSLFSRASNTCRLEIMAKGTVPLVKMINN
LVQDSE

>NjTPS-74

MDVRRRPTTTNRHGEPLKPHQPKASDALPVPLYITNGLFFTLFFSVMYFL
LVRWREKIRNSTPLHIVTLSELAAIFALVTSVIYLLGFFGIGLVQSFVTK
PSLDDTWDVMDETHAERFMLEDDSRFKPCGAALEDQGTNIKKHKNLVPQS
SVETEQTAEESSIWTAEDEEIVQSVISGTIPSYSLESKLGDCRRAAAVRR
EALQRITGKSLEGLPLGGFDYASILGQCCEMPVGYVQIPVGIAGPLLLNG
KEFWVPMATTEGCLVASTNRGCKAIYTSGGATGVLLRDGMTRAPVVRFGS
VVRAAELKFFLEDPLNFETLALMFNKSSRFGRLQGIKCAIAGKNLYIRFT
CSTGDAMGMNMVSKGVQNVLDFLQSDFPDMDVMGISGNFCSDKKPAAVNW
IEGRGKSVVCEAIITEDVVKKVLKTNVAALVELNMLKNLAGSALAGALGG
FNAHASNIVSAVYIATGQDPAQNVESSHCITMMEAVNNGKDLHISVTMPS
IEVGTVGGGTQLASQSACLNLLGVKGASQEKAGSNSRLLATIVAGSVLAG
ELSLMSALAAGQLVKSHMKYNRSTKDLTKLSS

>NjTPS-75

MAFSFLPNFQISTISRQLPPALPQATIRKSKLKLAKCMASSTPTQPQTIV
RREANYHPPIWEHDYLQSLTSEYQGDTYKTRADKLKEEVRKMLNKVEDSS
SKLELVETLQRLGIYYHFEEEIKRILENIHNNGYTDMDKNKDLYATSLNF
RLLRQHGYDIPQEVFSTFKDESGTFKVHVPEDIKGILSFYESTFLSTRGE
SILDEAREFTTQNMKEYLKKIIDINKSDDIMATQVSHALEMPLHWRMLRL
ETRWFIDVYEKTDNKNEILLEFAKLDYNMVQVIHQEDLKYTSRWWKSTKL
GEKLSFARDRLEENFFWNVGFTFEPQFEYCRRMETKLLSLITIIDDIYDI

YGTLDELQLFTNAVERWDINAMEQLPDYMKTCFLSLYNTTNETAYDALKE
QNVNIISFLKNSWADLCKSYFIEAKWYHTGYKPNLDEYLENARISVTAPL
ILTHAYYFLTNPHPNVALECFEKYSSLIRSTSIILRLADDLATSESEMKR
GDTPTSIQCYMYETGASEEEAREYIRYLISETWKKMNEDRVVNKDDSLFS
PIFVEMALNIARMGQCIYDHGDGFGIANRETMDRVTSLFVEPISL

>NjTPS-76

MAFSFLPNFQISTISRQLPPALPQATIRKSKLKLAKCMASSTPTQPQTIV
RREANYHPPIWEHDYLQSLTSEYQGDTYKTRADKLKEEVRKMLNKVEDSS
SKLELVETLQRLGIYYHFEEEIKRILENIHNNGYTDMDKNKDLYATSLNF
RLLRQHGYDIPQDVFENFLDDNGKFKAYLSKDTKAMLCLYEASYLSTRKD
NILDEAREFASNHLKEYLKDNIDNNNFLEKLVVHSLELPLHWRVGRLEAR
WFIDMYENKEDMNPIVLQLAKLDYNMVQAVHLEDLKYVSRWWKSTSLGEN
LSFARDRLMENYIWTLEANFEPHLQYSRRMNTKVFALITTIDDVYDVYGT
LDELKLFTDILERWDVNAINQLPDYMKICFLVLFNAVNDMGYDALKNQNF
NIIPHLRNVVIIY

>NjTPS-77

MESTTILCSFAFIFSLYLFHSLLKFFCSSDRRRNLPLPPGTMGWPYIGET
FQLYSKNPNVFFASKVKKFGSIFKTHILGCPCVMISSPEAAKFVLVTKSD
IFKPTFPASKERMLGKQAIFFHQGDYHTKLRKLVLRSFMPEAIRSIVPDI
ESIAIDSLKSFEGRMINTFQEMKTFTFNVALLSIFGKDEVLYREDLKRCY
YILEKGYNSMPINLPGTLFHKSMKARKELAQILAKILSLRRESSEDRHDL
LGSFMGDKEGLTDEQIADNIIGVIFAARDTTASVMTWIVKYLAENPSVLQ
AVMKEQEAILKEKEECDNEGNNSKALTWADTKKMPLTTRVIQETLRVASI
LSFTFREAVEDVEFEG

>NjTPS-78

MASLRFSATVVPPSVAVSGGDLRDNSCFGNTIFSRKSSKKRDQLMSVVNV
ASKIPVVAPLPKREVAGGDGDDRRKLVAWKSVQQERWEGELVVEGEIPLW
LSGTYLRNGPGLWHLGDYDFRHLFDGYATLVRLHFENGRLVMGHRQIESD
AYKAAKQNNKLCYREFSEVPKHDNFLSYIGDLANLFSGASLTDNANTGVV
RLGDGRVVCLTETIKGSIVVDPDTLDTLGKFEYSDSLGGLIHSAHPIVTD
TEFLTLLPDLVNPGYLVVRMNPGTNERKVIGRVGCRGGPAPGWVHSFPVT
EHYIIVPEMPLRYCAQNLLKAEPTPLYKFEWHPESKGYMHVMCKASGNIV
ASVEVPLFVTFHFINAYEEKDEDGRVIAVVADCCEHNADTTILDKLRLQN
LRSYSGKEDVLPDAKVGRFIIPLDGSPKGELVAALNPEEHGKGMDMCSIN

PAFLGKKYRYAYACGAQRPCNFPNTLTKIDLVDKKAKNWWDKGAVPSEPF
FVARPGATEEDDGVVISMISDKNGEGYALLLDGSTFEEIARAKFPYGLPY
GLHGCWVPKN

>NjTPS-79

MMLASSHYGHKTTTASTSTVPYKKQSIEGTKERIRKMFKSNKVELSASPY
DTAWVAMVPSPNSSNAPCFPECLDWLLKNQLGNGSWGLPSHSHLLLKDTL
SSTLASVLALKRWNVGQSHINKGLHFMELNFQSAIDKNQHSPIGFDIIFP
GMLNYAKDLDLKLPLEPTLLNAMLHTRDLELNRCYESKAEAYLAYVSEGM
GKLQQDWEIVMKKYQRKNGSIFNSPATTAAVLTHHLPDAASLNYIRLLLD
KFGNAVPTVYPLDIYVRLCMIDNLERLGIDWHFRDEIQTVLDETYRCWLQ
GDEQIFTDISTCAIAFRLLRMNGYDVSSDALTQIAEEGNYLNSPGDRNLK
GISDELELYKASQIIISPDESSALRKQNLQSSNFLKQMLSDDSYCSDKLS
RSISQEVDDALNFPFCASLERMANRRYIEQYNVDTSTIRVLKTSYFSSNI
GNKDFLKLAVEDFNKCQSRHREDAAYLARWVIENRLDKLKFVRQKSFGYM
SFSAAATSFTPKLSDARMSWAKNALLTTVVDDFFDIGGSMDELLNLIYLV
DKWDNVDIESDCCSEHVGIIFSALQRGINEIAEQAFVYQERNVTSHIVEI
WLDLLKSMLREAEWSRDRYVPSMEEYMENGYVSFALGPILLPALYLVGPK
LSDESARSFQLKKLFRLMSNCGRLLNDIQGFKRESKEGKLNSVSLRMMMM
MNEGDTDEGIIVNELKMLVESYKEELLRIVIEEKESVLPRECKELFWKMT
KVVHQFYLKDDGFTSQHMMKAVNDVIYQPIILEEHQLE

>NjTPS-80

MLTLVIHCRCWTTMYLEVCSRIPALLYSETIYNYVQKQNQVIYLIIFLIL
FAGKLNRGLSVIDSYKLLKEGKELTDDEIFLTCALGWCIEWLQAYFLVLD
DIMDNSQTRRGQPCWYKIPKVGMIAVNDGILLRNHIPRILKKHFRSKPYY
VDLIDLFNEVEFQTASGQMIDLITTLEGEKDLSKYSLSIHRRIVQYKTAY
YSFYLPVACALLMIGENLDNHVNVKDILIEMGTYFQVQDDYLDCFGAPEV
IGKIGTDIEDFKCSWLVVKALELSNDEQKKFLYENYGKEDHASVSKVKEL
YRTLKLEDVFADYESKSYEKLIKFIEAHPSKAVQEVLKSFLGKIYKRKK

>NjTPS-81

MDVSTCAIAFRLLRMNGYDVSSDLLIRTAEENDCFSSSGGHLKGISDTLE
LYRASQIIISPDESNLRKHNSKSSHILKYALSNDSFCSDKLATYIRQEVD
DGLKFPFYASLERMENRRNIEQYSADCSVIRVLKTSYCSPNIGNKDFLKL
AIEDFNFIQSIHREEIKHVESWVIEGKLDKLKFARQREAYCLFSAAATFF
TPELSDARIIWAQNAVLTIVVDDFFDNAGSPHEFLNLIRLAEKWNVDIES

DCCSKEVGILFFAIHNANTEIADKAFICQGRSVTNHIVQIWLDYLKALWI
EAEWTRNKYVPSVDEYIENAYITFALGPIILPALYVVGEELSEEAVRSIE
FNKMFQLVSSCGRIINDTQTLEVIIHTHTHVK

>NjTPS-82

MRIGTNHGSLSDRIMSYYGDSPRGMVESAFEFARICRKLDFHNFVFSMKA
SNPVVMVQAYRLLVAEMYVQGWDYPLHLGVTEAGEGEDGRMKSAIGIGTL
LQDGLGDTIRVSLTEAPEQEIDPCRRLANLGMQATALQKGVAPFEEKHRH
YFDFQRRTGQLPVQKEGDEVDYRGVLHRDGSVLMSVSLDQLKTPELLYRS
LAAKLVVGMPFKDLATVDSILLREIPPVDDQDSRLALKRLIDISMGVITP
LSEQLTKPLLNAMVLVTLKELSTGAHKLLPEGTRLVVSVRGDESIEELEI
LKSVDATMIFHNLPYTEEKIGRVHAARRLFDYLSENSLDFPVIHHIKFPE
GIHRDDLVIGAGTNAGALLVDGFGDGVLLEASDQDFDFLRNTSFNLLQGC
RMRNTKTEYVSCPSCGRTLFDLQEISAEIREKTSHLPGVSIAIMGCIVNG
PGEMADADFGYVGGAPGKIDLYVGKTVVKRAIAMEQATDALIQLIKDHGR
WVDPAPLE

>NjTPS-83

MRIGTNHGSLSDRIMSYYGDSPRGMVESAFEFARICRKLDFHNFVFSMKA
SNPVVMVQAYRLLVAEMYVQGWDYPLHLGVTEAGEGEDGRMKSAIGIGTL
LQDGLGDTIRVSLTEAPEQEIDPCRRLANLGMQATALQKGVAPFEEKHRS
YFDFQRRTGQLPVQKEGEEVDFRGVLHRDGSVLMSVSLDQLKSPELLYKS
LAAKLVVGMPFKDLATVDSILVRELPPVDDKDARLALKRLIDISMGIITP
LSEQLTKPLPNAIVLVTLKELSTGAHKLLPEGTRIAVSVRGDESTEELEI
LKSIDATMLLHDLPYTDEKVGRVQAARRLFEYLSENELNFPVIHHIEFPE
GIHRDDLVIGAGTNAGALLVDGFGDGILLEAVDQDFDFLRNTSFNLLQGC
RMRNTKTEYVSCPSCGRTLFDLQEISAEIREKTSHLPGVSIAIMGCIVNG
PGEMADADFGYVGGAPGKIDLYVGKTVVKRAIAMEQATDALIQLIKDHGR
WVDPAPLE

>NjTPS-84

MATGSVSTSFTSLKSKDNGLGFNKSMDFVKICDTRRVKFSRSKAPVITNS
GSEIAELQPASEGSPLLVPRQKYCESTNKTVRRKTRTVMVGNVAIGSEHP
IRIQTMTTTDTKDVAGTVEQVMRIADRGADLVRITVQGRKEADACFEIKN
SLIQKNYTIPLVADIHFAPPIAMRVAECFDKIRVNPGNFADRRAQFEQLE
YTEDDYQKELEHIEQVFVPLVEKCKKYGRAMRIGTNHGSLSDRIMSYYGD
SPRGMVESAFEFARICRKLDFHNFVFSMKASNPVVMVQAYRLLLAEMYVH

GWDYPLHLGVTEAGEGEDGRMKSAIGIGTLLQDGLGDTIRVSLTEAPEQE
IDPCRRLANLGMQATALQKGVAPFEEKHRSYFDFQRRTGQLPVQKEGEEV
DFRGVLHRDGSVLMSVSLDQLKSPELLYKSLAAKLVVGMPFKDLATVDSI
LVRELPPVDDKDARLALKRLIDISMGIITPLSEQLTKPLPNAIVLVTLKE
LSTGAHKLLPEGTRIAVSVRGDESTEELEILKSIDATMLLHDLPYTDEKV
GRVQAARRYVLNISQKNLDLFNKIILYNSFFRKFIATTIKLLCTPHHTPH
TY

>NjTPS-85

MATGSVSTSFTSLKSKDNGLGFNKSMDFVKICDTRRVKFSRSKAPVITNS
GSEIAELQPASEGSPLLVPRQKYCESTNKTVRRKTRTVMVGNVAIGSEHP
IRIQTMTTTDTKDVAGTVEQVMRIADRGADLVRITVQGRKEADACFEIKN
SLIQKNYTIPLVADIHFAPPIAMRVAECFDKIRVNPGNFADRRAQFEQLE
YTEDDYQKELEHIEQVFVPLVEKCKKYGRAMRIGTNHGSLSDRIMSYYGD
SPRGMVESAFEFARICRKLDFHNFVFSMKASNPVVMVQAYRLLLAEMYVQ
GWDYPLHLGVTEAGEGEDGRMKSAIGIGTLLQDGLGDTIRVSLTEAPEQE
IDPCRRLANLGMQATALQKGVAPFEEKHRSYFDFQRRTGQLPVQKEGEEV
DFRGVLHRDGSVLMSVSLDQLKSPELLYKSLAAKLVVGMPFKDLATVDSI
LVRELPPVDDKDARLALKRLIDISMGIITPLSEQLTKPLPNAIVLVTLKE
LSTGAHKLLPEGTRIAVSVRGDESTEELEILKSIDATMLLHDLPYTDEKV
GRVQAARRLFEYLSENELNFPVIHHIEFPEGIHRDDLVIGAGTNAGALLV
DGFGDGILLEAVDQDFDFLRNTSFNLLQGCRMRNTKTEYVSCPSCGRTLF
DLQEISAEIREKTSHLPGVSIAIMGCIVNGPGEMADADFGYVGGAPGKID
LYVGKTVVKRAIAMEQATDALIQLIKDHGRWVDPAPLE

>NjTPS-86

MEPQSWSIILLALFLATTLFLLRHRPKKNFPPGPKAWPIIGNIHQIGPLP
HHSFHSLSETYGPLLHLHLGSRPVVVASSPHMAEQILKTHDQTFASRPAL
ASAKYTSYNSSNVLWAPYGPHWRQARKIYSTELLSPKKLESYDYIIEEEN
ILFIRRLYKTRGESIKVKEELTRYMLSNLSRVVMGNEYFGSIMKLEELGE
ILDEWFLLNGVINLGDWIPWLGFLDLQGYVKRMKSLHKKFDMFNNSVLSY
HKNKVNEKEDMVDVLLKLANQPNLEVKLTNDGVKGLVQDLLVGGTDTSAT
TVEWAMLELLKNPHILKKAIKEVEEVIGNQRWVEEKDFGELPYIESIIKE
TLRLHPLATLLAPHFAIQDCKIDGYDILKGTTVFINTWSIGRNPLYWDNP
LEFKPERFSGKNSNIDVKGLNFELLPFGSGRRMCPGYRLALNVVRSTLAN
LLHGFIWKLPNDIKIEDICMDEVYGIASHPKMSLDLVLEPRLPSTLYY

>NjTPS-87

MALNFLSPTTEIKGISFLDTSKSTYQIPKLQGGFGCMRKENGVKSVNRIS
CSSNPPPPPAWPGRAVVEPGRKVWDGPKPISIVGSTGSIGTQTLDIVAEN
PDKFNVVALAAGSNITLLADQVKTFRPQLVAVRNESLIGELREALADADY
KPEIVAGEQGVIEVARHPDCVTVVTGIVGCAGLKPTVAAIEAGKDIALAN
KETLIAGGPFVLPLAQKHNVKILPADSEHSAIFQCIQGLPEGALRRIILT
ASGGAFRDWPVEKLKDVKVADALKHPNWNMGKKITVDSATLFNKGLEVIE
AHYLYGANYDDIEIVIHPQSIIHSMIETQDSSILAQLGWPDMRLPILYTL
SWPERIYCSEVTWPRLDLCKLGSLTFKSPDNVKYPSMDLAYAAGRSGGTM
TGVLSAANEKAVEMFIDEKISYLDIFKVVELTCEKHQAELVTSPSLEEII
YYDLWARDYAASLQPSSSGLNAVLV

>NjTPS-88

MALNFLSPTTEIKGISFLDTSKSTYQIPKLQGGFGCMRKENGVKSVNRIS
CSSNPPPPPAWPGRAVVEPGRKVWDGPKPISIVGSTGSIGTQTLDIVAEN
PDKFNVVALAAGSNITLLADQVKTFRPQLVAVRNESLIGELREALADADY
KPEIVAGEQGVIEVARHPDCVTVVTGIVGCAGLKPTVAAIEAGKDIALAN
KETLIAGGPFVLPLAQKHNVKILPADSEHSAIFQCIQGLPEGALRRIILT
ASGGAFRDWPVEKLKDVKVADALKHPNWNMGKKITVDSATLFNKGLEVIE
AHYLYGANYDDIEIVIHPQSIIHSMIETQDSSILAQLGWPDMRLPILYTL
SWPERIYCSEVTWPRLDLCKLGSLTFKSPDNVKYPSMDLAYAAGRSGGTM
TGVLSAANEKAVEMFIDEKISYLDIFKVVELTCEKHQAELVTSPSLEEII
YYDLWARDYAASLQPSSSGLNAVLV

>NjTPS-89

MQAKPYHITPPKIPSPVKLPPATTVITSPEKVSQPISITVPTHRVISSPS
LVDDSVTAYWDYQFLFVSQRSETVDPITLRLVEGAVPPDFPLGTYYLTGP
GLFADDHGSTVHPLDGHGYLRAFTIDGVKSEVKFMARYIQTEAQSEERDS
VSGNWQFTHRGPFSVLKGGKKLGNTKVMKNVANTSVLKWNERLFCLWEGG
TPYEINPGSLDTIGEFDVIHSGDSSTGGEFKVRDVWDFAAEILKPILYGI
FNMPPKRLLSHYKIDASRNRLLIMSCNAEDMLLPRSHFTFYELDSNFKML
QTREFKIPDHLMIHDWAFTESHYILFGNRIRLDIPGSMTAVCGLSPMITA
LSLNPSKSTSPIYLLPRFPNNNEDEKKERDWKVAIEAPSQMWVLHVGNAF
ENKGQNGNSQIQIQASGCSYQWFNFQTMFGYDWKSGKLDPSMMNADGGEK
LLPHLVQVSIDLDDKGNCEKCDVDILNQWEKAADFPIVNQHFSGSQNKHI
YAATSLGSRKALPHFPFDTIVKLNTDNKSSTATWSVGSRRFIGEPIFVPK
GIDEDDGYLLVVEYAVSTQMCYLVILDAKNIGEDNALVARLEVPKHLNFP

LGFHGFWAPA

>NjTPS-90

MWGYCRTTYMPMSYLYRKKYHGPITNLVLALRQEIYPIAYSEINWNKQRH
NCCKEDLYYPHSFIQDLLWDSLHYFIEPIIKSWPFNKLSDRAIQRTMELM
HYGTKESRYINMGCVEKSLQMMCAWAENPNDVAFRYHLARIPDYLWVAED
GMKMQSFGSQLWDCVLATQAIMASNMVEEYGDSLKNANFYIKESQIKDNP
SGDFTKMCRQFTKGSWTFSDQDHGWTVSDCTAESLSCLLKLSQMPSKIVG
DKVDVERLYEAVDVLLYLQSPTSGGFAIWEPPVPQPYLQVLNPSELFADI
VVEKEHVECTGSIIQALVEFKHLHPGYRKKEIQISITKAIHFLETKQWHD
GSWYGYWGICFLYGTYFALKGFAAVGKTYNNSEAVRKAVQFLLSKQNKEG
GWGESFQSCPTEKFTPLDGNRTNLVQTSWAMLGLMLGGQAERDPTPLHKA
AKLLINAQMENGDFPQQEITGVYMKNCMLHYPEYKNIFPFWALGEYRKRI

>NjTPS-91

MVRILDLKPSMDAVYHWINPSPLDKNTEELIEKIRERFEKVDLSISAYDT
AWVAMVTSALRHQEPCFPGCLEWILENQKADGSWGLNLSHPSLLKDSLSS
TLACVIALQKLNVGEKHIQRGLVFIGSKKYAAVDKYQSSPIGFDINFPAM
IKYANNLGLNLPLDSAFIDLMLHNRDIELQRCKPRDLAYFAEGLVGESSY
DWEEIIKNGQGSNGSLFNSPAATAVALINTRNDKCYDYLNSLLKINHGKG
VPTIYPFHLYTRLCMVDTIDRLGINRHFANELKVILDETYRCWLQKSEEI
YSDVSCCSMAFRLLRKNGYHVSSDALEEFIDEEHFFSTLSPQFRNTSTVV
QLYRASQMSFFQNEPVLDKINEWTTNFLRHQLLNHEIFDDDLLREVNYAF
EYPIDNMPRLTNRRGIELYNTDSFRMLKTSYRCCSVNNEEFLVLSQQEFN
KTQQIHLEEYKQVEEWLKKHRIGGLEFEWHMVTSSYFLAASGYIIPELSD
ARIIWAKVSVLGTIIDDLFDIDGTQEELLNIVHLIQNWDGNPNISSLDYS
SERTEIMFLALTEIINEQAAIGLIRQGRCIKKELIQIWQNFCKSCFKEVE
WWANKSTPTLDKYLANGCQTIGIGLWSITFYCVGIQLSQDTLISEEYQII
YKHLGLIMRLFNDYQGVIDERDKVQRKMNGCLLLVSLSGGALTVEEARTE
VRKMIDISRKEVLRMMLTASSTTQKILMESFFCFHQQAYYLYTGNDEYRI
PSKKGLNDINRLLYEPLNLI

>NjTPS-92

MPSRISDASKTHPVINIDKHLDLHSMKELPDSHSWISLDGSPSGDSSGAD
NVTVPLIDLEDPNVIKLIGHACKTWGVFQIKNHGISNNLLDSMEGFAQKL
FNLPIQQKLKAARAPNGVSGYGVARISRFFSKLMWSEGFTIVGSPLQHAR
QLWPQDYNKFCDVVQEYEEEMKSLGGKLMRLMLGSLEIATKDVNWAGPNG

DFKASTAALQLNSYPACPDPDRAMGLAAHTDSTLLTILHQNNTSGLQVKH
EGSGWVTVDPLPDTLVVNVGDLLHILSNGLYLSVLHRAVVNRTRHRLSIA
YLYGPPSNVEISPLSKLVENGQTPLYRSVTWSEYLGTKAKHFDKALSAVR
LCVSLNGFGDANDQNSVIVG

>NjTPS-93

MVVNNFSFINTTTNLFSEIPIIPLTHPNAKKLIVKACEEFGFFKLVNHNV
SIDLMARLEDEALEFFNSPQSEKDQTAPPHPFGYGNKRIGHNGDIGWVEY
LLFTINDPQLNSNNSLSIFARNQQTLWCLINEYIAAVRKMACEVLELITD
GLEIKPRDVLSRMLSDENSDSIFRLNYYAPCVDHQQLREMNGRDIVGFGE
HTDPQIISVVKSNNINGLQICLKDGTWVAVPPDPYSFFINVDDSLQVMTN
GRFKSVKHRVLTNCIKSRVSMIYFGGPPLSENITPLTSLMEQTGEESLYK
HFTWSQYKNSAYNSKLGDDRLKPFEKP

>NjTPS-94

MAVVASAPGKVLMTGGYLILERPNAGIVLSTNARFYAIVKPLSHEIKLFG
DETEWTYVKLTSPQMSRETMYKLSLKILTIECASSSDSRNPFVEYAVQYA
VAAAYATLDKNKKDELHRLLLHGLDITILGSNDFYSYRNQIEARGLPLSP
ESLATLSPFTSITFNDEESNGGKNSKPEVAKTGLGSSAAMTTAVVAALLH
YLGVVNLEGSDKDEKPQEKGNSDLDLVHIIAQTAHCIAQGKVGSGFDVSS
AAFGSQRYVRFSPAVISSAQDPVDGKPLEDVIDDVIKGKWDHERTRFALP
PLMTLLLGEPGSGGSSTPSMVGAVKKWQKSDPQKSLETWTKLSDANSSLE
KQLNLLSNLAVQNWDAYESVINSCSMQVSQKWMEQTNELNQVEIVKALLG
ARDAMLLIRSLMRQMGNAADIPIEPESQTELLDETMKMEGVVFAGVPGAG
GFDAIFAVTLGDSSTLTKTWSSRNVLAMLVREDPRGVSLETTDPRINN

>NjTPS-95

MSIANLSTSWIQINSTYGNPNKSRSTSKFLIHSFNYNNKSLSLFFPFPKS
KSISHCSLSISSILTKEQTEEKDKQSSSSLFDFNSYMIQKANSVNQALDD
AVSVREPLKIHESMRYSLLAGGKRIRPMLCIAACELVGGDEATAMPAACA
VEMIHTMSLMHDDLPCMDNDDLRRGKPTNHKVFGEDVAVLAGDALLAFSF
EHIATETAGVSSERIVRAVHELAKCIGAEGLVAGQIVDICSEGMAGVGVD
HLEFIHVHKTAALLEGSVVLGALLGGGSDDEIEKLRKFARCIGLLFQVVD
DILDVTKSSLELGKTAGKDLVADKTTYPKLIGVEKSREFAEKLNKEAQDQ
LSGFDSRKAAPLIALANYIAYRDN

>NjTPS-96

MASQQKNVGILAIEIYFPPACIQQESLEAHDEVSKGKYTIGLGQDCMAFC
TEVEDVISMSLTAVTSLLEKYEIDPKQIGRLEVGSETVIDKSKSIKTFLM
QIFEESGNTDIEGVDSTNACYGGTAALFNCVNWVESSCWDGRYGLVVCTD
SAVYAEGPARPTGGAAAIAMLIGPDAPIAFESKFRASHMAHVYDFYKPNL
ASEYPVVDGKLSQTCYLVALDSCYKRFCSKYEKLEGKQFSIADADYFVFH
SPYNKLVQKSFARLTFNDFMRNASSISEDAKEKLAPFSTLTGDESYQNRD
LEKASQQVAKALYDKKVTPSTLIPKQIGNMYTASLYAAFVSLLHNKNSSL
DGNRVVMFSYGSGSTATMFSFHLNEGQHPFSLSNIAKVMNVGDKLKSRTE
FPPKKFVETLHLMEHRYGAKDFVTSKDCSLLAPGTFYLTEVDSKYRRFYA
KKTGDSTVTVNGSLSNGVH

>NjTPS-97

MASQQKNVGILAIEIYFPPACIQQESLEAHDEVSKGKYTIGLGQDCMAFC
TEVEDVISMSLTAVTSLLEKYEIDPKQIGRLEVGSETVIDKSKSIKTFLM
QIFEESGNTDIEGVDSTNACYGGTAALFNCVNWVESSCWDGRYGLVVCTD
SAVYAEGPARPTGGAAAIAMLIGPDAPIAFESKFRASHMAHVYDFYKPNL
ASEYPVVDGKLSQTCYLVALDSCYKRFCSKYEKLEGKQFSIADADYFVFH
SPYNKLVQKSFARLTFNDFMRNASSISEDAKEKLAPFSTLTGDESYQNRD
LEKASQQVAKALYDKKVTPSTLIPKQIGNMYTASLYAAFVSLLHNKNSSL
DGNRVVMFSYGSGSTATMFSFHLNEGQHPFSLSNIAKVMNVGDKLKSRTE
FPPKKFVETLHLMEHRYGAKDFVTSKDCSLLAPGTFYLTEVDSKYRRFYA
KKTGDSTVTVNGSLSNGVH

>NjTPS-98

MASSSPPICLPAAGNRSRLPTGLRICGSKRLVKFTTQTSVIRSDLDTNVS
DMSVNAPKGLFPPEPQHYRGPKLKVAIIGAGLAGMSTAVELLDQGHQVDI
YDQRSFIGGKVGSFVDKRGNHIEMGLHVFFGCYNNLFRLMKKVGAEKNLL
VKDHTHTFVNKGGEIGDLDFRFPVGAPLHGISAFLSTNQLKVYDKARNAL
ALALSPVVRALVDPDGAMRDIRNLDSISFSEWFMSKGGTRTSIQRMWDPV
AYALGFIDCDNISARCMLTIFSLFATKTEASLLRMLKGSPDVYLSGPIRD
YITEKGGRFHLRWGCREVLYDRSADGEMYVTGIAMSKATQKKIVKADAYV
AACDVPGIKRLVPAEWRQWDLFENIYKLDGVPVVTVQLRYNGWVTELRDL
ERSRQSRQATGLDNLLYTPDADFSCFADLALTSPEDYYREGEGSLLQCVL
TPGDPYMPLPNDEIIRRVTDQVLSLFPSSQGLEVTWSSVVKIGQSLYREG
PGKDPFRPNQKTPVKNFFLAGSYTKQDYIDSMEGATLSGRQASAYICDAG
EVLVALQKEISSISSPGLIFNNDELTLV

>NjTPS-99

MAATGNLIRINPNVSSRFKSSRLSPSTKKKFCARASASSRDAGDQEGKLV
IKREKENFKIDFSGEKPPTPLLDTINYPAHLKNLSTEDLEQLAAELRVEI
VYTVAKIGGHLSSSLGVIELAVALHHVFNTPDDRIIWDVGHQAYPHKILT
GRRSKMHTIRKTSGLAGFPKRDESVYDSFGAGHSSTSISAGLGMAVGRDL
LGKNNNVISVIGDGAMTAGQAYEAMNNAGFLDSNLIVVLNDNKQVSLPTA
TLDGPATPVGALSSALSKLQASPKFRQLREAAKSVTKQIGPEAHEIAAKV
DEYARGMVSATGASLFEELGLYYIGPVDGHNLEDLVTIFERVKSMPAAGP
VLIHIVTEKGKGYPPAEAAADKMHGVVKFDVKTGKQDKVKSPTLSYTQYF
AEALIKEAEADEKIVAIHAAMGGGTGLNYFQKQFPERCFDVGIAEQHAVT
FAAGMATEGLKPFCAIYSSFLQRGYDQVVHDVDLQKIPVRFAMDRAGLVG
ADGPTHCGAFDITYMACLPNMVVMAPSDEAELMHIVATAASIDDRPSCFR
FPRGNGIGAVLPPNNKGIPIEVGKGRILIEGTRVAILGYGSIVQQCLGAA
TMLKSHNISTTVADARFCKPLDADLIKRLAKEHEVLITVEEGSIGGFGSH
VAHYLCLNGLLDGPLKVNCPSLFVTNICLLSVWN

>NjTPS-100

MMHLPQSPTLTRTYYPSITASFRSHSGAKSATNATTAASVEEETKDGIRK
LFKKVEVSVSSYDTAWVAMIPCGDKIDSVVRPCFPECLDWLLNNQLHDGS
WGLTPHTHLLLKDTLSSTLASILALKRWGIGQNHINKGLHFMDLNFHYAI
DKNHHSPTGFDVIFPGMLDYAKHLDLKLPLQPILSDAIIRNRDLELKRCY
ESEAYERDAYLAYVSEGIGKSQDWEMVMKYQRKNGSLFNSPATTAAALTH
HLPSDSRLLTYIRQSLTIFGNAVPTVYPLDIPVRLCMIENLERLGIDWHF
KDEIQSVLDETYSYWIRGDEQIFIDVSTCARAFRLLRMNGYDVSSDPLTK
MAEENYCFHSPDMQLNRMSDALELYKASQIIITPDKPALRKLNSQSSDFL
RHTLSNDSSCSDKLSAYIRQEINDALNFPFYASLERIATKRNVEHYNVDH
SGTIRVLKTSYCSPNTGNTIFLKLAVEDFNFIQSVHREEIKHLESWVVEQ
KLDKLKFVRQKVAYSFFVVAATYYTPKLSDARMMMAKNSILAVTIDDFFD
VAGSRDEFLNLIQLAEKWDVDIESDCCSEEVGIIFSGIHDASTEIATKAF
TCQRRSVTNHIVQIWLDYLKAAFREAEWSRNKYVPSIDEYIENAHITFAL
GPISIPSLYVVGPELSEEVVHSLEFKKMFKLMSTCGRILNDIQSLERETK
QGKLNFISLLMIHGGGGGGITKEEAIDQAKKLIKTQREELLSLLLKKGSI
VPRDCKEVFWKMTKIVHLFYEADDGFISQHLMKALNDIIYEPMLVTLP

>NjTPS-101

MVTRFSSISTRKLSTALTILSSDKAMVAISDYNDFHKAAKRCILTHILGP
AAQKRLRSHRDTLIENMSKHLHDFSKNNPLEPVNFRHYFQSELFGLALKQ

SLGKDVESIYVEKLGTTFSREELFDIFVLEPLMGALDVDWRDFFPYLKWV
PNKAIEERVQKMHIRREAAMKTLIQQSKKCIDSGEKTDCFHEFLLSLEKP
LTENEVLMMLWEIIIETSDTMMVTTEWAMYELAKNPKQQEKLYQELQTVC
VSDKIIEEKLSQLPYLCAVFHETLRKYSPVPVIPLRYVHEDTEIGGYHVP
AGSEIAINIYGCNMDNKVWENPEDWNPERFLENKNNTMDLHVTMAFGGGK
RVCAGALQAFHISCVTIGRLIQEFKWKLADGEEENVDMLGLTTHKLHPMK
AIIEPRA

>NjTPS-102

MMFSSRYALPLGHNPLIRIPFSSSSPSHSSNFIASTHSLGAKGIHSNRIS
IFPSVAIKSINLDCTRVSQRINQSIEIDNDDYDEVEACKNPPPPEVEKCI
KEIKAILGRMEGGELTTSAYDTAMVALVKDINGNGNPQFPSCLEWICNNQ
MDDGSWGAPYIFLVSDRLINTLACVIALKSWNIHPNKILKGISFFNNNFD
KLANENEEHMLIGFEIVLPGLIQRARKLEIEVPNDSHPILKEIYAKRNLK
LSKIPEEIFHTEHTTLLFSTEGLEDLEMERVIKLQCPDGSMLYSPASTAH
AFIHTKDLKCLTYLTQVVDKFNGRVPYIYPVDLFARNWFIDQIQRLGISR
YFVAEIDELVSYVSRYWDPKRGAYSISNSPFHNIDDTALCFRNLRLHGYQ
ISPDAFWHFKYGDAFCCFPRQSSESVTAIFDLLRASQLQFPGEKVLDETK
NFSYKFLREREANNTLIDKWVLLKDLPGEVKYALDMPWYANLPRVESRFY
IEQYGGEEDVWIGKTLYQMKEVNNNTYLKLAKLDYNLCQEQHQLEWLHML
RWYKKNNLMEFGISERMVLLDYFLATANIFELERSNERLGWAKSSILLHI
VSTYINKHLTSNQQKLSFINTFKTHTIALHKSSNSNSVEERVISLVCKTL
YQQSVHVFNACGRDIHLPLFHAWEIWLMTWQEEDVNHVDSHSQSSVVLLA
TTINMFGGRLVTDQISSHPQYHRLTQLIAKICNHLHPHHVSDDEYCLDRK
KIITVEIESNMQELLQLVLQKSSDVDRINCNIKQTFLAIAKSAYYAAYCS
SATINMHVHKVLFQPVI

>NjTPS-103

MSTLTKPPPCPLLCISRTSSSPPTSQFLLPKPRKTSHRIAHHKIRNKYGN
FLDLKPEYHPEPLKFDLNWLDPASRFRADVIIIGAGPAGLRLAERISGYG
IKVICVDPSPLSMWPNNYGAWVDELETIGLDDCFDKKWGMATVCINDNKT
KYLDRPYGRINRKNLKLKLLESCVNNGVLFHKAKVWKVAHQELDNMLVCD
DGNELKASLIVDASGFASTFVEYDKPRNHGYQIAHGILAEVEEHPFDLDK
MLLMDWRDSHLGNEPNLRMNNAKLPTFLYAMPFDSNLIFLEETSLVSRPV
LPYIEIKKRMVARLRHLGIRVKKVIEEEKCVIPMGGSLPRIPQRIMGVGG
TAGLVHPSTGYMVARTLALAPVLGDAIAECLGSTRMIRGDPLSRRVWDGL
WPLDKRCVRDYYSFGMETLLKLDLKGTRGFFDAFFDLDPYYWHGFLSSRL
SLGELAMLSLSLFGHASNPSRVDVVTKCPVPLVKLIGNLALETI

>NjTPS-104

MADESKDWVLVLTAQTPTNIAVIKYWGKRDENLILAINDSISVTLDPDHL
CTTTSVAVSPSFDQDRMWLNGKEISLLGGRYQNCLREIRSRASDLEDEKK
GIKIAKKDWQNLRVHIASHNNFPTAAGLASSAAGFACLVFSLAKLMNVKE
DQGQLSAIARQGSGSACRSLYGGFVKWVMGKDEKGTDSIAVQLADEKHWD
ELVIIIAVVSSRQKETSSTSGMRDSVETSALIQHRAKEVVPKRIVEMEEA
IKNRDFPAFAHLTCADSNQFHAVCLDTYPPIFYMNDTSHKIISCVEKWNR
AEGTPQVAYTFDAGPNAVMIARDRKTAALLLQRLLFHFPPHSDTNLDSYV
IGDKSILKDAGIQDMKDIEDLPPPQEIKDNIRTTQKSKGDVSYFICTRPG
RGPVLLPETLSLLNPQTGLPK

>FhLin

MALLPCLPSQFPCSPVTGFVRLPLLSSRRSSKGVQSSNYRFRCCINTGTSVSQP
LRRAASYPQNIWDDSYIQALNCGYMGDEQVNEIRKLKEEVGQLFSDSKEILY
QIELIDELQQLGVAYHFQDEIKDKLSTIFCSLEKTSLFMENDLKATSLVFRLLRE
HGFHASADIFNNFRENKGNFKSCLKNDMEGMINLYEASFFAVEGENQLDEAR
VFATEHLRHLSESLVEASLRERVAHALELPLHFRMSRLHTRWFIDWYEKKVDK
NSNLCRLAKLDFNFVQNIYKRELKELSRWWTNLGLGQKLSFARDRLVENYLF
VIGWAFEPKLWQNREAMTMANCLVTTLDDIYDVYGSLDELELFTDAVNRWD
AEAIEQLPDYMKTCIMALFNTTNLTANKIMYSKGVNIIPQLRRSWADLCKA
YLVEAKWYHSGYMPTLEEYLDTAWISISGPVVLTQAYCTSENITDEALKCYNF
YPDVVRQSSMISRLWNDLATSTAEMERGDVPKSIQCYMHEKGVSEEVAREHI
RDMIVSISKKFDYDCISNSSIAESLKSVALDVHRMSQCVYQYEDGYGEQGHQ
KREQVISLLFEPIPL

>AmMyr

MIYIWICFYLQTTLLPCSLSTRTKFAICHNTSKLHRAAYKTSRWNIPGDVGSTP
PPSKLHQALCLNEHSLSCMAELPMDYEGKIKETRHLLHLKGENDPIESLIFVD
ATLRLGVNHHFQKEIEEILRKSYATMKSPIICEYHTLHEVSLFFRLMRQHGRYV
SADVFNNFKGESGRFKEELKRDTRGLVELYEAAQLSFEGERILDEAENFSRQIL
HGNLAGMEDNLRRSVGNKLRYPFHTSIARFTGRNYDDDLGGMYEWGKTLRE
LALMDLQVERSVYQEELLQVSKWWNELGLYKKLNLARNRPFEFYTWSMVIL
ADYINLSEQRVELTKSVAFIYLIDDIFDVYGTLDELIIFTEAVNKWDYSATDTLP
ENMKMCCMTLLDTINGTSQKIYEKHGYNPIDSLKTTWKSLCSAFLVEAKWSA
SGSLPSANEYLENEKVSSGVYYVVLVHLFCLMGLGGTSRGSIELNDTQELMSSI
AIIFRLWNDLGSAKNEHQNGKDGSYLNCYKKEHINLTAAQAHEHALELVAIE
WKRLNKESFNLNHDSVSSFKQAALNLARMVPLMYSYDHNQRGPVLEEYVKF

MLSD

>Cstps1

MSSGETFRPTADFHPSLWRNHFLKGASDFKTVDHTATQERHEALKEEVRRMIT
DAEDKPVQKLRLIDEVQRLGVAYHFEKEIGDAIQKLCPIYIDSNRADLHTVSLH
FRLLRQQGIKISCDVFEKFKDDEGRFKSSLINDVQGMLSLYEAAYMAVRGEHI
LDEAIAFTTTHLKSLVAQDHVTPKLAEQINHALYRPLRKTLPRLEARYFMSMI
NSTSDHLCNKTLLNFAKLDFNILLELHKEELNELTKWWKDLDFTTKLPYARD
RLVELYFWDLGTYFEPQYAFGRKIMTQLNYILSIIDDTYDAYGTLEELSLFTEA
VQRWNIEAVDMLPEYMKLIYRTLLDAFNEIEEDMAKQGRSHCVRYAKEENQK
VIGAYSVQAKWFSEGYVPTIEEYMPIALTSCAYTFVITNSFLGMGDFAT
KEVFEWISNNPKVVKAASVICRLMDDMQGHEFEQKRGHVASAIECYTKQHG
VSKEEAIKMFEEEVANAWKDINEELMMKPTVVARPLLGTILNLARAIDFIYKE
DDGYTHSYLIKDQIASVLGDHVPF

>OsCyc2

MQMQVLTAASSLPRATLLRPAAAEPWRQSFLQLQARPIQRPGIMLHCKAQLQ
GQETRERRQLDDDEHARPPQDGDDDVAASTSELPYMIESIKSKLRAARNSLGE
TTVSAYDTAWIALVNRLDGGGERSPQFPEAIDWIARNQLPDGSWGDAGMFIV
QDRLINTLGCVVALATWGVHEEQRARGLAYIQDNLWRLGEDDEEWMMVGF
EITFPVLLEKAKNLGLDINYDDPALQDIYAKRQLKLAKIPREALHARPTTLLHS
LEGMENLDWERLLQFKCPAGSLHSSPAASAYALSETGDKELLEYLETAINNFD
GGAPCTYPVDNFDRLWSVDRLRRLGISRYFTSEIEEYLEYAYRHLSPDGMSYG
GLCPVKDIDDTAMAFRLLRLHGYNVSSSVFNHFEKDGEYFCFAGQSSQSLTA
M
YNSYRASQIVFPGDDDGLEQLRAYCRAFLEERRATGNLMDKWVIANGLPQRG
RVRAGFPMEGKLAASRDEGVSGAVRRYEDAWIGKGLYRMTLVNNDLYLEAA
KADFTNFQRLSRLEWLSLKRWYIRNNLQAHGVTEQSVLRAYFLAAANIFEPN
RAVGTLGWARTAILAEAIASHLRQYSANDAADGMTERLISGLASHDWDWRES
KDSAARSLLYALDELIDLHAFGNASNSLREAWKQWLMSWTNESQGSTGGDT
ALLLVRTIEICSGRHGSAEQSLKNSADYARLEQIASSMCSKLATKILAQNGGSM
DNVEGIDQEVDVEMKELIQRVYGSSSNDVSSVTRQTFLDVVKSFCYVAHCSPE
TIDGHISKVLFEDVN

>OsDTC2/KS8

MMLLSSSYSGGQFPGVSPLGTRPKRSTTVVPRPVVTRATAGGVRNNLEVVGN
AGTLQGMDIDELRVIVRKQLQGVELSPSSYDTAWVAMVPVQGSRQSPCFPQC

VEWILQNQQEDGSWGHSAGPSGEVNKDILLSTLACVLALNIWNVGQDHIRRG
LSFIGRNFSVAIDGQCAAPVGFNITFSGMLRLAIGMGLKFPVMETDIDSIFRLRE
VEFERDAGGTASARKAFMAYVSEGLGREQDWDHVMAYQRKNGSLFNSPSTT
AASAIHSCNDRALDYLVSLTSKLGGPVPAIYPDKVYSQLCMVDTLEKMGISSD
FACDIRDILDMTYSCWMQDEEEIMLDMATCAKAFRLLRMHGYDVSSEGMAR
FAERSSFDDSIHAYLNDTKPLLELYKSSQVHFLEEDFILENIGSWSAKLLKQQL
SFNKISKSLMPEVEYALKYPFYATVEVLEHKGNIERFNVNGFQRLKSGYCGSG
ADKEILALAVNKFHYAQSVYQQELRYLESWVAEFRLDELKFARVIPLQSLLSA
VVPLFPCELSDARIAWSQNAILTAVVDDLFDGGGSMEEMLNLVALFDKWDDH
GEIGFCSSNVEIMFNAVYNTTKRIGAKAALVQKRCVIDHIAEQWQVMVRAML
TEAEWAAGKHIPATMGEYMSVAEPSFALGPIVPVSAYLLGEELPEEAVRSPEYG
RLLGLASAVGRLLNDVMTYEKEMGTGKLNSVVLLQPLAAGGAASRGGGGAP
APAPASVEAARAEVRRAIQASWRDLHGLVFGSGGGSSSSIIPRPCREVFWHTG
KVASVFYQEGDGYARKAMRSMANAVILEPLHLQE

>SlTPS38

MLQSCISTMDIRRSGNYKPSIWEDGYVQSRPNLYAEEKYCERAEKLKEEVRK
MLQKRMTNSLEQLELVDI
LQRLGIYYHFEEEIDTVLKQIYVNYNKRDHHNEELYDTALEFRLLRQHGYHLP
QEIFCSFMNEEGKFKTA
LVEDTKGLLSLYEASYLCMEDENIMENARDFATHYLMENVKKKMDEQVSHA
LEMPVHWRMERLEARWFIE
IYHKKENMNPLLLELAKLDYNMVQATYLEELKQMSRWDKNMKLVKKMSFV
RDRLVEGFFWAVGFTPNPQF
GYCRKLSTKLSVLLTTIDDIYDVYGTLDELELFTDIVDRWDINAIEQLPEYMKI
SFLALFNSMNELAYDI
LKEQGFSIISHIRKQWANLCKAYLLEVKWYQRGYTPSLDEFLRNAWITNTGPV
LIMHAYFCITNPIKEDE
LQRLNHYPAIIYSPSLILRLANDLATSPDEIKKGDYLKSIQCYMHDSKSCEENA
RNYIKKLIDETWKKMN
RDILRDESLSKDFRRTSMNLARIAQCMYQHGDGFGIPDRETKDRILSLFFQPIPL
T

>VvaTerP

MALSMLSSIPNLITHTRLPIIIKSSSCKASPRGIKVKIGNSNCEEIIVRRTANYHPT
IWDYDYVQSLRSDYVGETYTRRLDKLKRDVKPMLGKVKKPLDQLELIDVLQ
RLGIYYHFKDEIKRILNSIYNQYNRHEEWQKDDLYATALEFRLLRQHGYDVPQ
DVFSRFKDDTGSFKACLCEDMKGMLCLYEASYLCVQGESTMEQARDFAHRH
LGKGLEQNIDQNLAIEVKHALELPLHWRMPRLEARWFIDVYEKRQDMNPILL

EFAKLDFNMVQATHQEDLRHMSSWWSSTRLGEKLNFARDRLMENFLWTVG
VIFEPQYGYCRRMSTKVNTLITIIDDVYDVYGTMDELELFTDVVDRWDINAM
DPLPEYMKLCFLALYNSTNEMAYDALKEHGLHIVSYLRKAWSDLCKSYLLEA
KWYYSRYTPSLQEYISNSWISISGPVILVHAYFLVANPITKEALQSLERYHNIIR
WSSMILRLSDDLGTSLDELKRGDVPKSIQCYMYETGASEEDARKHTSYLIGET
WKKLNEDGAVESPFPETFIGIAMNLARMAQCMYQHGDGHGIEYGETEDRVLS
LLVEPIPSLSFE

>PcAFS1

MEFRVHLHADHEQKILQNQMKPEPEASYLINQRRSANYKPNIWKNDFLDQSL
ISKYDEDQYRKLSEKLIEEVKIYISAETKDLVAKFELIDSVRKLGLANHFEKEIK
EALDSIAAIESDNLGTRDDLYGTALHFKILRQHGYKVSQDIFGRFMDEKGTLE
NHHFAHLKGMLELFEASNLGFEGEDILDEAKASLTLALRDSGHICYPDSNLSR
DVIHSLELPSHRRVQWFDVKWQINAYEKDICRVNATLLELAKLNFNMVQAQL
QKDLREASKWWANLGIADNLKFARDRLVECFACAVGVAFEPEYSSFRICLTKV
INLVLIIDDVYDIYGSEEELKHFTNAVDRWDSRETEQLPECMKMCFQVLYNTT
CEIAHEIEKENGWNQVLPQLTKVWADFCKALLVEAEWYNKSHIPTLEEYLRN
GCNSSSVSILLVHSFFSITHEGTKEMADFLHKNEDLLYNLSLIVRLNNDLGTSA
AEQERGDSPSSIVCYMREVNASEEIARKNIKGMIDNAWKKVNGKCFTTNQVP
FLSSFMNNATNMARVAHSLYKDGDGFGDQEKGPRTHILSLLFQPLVN

>AtTPS14

MALIATKISSRSCFVSAYPNNSPTFLISKFPNTVDSLSPANTAKRSILRNVHASVS
NPSKQFHNKTSLEYLHELNIKKIKNILSANVDVPSENLEMIDVIQSLGIDLHFR
QEIEQTLHMIYKEGLQFNGDLHEIALRFRLLRQEGHYVQEIIFKNILDKKGGFK
DVVKNDVKGLTELFEASELRVEGEETLDGAREFTYSRLNELCSGRESHQKQEI
MKSLAQPRHKTVRGLTSKRFTSMIKIAGQEDPEWLQSLLRVAEIDSIRLKSLTQ
GEMSQTFKWWTELGLEKDVEKARSQPLKWHTWSMKILQDPTLTEQRLDLTK
PISLVYVIDDIFDVYGELEELTIFTRVVERWDHKGLKTLPKYMRVCFEALDMIT
TEISMKIYKSHGWNPTYALRQSWASLCKAFLVEAKWFNSGYLPTTEEYMKNG
VVSSGVHLVMLHAYILLGEELTKEKVELIESNPGIVSSAATILRLWDDLGSAKD
ENQDGTDGSYVECYLNEYKGSTVDEARTHVAQKISRAWKRLNRECLNPCPFS
RSFSKACLNIARTVPLMYSYDDDQRLPDEYLKSLM

>GmNES

MDNIYIKQALVLKEVKHVFQKLIGEDPMESMYMVDTIQRLGIEHHFEEEIEAA
LQKQHLIFSSHLSDFANNHKLCEVALPFRLLRQRGHYVLADVFDNLKSNKKEF

REKHGEDVKGLISLYEATQLGIEGEDSLDDAGYLCHQLLHAWLTRHEEHNEA
MYVAKTLQHPLHYDLSRFRDDTSILLNDFKTKREWECLEELAEINSSIVRFV
NQNEITQVYKWWKDLGLNNEVKFARYQPLKWYMWPMACFTDPRFSEQRIEL
TKPISLVYIIDDIFDVYGTLDQLTLFTDAIKRWELASTEQLPDFMKMCLRVLYEI
TNDFAEKICKKHGFNPIETLKRSWVRLLNAFLEEAHWLNSGHLPRSAEYLNN
GIVSTGVHVVLVHSFFLMDYSINNEIVAIVDNVPQIIHSVAKILRLSDDLEGAKS
EDQNGLDGSYIDCYMNEHQDVSAGDAQRHVAHLISCEWKRLNREILTQNQLP
SSFTNFCLNAARMVPLMYHYRSNPGLSTLQEHVKLLSNNAVAGAERHVVHIL
CLQFVIE

>MdGDS-RG1

MSSVEVPLSQSSSPNDTFDPKPSATFSPSIWGDHFLSYASLEVDAELEQHVQEL
KEEVRSMLMTSPENVSQKLNLIDDIQRLGVSYHFGNEIEEILQKIHQSSYDLDD
LYTVALRFRLLRQQGYNVSCDLFNKLKDGDGKFKESLVDDVVGLLSLYEATH
LRIHGEEILDEALTFTTTNLESATFRLSPPLAKAVTHALNQPLRKGLPRVEARY
YLSVYQELRESPNETLLTFAKLDFNRLQRVHQKELSEITRWWKDLDVPNKLPF
ARDRLVEVYFCWSMSVYFQPQYSFARRTSCKVTAITSIMDDIYDTHGKFEELE
LFTEAIERWDVSAIDQLPEYMKLCYRELLNIYSEIHEKLAHEGKLYRIDHAREA
MKNQVRGFFDEAKLFRQNHMPSLDEYMSVSLMTCGYALLITTSFVGMEEATI
DSFDWLLTSPQAVKAASTVTRLMDDIADHKLEQEREHFASAVNCYMRKYGAT
EEEAIIELQRQVNNAWKDINEACLHPTAVAMPLLIRILNFARVMDVVYKCEDG
YNNADGGLKDFIVSTLVEPVAL

>SlTPS12

MASSSANKCRPLANFHPTVWGYHFLSYTHEITNQEKVEVDEYKETIRKMLVE
APEGSEQKLVLIDAMQRLGVAYHFDNEIETSIQNIFDASSKQNDNDNNLYVVS
LRFRLVRQQGHYMSSDVFKQFINQDGKFKETLTNDVQGLLSLYEASHLRVRD
EEILEEALTFTTTHLESTVSNLSNNNSLKAEVTEAFSQPIRMTLPRVGARKYISI
YENNDAHNHLLLKFAKLDFNMLQKLHQRELSDLTRWWKDLDFANKYPYAR
DRLVECYFWILGVYFEPKYSRARKMMTKVIQMASFFDDTFDAYATFDELEPF
NNAIQRWDINAIDSVPPYLRHAYQALLDIYSEMEQALAKEFKSDRVYYAKYE
MKKLVRAYFKEAQWLNNDNHIPKYEEHMENAMVSAGYMMGATTCLVGVEE
FISKETFEWMINEPLIVRASSLIARAMDDIVGHEVEQQREHGASLIECYMKDY
GVSKQEAYVKFQKEVTNGWMDINREFFCPDVEVPKFVLERVLNFTRVINTLY
KEKDEYTNSKGKFKNMIISLLVESVEI

>SlTPS41

MRNLREEIKNMLSSMGDGRSSVSPYDTAWVSFIEDTNTNINGTSKRPLFPSCL
QWIIDNQLDDGSWGEELVFCIYDRLLNTLACVVALTLWNTCLHKRNKGVMFI
KENLRKLEGGEVVNMTSGFEFVFPSLLDKAQQLHIDNIPYDAPVFRDIYARRE
VKFTRFPKDLIHTIPTIVLFSLEGLRDLDWQRLLKLQMEDGSFLTSPSSTAIVFM
NTNDDKCFTFLQNAVQKFNGGVPCSYPADIQARLWAIDRLQRLGISYYFEEEI
KDLLEYVFRYWDKENGFFSARNSNICEVDTTCMAIRLLRLHGFDVSPDVLHK
FKDGDEFFCLRGESNKSATVMFNLYRCSQALFPGEIICEEAKNFTYNFLHQYL
ANNQSKDKWVIAKDIPGEIRYALEFQWYASLPRVESRLYIDQYGGADEIWISK
TLYRMPDVSNNVYLEAAKLDYNRCQSQHRFEWLIMQEWFEKGNFQKFGISK
KEVLVSFFLAASSIFEVEKSRQRLAWAKSCILCKMITSYINQEATTWNSFLMEF
KNYRDMSIKKSNETKEIIVLNNLCQFLHQLTKETYQDLGKDIHHQLHNVWEE
WLEENNTTCQEAAVLLVQTINLSSGHMTHDEILSKYTNKVCHMLNEFQNDQI
CNSSKARDIELHMQALVKLVFSNTSSNNINQGIEDTYFKVVKTFYYTAHVSEE
TINNHISKVLFQKA

>LnTPS3

MESIKDLVSEMKEEMFSSAVSLCSFVSSSAYDTAWLALIPDPARPGQPLFRQCL
EWIMKEQKEEGFWGEGGSIECLPASLACMVALQTWEAGPCNVGRGLAFVHA
NVEKLLREEDGAFPRWFCIVFPAMVELAQTKGLNVLPDGLTDVVASIFHQRER
FLEMEASFCEAYHPPLLAYLEALPPSYGHIGHEIILQNQNKDGSLFHSPSATAH
AFMVTGNEGFKLYLESMVQRCGHGGVPPFYPIDEELIKLSIVDRLERLGLSEH
FSVEIEDVLCHVYRNWTVQEKEEDENERSTLVPIRLYKDSLAFRLLRMHGYRV
SPRRFCWFLQHEDITSYIEENYNCLLSTLLNVYRATDVTFLGENELEEARSFSR
TLLERGITMKTAADSIVTFNIQREIEHQLGLPWLARLDHLEHRDSIEGSKSSDY
LWIGKACYYRISCLNDDKLLQLARENYTLRQSIFKNELEEVTRWSRDSGLRDI
GFGREKTTYCYLAVASSVYHPSLSDVRMLVCKSAILVTVADDFFDMEGSLDEL
NTLTEAVGRWNGDGLSGHGKVIFDALDELADNTIKMLFDRHRVDATESIREL
WYETFVSWLKEAKWSRRRYAPSIDDYIKTSMISVAVHTMILPACYLINPGFAM
HRMRHSKTDVITRLLMISARLLNDMQSYEKEKEDGKLNLVLLYSKENLELDIE
NATDCIEKILDEQKKEFLKMTLEDGITDLPKQWRHLHLGGLKAFQMFFNSSN
GFDSPTAMIKNINKAFYEPLVTDTWKTTPQQTLQPHPGSKNCNSIVRAHIGKS
CQDLGRRRLLYYSNIETSGIRVLPVHNFPLRFPIIRLSHKFYAKVLMPPSVTKPL
LW

>PdTPS

MEESLKCLIERVKEEVFSPSADMYSFLPSSAYETAWVAMIPNPERCRRPMFPNC

LVWMLRTQNDGGLWGELDLTIDCLTATLACIIALKIWNVGYINIEKGLKFLHA
NTEKVLMKHHGGIPRWFAIIFPGMLELAKAKGLEVFPQGYTGVVEDIFNEREK
IFKMEETSCGGHHLPLSLYLEALPAIYRGKHEDILKHKREDGSLFHSPSATACA
FMITGDGDCKEYLEAMVQRCGHGVPPAYPVDQDLIKLCMMDHLMRLGCGE
HFTKEIGDAMDHIYWNWVTEELQPSNVHNLPLQIFKDFLTFQLLRRHGHRISP
ERCCWFMRDPQTLLHIEENYQNFLGAMYAVYIATHLMFLEEPEFENAKTFSK
KILQKGLPGKDVKDHPSALSDLQKEIEHELKHLWLARMDHLEHRTYIERTK
GYNIWIGKSSSCRLTCSDDIIQLAIKNFMTRQSVYRTELEELKRWSKDTGLAN
MGFGRENTSYCYFVAATPISLLLDSEVRKIMAKSATLVTVADDFFDEHGSLDEL
QCLTEAVQRWEGEGLSGHSKVIFETLDDLVCDIALKIFNQQGHDMKTLLQDF
WREAFNVWLTESEWSRIKHAPSIDEYLRVGTLSIAIQAMYLPACYLASPKH
PQSSPETHYSKITTLLMLSARLLNDIQSYEKEMKDGKLNMVPLYLKENPQANI
EDAIAYIQNKLERHKKQLLEITMVDINTEVPKEWKQIHLCALKGFQMLFNTFN
AFDSPTALLQDITMAFYEPLVMDSQRILLPTFVLNAHESIKETTGRSMEESSKK
ESSVKAQGQLQGVNGKIVYNPRAITSKIKQSLYYVRPKSFITLRCPSSYMFLAV
PPISPSPLLKM

>NjTPS3

MDSYLNASSAPPPKKNMQEPVRPIANYHPSVWGNQFLKYASNPKQSDSGA
EEQHEQLKEALRKKLVVNVANERAGEQLKLIDAIQRLGVAYQFENEIDVV
LNNQLQLLNNEDDDLHMVSLRFRLLRQHGHNVSCGVFGKFKDIEGRFKEC
LMDDVRGLLSLYESTHMRVHKEDILEEALEFTTTHLEQVVKSPLSGSVLA
SQVVHALNMPIRKGLTRIEARHFIPIYQQDESHDETLLKFAKLDFNMLQK
VHQREVADITMWWKDLNVSEKLPYARDRAVECYFWILGVYFEPQYSRARR
ILTKVICMTSLIDDTYDSYGTFEELILFTDAIQRWDVNAKNQLPEYMRHI
FGELLDVYGAMEEELSKEGISYRVDYAKQIMIQLVTAYNHEAIWYHDGYV
PTLEEYLEVALVSCGYIMAATTSFVGMGVKAVPKQAFDWVSSNPLMVQASSI
INRLTDDRVGHELEQQRGHVASGVECYMKQHNATEEEVLVEFNKRITS
AWKDMNQECLHPFPVPIHLLERVLNLARFMNIFYKDEDCYTHSNTRMKGI
ITSILIESIPS

>NjTPS15

MSIIIATNGTEHPIFRPLANFPPSLWGNLFTSFSMDNQAREIYAKEHEGL
KEKVRVMLLDTTNYKISEKINFINTVERLGVSYHFEKEIEELLHQMFDAH
SKLLDDIQEFDLFTLGIYFRILRQHGYKISCDVFNKLKDSNGEFKDELKD
DVNGMLSLYEATHVRTHGENILDEALIYTKAQLESMAAASLSPFLAKQVK
HALMQALHKGIPRIEARNYISVYEEDPNKNDLLLRFSKIDFNLVQMIHKQ
ELCDTFRWWKDLEFESKLSFARNRVVEAYLWTLSAYYEPKYSSARIILVK
LMVIISVTDDTYDAYGTLDELQLFTDAVQRLDMSSINQLPDYMKTIYKAL

LDLFDEIEDRLSKHETDHSYRVAYAKYVYKEIVRCYDMEYKWFNKNYVPA
FEEYMQKALVTSGNRLLITFSFLGMDEVATIQAFEWVKSNAKMIVSSNKV
LRLIDDIMSHEEEDERGHVATGIECFVKEHGLTREEVIVEFHKRIDDAWK
DINEEFITPNNLPIEILTRVLNLTRIGDVVYKYDDGYTHPEKALKDHIIS
LFVDPVSI

>NjTPS21

MDSYLNASSAPPPKKNMQEPVRPIANYHPSVWGNLFLKYASNPEVKQSDG
GAEEQHEQLKEALRKKLVVNVANERAGEQLKLIDAIQRLGVAYHFETEID
VILNNQLQLLNNQDDDLHMVSLRFRLLRQHGHNVSCGVFGKFKDIEGRFK
ECLMDDVRGLLSLYESTHMRVHKEEILEEALEFTTAHLEQVDKSPLSGSV
LASQVVHALNMPIRKGLTRIEARHFIPIYQQDESHDETLLKFAKLDFNML
QKVHQREVADATMWWKDLNVSEKLPYARDRVVECYFWILGVYFEPKYSRA
RRMLTKVICLASLIDDTYDSYGTFEELILFTDAIERWDVNAKNQLPEYMR
HIFEELLDVYGAMEEELSKEGISYRVDYAKQIMKQLVTAYNHEAIWYHDG
YVPTLEEYLEVALVSCGYMMLATTSFVGMGVTAVTKQALDWVSSKPLMVQ
ASSIINRLADDKVGHEFEQQRGHVVSGVECYMKQHNATKEEVLVEFNRRI
TSAWKDMNQECLHPLPVPMHLLERVLNLACFMNIFYKDEDCYTHSNTRMK
DFITSLLIESVPS

>NjTPS28

MSCMRSISSPSQLLSKSYDNIIDSSSSSSRSFSWSFKSTNPAAAARTSMC
LSSRSSPSAVVVPPTSNNGSFLKYLQQSTVLVPQEIDDNSRTMELIEETR
KELVKVREPVEKMRLIEALQRLGISYHFENEINIILENLSAGGGRHSDED
LFTTSLRFRLLRHNGHHISNDVFEKFVDENGKFKESLKEDTMGMLSLYEA
SYMGANGEDILLQAMEFTKNHLKESLPLMESNLGKQVLQSLELPKNLRMA
RLEARRYIEEYSNESDHNLALLELAKLDYNQVQSLHQMELAEISRWWKHL
GLVDKLSFARDRPLECFLWTVGILPEPKDSGCRIELAKTIAILLVIDDIF
DTHGSYDELVLFTNAIRRWDLNAMEELPEYMKICYMALYNTTNEICYKVL
KENGWSVLPFLKTTWIDMIEGFMVEAKWLNNEEVPNLEEYIENGVTTAGS
YMALVHIFFLIGEGVNEDNVKLLLNPYPKLFSSAGRILRLWDDLGTSKEE
QERGDVASSIQLFMKENNITCEEEARNQIIQIVQNLWKELNGELMAPNAL
PLPIIKACLNMARASQVVYQHDGDSYFSNVDNYVQSCFIHQFVCNAFLVI
IFFYIYTFYIYLFFHINKFMHID

>NjTPS35

MDRSIQASSAPLPLSVLEPTRPIANFHPTIWGNYFLKFASDPGTNDDSDI

NQQIAQMKEDVRKMIVNSGDRREQQLKLIDEIQRLGVSYHFKSEIDVVLN
DHLLTLNDNGDDLYMESLRFRLLRQHGHNVSCDVFEKFKDGEGRFKEYLT
DDVRGLLSLYEATHMRVHKEEILDEALEFTTSHLEQVVKYSLSDHVLASK
VVHALKMPIRKGLTRLEARHYIPIYQMDNSHDETLLKFAKLDFNKLQKLH
QSELGDMTRWWKDFNVAEKLPYARDRFVECYFWALGVYFEPQYSHARRML
TKVIAFISLIDDTYDSYGTFEELSLFTDAIQRWDVNAKNQLPEYMRHIYG
ELLDVYNAMEEELSKEGISYRIDYAKQTMKQQVGTYFDEAIWYNNGYVPT
MEEYLKVALVSCGYIMLSTTSFVGMGVSVVPNQAFDWVTSNPLIVEASSV
INRLSDDKVGHKIEQERGHVVSAVECYMKQHNRTEEETIAEFKKRVTTAW
KDMNQECLHPIAVPMHLLERVLNFARFMNVFYEDEDCYTNSKSRMKDCIT
SLLVESIPI

>NjTPS48

MDSYLNASSAPPPKKNMKEPVRPIANYHPSVWGNQFLKYASNPKQSDGGA
EEQHEQLKEALRKKLVVNVANERAGEQLTLIDAIQRLGVAYQFETEIDVV
LNNQLQLLNNQDDDLHMVSLRFRLLRQHGHNVSCGVFGKFKDIEGRFKEC
LMDDVRGLLSLYESTHMRLHKEDILEEALEFTTTHLEQVVKSPLSGSVLA
SQVVHALNMPIRKGLTRIEARHFIPIYQQDESHDETLLKFAKLDFNMLQK
VHQREVADITMWWKDLNVSEKLPYARDRAVECYFWILGVYFEPQYSRARR
ILTKVICMTSLIDDTYDSYGTFEELILFTDAIQRWDVNAKNQLPEYMRHI
FGELLDIYGAMEEELSKEGISYRVDYAKQIMIQLVTAYNHEAIWYHDGYV
PTLEEYLEVALVSCGYIMAATTSFVGMGVTAVPKQAFDWVSSNPLMVQAS
SIINRLTDDRVGHEVCI
